# Adaptive resistance to lorlatinib via EGFR signaling in ALK-rearranged lung cancer

**DOI:** 10.1038/s41698-023-00350-7

**Published:** 2023-01-26

**Authors:** Yuki Katayama, Tadaaki Yamada, Keiko Tanimura, Shinsaku Tokuda, Kenji Morimoto, Soichi Hirai, Yohei Matsui, Ryota Nakamura, Masaki Ishida, Hayato Kawachi, Kazue Yoneda, Kazutaka Hosoya, Takahiro Tsuji, Hiroaki Ozasa, Akihiro Yoshimura, Masahiro Iwasaku, Young Hak Kim, Mano Horinaka, Toshiyuki Sakai, Takahiro Utsumi, Shinsuke Shiotsu, Takayuki Takeda, Ryohei Katayama, Koichi Takayama

**Affiliations:** 1 Department of Pulmonary Medicine, Graduate School of Medical Science, Kyoto Prefectural University of Medicine, Kyoto, Japan; 2 Second Department of Surgery, University of Occupational and Environmental Health, Kitakyushu, Japan; 3 Department of Respiratory Medicine, Graduate School of Medicine, Kyoto University, Kyoto, Japan; 4 Department of Drug Discovery Medicine, Graduate School of Medical Science, Kyoto Prefectural University of Medicine, Kyoto, Japan; 5 Division of Experimental Chemotherapy, Cancer Chemotherapy Center, Japanese Foundation for Cancer Research, Tokyo, Japan; 6 Department of Respiratory Medicine, Graduate School of Medical Sciences, Kyushu University, Fukuoka, Japan; 7 Department of Respiratory Medicine, Japanese Red Cross Kyoto Daiichi Hospital, Kyoto, Japan; 8 Department of Respiratory Medicine, Japanese Red Cross Kyoto Daini Hospital, Kyoto, Japan

**Keywords:** Non-small-cell lung cancer, Cancer therapeutic resistance, Targeted therapies

## Abstract

Anaplastic lymphoma kinase (ALK)-tyrosine kinase inhibitors rarely elicit complete responses in patients with advanced ALK-rearranged non-small cell lung cancer (NSCLC), as a small population of tumor cells survives due to adaptive resistance. Therefore, we focused on the mechanisms underlying adaptive resistance to lorlatinib and therapeutic strategies required to overcome them. We found that epidermal growth factor receptor (EGFR) signaling was involved in the adaptive resistance to lorlatinib in ALK-rearranged NSCLC, activation of which was induced by heparin-binding EGF-like growth factor production via c-Jun activation. EGFR inhibition halted ALK-rearranged lung cancer cell proliferation by enhancing ALK inhibition-induced apoptosis via suppression of Bcl-xL. Xenograft models showed that the combination of EGFR inhibitor and lorlatinib considerably suppressed tumor regrowth following cessation of these treatments. This study provides new insights regarding tumor evolution due to EGFR signaling after lorlatinib treatment and the development of combined therapeutic strategies for ALK-rearranged lung cancer.

## Introduction

Recently, the development of molecule-targeted therapy has markedly improved the clinical outcomes in patients with non-small cell lung cancer (NSCLC) harboring driver oncogenes^1^. Anaplastic lymphoma kinase (ALK)-rearranged genes are found in 3–5% of patients with NSCLC^2^. Several ALK-tyrosine kinase inhibitors (ALK-TKIs) have been developed, which have improved the clinical outcomes of patients with advanced ALK-rearranged lung cancer^3^.

Lorlatinib is a third generation ALK inhibitor that has been found to be more potent than second-generation inhibitors in biochemical and cellular assays^4^. A phase 3 trial of untreated ALK-rearranged NSCLC (CROWN study) demonstrated that the outcomes with lorlatinib were more favorable than those with crizotinib, a first-generation ALK-TKI^5^. Because of its efficacy and safety, lorlatinib has been approved as a standard first-line treatment option for untreated ALK-positive patients in some countries. Lorlatinib is also known to cover the broadest range of single ALK-resistant mutations^4–7^ and has demonstrated potent antitumor activity for patients with acquired resistance to previous ALK-TKI therapy, including first and second-generation ALK-TKIs^8,9^. However, nearly all cases of tumor relapse are believed to be due to the acquisition of resistance to lorlatinib after various durations of therapeutic interventions.

So far, multiple molecular mechanisms of acquired resistance to ALK inhibitors have been identified. Compound mutations in the ALK domain, including L1196M/D1203N, F1174L/G1202R, and C1156Y/G1269A, have been reported to be involved in “on target” acquired resistance to lorlatinib^10^. Treatment with sequential ALK inhibitors has been shown to induce compound ALK mutations that confer high-level resistance to ALK-targeted therapies^11^. In addition, various ALK-independent mechanisms underlying acquired resistance to ALK-TKIs have been identified, including bypass tract activations through EGFR, HER2/HER3, IGF-1R, MET, or RAF-MEK as mechanisms of “off target” resistance, epithelial–mesenchymal transition, and small cell and squamous cell lung cancer transformations as “phenotypic changes” of acquired resistance^3^. Various durations of therapeutic intervention have promoted the evolution of intratumor heterogeneity for advanced lung cancer^12^. Clinically, overcoming the acquired resistance to ALK-TKIs is challenging, because of the presence of individual mechanisms of drug resistance. Therefore, understanding the molecular mechanisms underlying intrinsic resistance and early refractoriness to lorlatinib is critical for establishing novel therapeutic strategies in patients with ALK-rearranged NSCLC. One of the potential therapeutic targets for solving such clinical issues involves the prevention of the drug-tolerant state, which is the basis for acquired resistance to molecular targeted agents in NSCLC tumors^13^.

Recent preclinical studies have revealed promising targets for overcoming the drug tolerance of ALK-rearranged NSCLC cells. Tsuji et al. indicated that ALK-TKIs enhance the expression of anti-apoptotic factors mediated via YAP1 upregulation in ALK-rearranged NSCLC cells^14^. Yanagimura et al. indicated that inhibition of the adaptive survival via STAT3 combined with an ALK-TKI may improve the outcomes of ALK-rearranged lung cancer^15^. Velde et al. showed that diverse epigenetic alterations, including those in EGFR, HER2, FGFR, AXL, EPHA2, cytokines, extracellular matrix (ECM), and ECM receptors affect the drug tolerance in ALK-rearranged lung cancer^16^. In addition, our previous reports have demonstrated that the inhibition of the tolerant signal via HER3 and c-Jun N-terminal kinase (JNK)/c-Jun in combination with ALK-TKIs dramatically improved the outcome of patients with ALK-rearranged NSCLC^17,18^. However, the molecular mechanism underlying the adaptive resistance to the new class of ALK-TKI, such as lorlatinib, in lung cancer cells remains unclear and requires further investigation.

Here, we aimed to elucidate the molecular mechanisms underlying the maintenance of cell survival during the initial phase of lorlatinib treatment and to establish promising novel therapeutic strategies for overcoming adaptive resistance.

## Results

### EGFR plays a pivotal role in the survival of ALK-rearranged NSCLC cells treated with lorlatinib

**Fig. 1:**
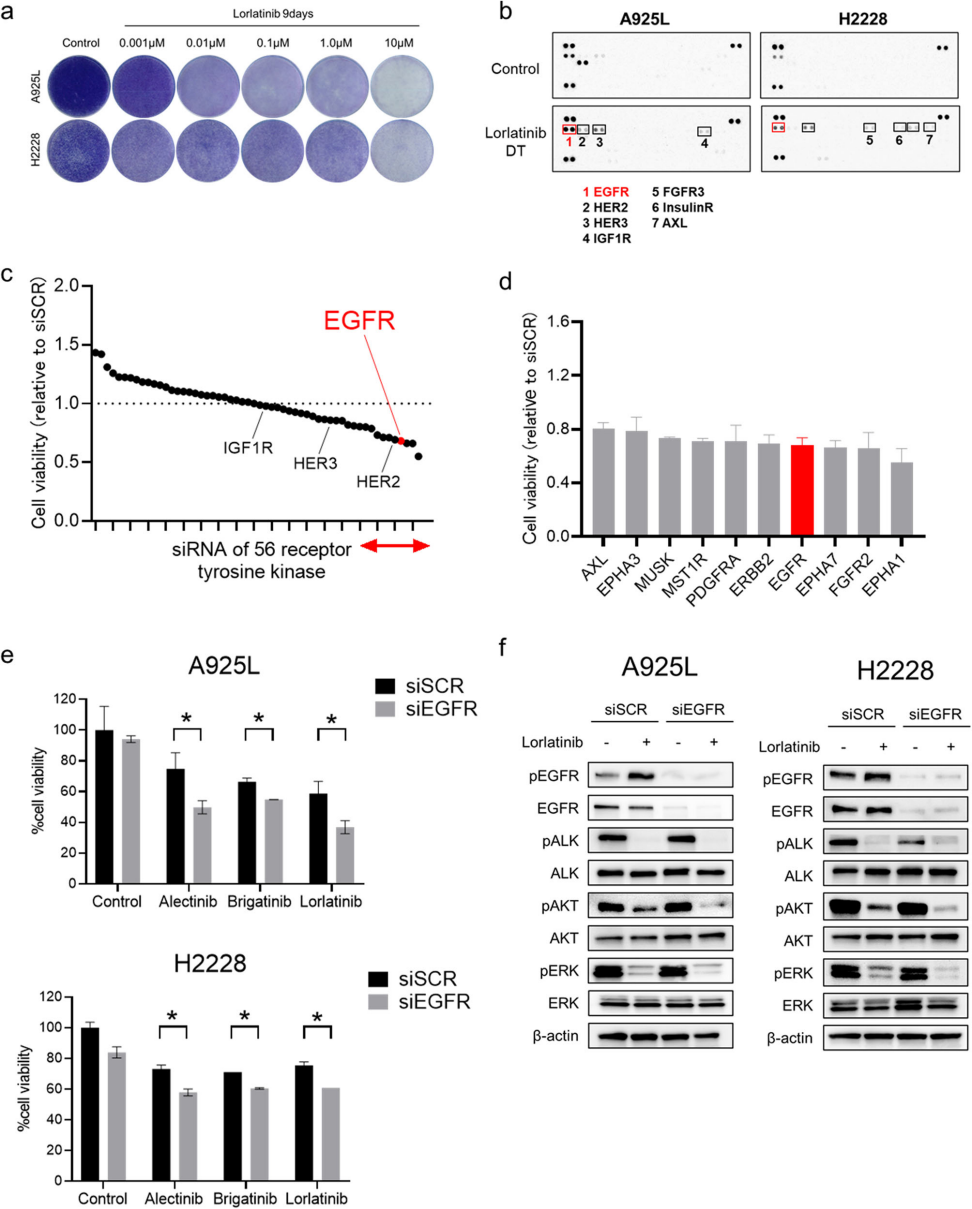
EGFR signaling plays a pivotal role in the adaptive resistance of ALK-rearranged NSCLC cells. **a** A925L and H2228 cells were visualized after crystal violet staining following 9 days of treatment with the indicated concentrations of lorlatinib. The drugs were replenished every 72 h. **b** Human phospho-RTK array analysis of parental cells and DT cells in A925L and H2228 cells. **c**, **d** Effect of a combination of lorlatinib (100 nmol/L) and knockdown of 56 receptor tyrosine kinases (RTKs) from the Silencer® Select human kinase siRNA library V4 on the viability of A925L cells was assessed using MTT assays. The 56 RTKs are rank-ordered from highest to lowest according to their inhibitory effect on the viability of A925L cells relative to nonspecific control siRNA. The effects of the top 10 genes indicated by a red line are further shown in **d**. **e** A925L and H2228 cells treated with nonspecific control siRNA or EGFR-specific siRNAs were incubated with or without alectinib (100 nmol/L), brigatinib (100 nmol/L), or lorlatinib (100 nmol/L) for 72 h and cell viability was detected using MTT assays. *P < 0.05 (one-way ANOVA). **f** Western blotting of A925L and H2228 cells treated with nonspecific control siRNA or EGFR-specific siRNA and incubated with or without lorlatinib (100 nmol/L) for 4 h. Data are represented as mean ± S.D.

ALK-rearranged NSCLC cells, including A925L and H2228 cells, were highly sensitive to lorlatinib (Supplementary Fig. 1). However, some of the A925L and H2228 cells survived, even after long exposure to high concentration (1 µmol/L) of lorlatinib for 9 days (Fig. 1a). To investigate the molecular mechanisms via which these cells escaped the effects of lorlatinib, we generated drug-tolerant (DT) cells from A925L and H2228 cells exposed to high doses of lorlatinib (1 or 10 µmol/L, respectively) for 9 days (Supplementary Fig. 2a). Compared to their parental cells, these DT cells were resistant to lorlatinib (Supplementary Fig. 2b) and a large population of cells accumulated in the G1 phase, as reported previously^18,19^ (Supplementary Fig. 2c). We evaluated 49 phospho-receptor tyrosine kinases (RTKs) in parental and DT cells using a phospho-kinase antibody array in A925L and H2228 cells, and EGFR phosphorylation was higher in DT cells than in their parental cells (Fig. 1b, Supplementary Fig. 3). Among the upregulated RTKs in DT cells of A925L and H2228, EGFR knockdown resulted in the largest decrease in the viability of both DT cells in the presence of lorlatinib (Supplementary Fig. 4). To identify pathways associated with DT cell characteristics, we performed transcriptome analysis between parental and DT cells using a gene expression microarray in A925L and H2228 cells; 1385 and 500 upregulated genes (*q* < 0.05, log2 Fold Change > 1.0) in A925L and H2228 cells, respectively, were identified (Supplementary Fig. 5a, Supplementary Table 1). Pathway analysis using the Kyoto Encyclopedia of Genes and Genomes (KEGG) database on 253 genes with upregulated expression in both A925L and H2228 showed that the ERBB signaling pathway was enriched (Supplementary Fig. 5b). To further elucidate the molecular mechanism of adaptive resistance to lorlatinib from a different perspective, a siRNA library was used to screen for factors related to adaptive resistance to lorlatinib treatment. Among 56 receptor-type tyrosine kinase siRNAs, we focused on knocking down *EGFR* because of its remarkable synergistic effect in reducing the viability of A925L cells (Fig. 1c, d). We confirmed that *EGFR* knockdown enhanced the efficacy of multiple ALK-TKIs, namely, alectinib, brigatinib, and lorlatinib, in terms of A925L and H2228 cell viability (Fig. 1e). Western blotting showed that compared to lorlatinib monotherapy, the combination of *EGFR* knockdown and lorlatinib suppressed phosphorylation of AKT and ERK, which are downstream survival signals of ALK (Fig. 1f). These results showed that EGFR plays a pivotal role in determining the sensitivity of lorlatinib in ALK-rearranged lung cancer cells.

### Adaptive feedback loop of lorlatinib is induced by HB-EGF-mediated EGFR activation

**Fig. 2:**
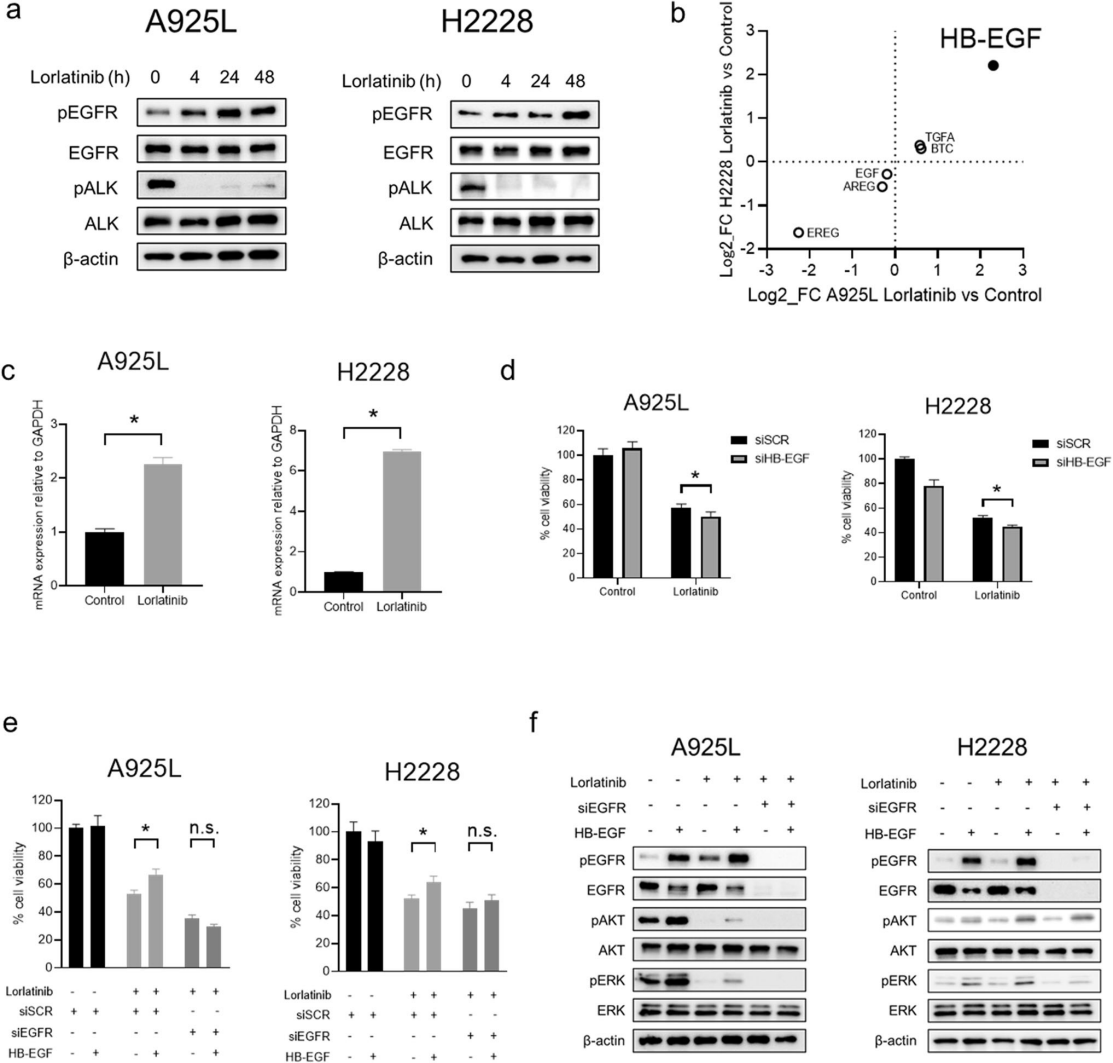
EGFR activation by lorlatinib treatment is induced by exogenous overexpression of HB-EGF. **a** Western blotting of A925L and H2228 cells treated with lorlatinib (100 nmol/L) for indicated durations. **b** Scatterplot of EGFR ligand expression analyzed using microarrays in either A925L or H2228 DT cells vs. parent cells. **c** qPCR of heparin-binding EGF-like growth factor (HB-EGF) in A925L and H2228 parent cells and cells treated with lorlatinib (100 nmol/L) for 48 h. *P < 0.01 (unpaired *t-*tests). **d** A925L and H2228 cells with nonspecific control siRNA or HB-EGF-specific siRNAs were treated for 72 h with or without lorlatinib (100 nmol/L). Cell growth was determined using MTT assays. *P < 0.05 (two-way ANOVA). **e** A925L and H2228 cells with nonspecific control siRNA or EGFR-specific siRNAs were treated for 72 h with or without lorlatinib (100 nmol/L) and/or HB-EGF (50 ng/mL, 10 ng/mL, respectively). Cell growth was determined using MTT assays. *P < 0.01 (two-way ANOVA). **f** Western blotting of A925L and H2228 cells treated with nonspecific control siRNA or EGFR-specific siRNA and incubated with or without lorlatinib (100 nmol/L) and/or HB-EGF (50 ng/mL, 10 ng/mL, respectively) for 4 h. Data are represented as mean ± S.D.

We next examined the fundamental roles of EGFR signaling on the sensitivity of ALK-rearranged NSCLC cells treated with lorlatinib. We hypothesized that tolerance to lorlatinib may be caused by a survival signal via EGFR. Western blotting showed that while EGFR was not remarkably phosphorylated, its phosphorylation was induced by lorlatinib at 4 h and that it increased through 48 h in A925L and H2228 cells. (Fig. 2a). To further investigate the mechanism underlying EGFR activation, we evaluated the expression of EGFR ligands between parental and DT A925L and H2228 cells using transcriptome analysis. Among several expressed EGFR ligands, HB-EGF was more upregulated in DT cells than in parental cells (Fig. 2b). Evaluation of HB-EGF expression using real-time PCR showed that treatment with lorlatinib enhanced mRNA level of HB-EGF in A925L and H2228 cells (Fig. 2c). The production of endogenous HB-EGF proteins in cell culture supernatants tended to increase when treated with lorlatinib in A925L cells (Supplementary Fig. 6). Knockdown of HB-EGF by specific siRNA enhanced the efficacy of lorlatinib on inhibition of the viability of A925L and H2228 cells (Fig. 2d). Recombinant HB-EGF reduced the sensitivity of ALK-rearranged lung cancer cells to lorlatinib, whereas *EGFR* knockdown restored the sensitivity of A925L and H2228 cells to lorlatinib in the presence of HB-EGF (Fig. 2e). Western blotting showed that HB-EGF stimulation induced phosphorylation of EGFR, AKT, and ERK when treated with lorlatinib, whereas *EGFR* knockdown restored these responses (Fig. 2f). These results revealed that HB-EGF-mediated EGFR activation after lorlatinib intervention promoted the adaptive resistance to lorlatinib in ALK-rearranged lung cancer cells.

### Activation of the c-Jun pathway by lorlatinib accelerated EGFR signaling as adaptive resistance

**Fig. 3:**
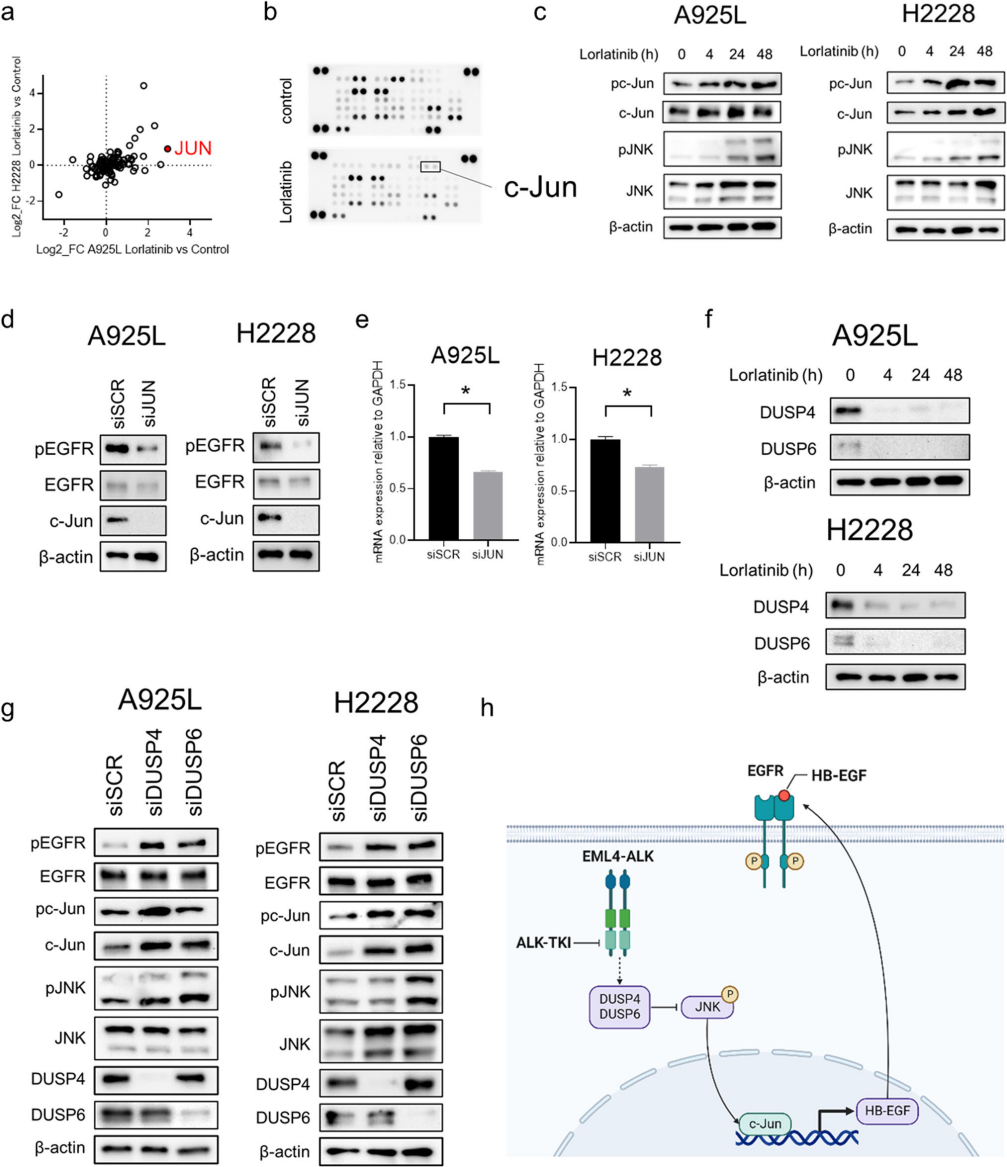
EGFR activation is induced by JNK/c-Jun axis activation via suppression of DUSP4 and DUSP6. **a** Scatterplot of genes in the KEGG_ERBB_SIGNALING_PATHWAY gene set in either A925L or H2228 DT cells vs. that in parent cells. **b** Human phospho-kinase array analysis of parental A925L and H2228 cells, as well as cells treated with lorlatinib (100 nmol/L) for 48 h. **c** Western blotting of A925L and H2228 cells treated with lorlatinib (100 nmol/L) for the indicated durations. **d** A925L and H2228 cells were incubated with nonspecific control siRNA or *JUN*-specific siRNA and lysed, and the indicated proteins were detected using western blotting. **e** qPCR of heparin-binding EGF-like growth factor (HB-EGF) in parent A925L and H2228 cells and cells incubated with nonspecific control siRNA or *JUN*-specific siRNA. *P < 0.01 (unpaired *t*-tests). **f** Western blotting of A925L and H2228 cells treated with lorlatinib (100 nmol/L) for the indicated durations. **g** A925L and H2228 cells were incubated with nonspecific control siRNA and DUSP4 or DUSP6-specific siRNA and lysed, and the indicated proteins were detected using western blotting. **h** Schematic diagram showing the mechanisms of adaptive resistance, including activation of EGFR signaling via endogenous HB-EGF stimulation, the feedback loop of which was induced by the JNK/c-Jun axis, in ALK-rearranged NSCLC cells. Data are represented as mean ± S.D.

To elucidate the molecular mechanisms underlying EGFR activation by lorlatinib, we investigated the changes in gene expression between ALK-rearranged lung cancer cells and their DT cells using microarray analysis. Among the several expressed genes associated with the ERBB pathway, JUN, a transcription factor, showed remarkable upregulation in DT cells compared to that in parental cells (Fig. 3a, Supplementary Fig. 7). In addition, the results obtained using the phospho-kinase antibody array showed that phosphorylation of c-Jun was activated by lorlatinib exposure in A925L cells (Fig. 3b). Based on these results, we hypothesized that c-Jun might play a pivotal role in the activation of EGFR by lorlatinib. Western blotting showed that lorlatinib increased the levels of phosphorylated and total c-Jun and JNK in both A925L and H2228 cells (Fig. 3c). Knockdown of *JUN* decreased the expression of phosphorylated-EGFR and enhanced the efficacy of lorlatinib in promoting the survival in A925L and H2228 cells (Fig. 3d, Supplementary Fig. 8). In addition, the results of real-time PCR showed that *JUN* knockdown reduced the level of HB-EGF mRNA (Fig. 3e). These results indicated that the HB-EGF/EGFR axis is activated via the c-Jun pathway for ALK-rearranged NSCLC cells treated with lorlatinib. To further elucidate the molecular mechanisms underlying tolerance to lorlatinib, we next investigated the expression of the DUSP family proteins, which are known as JNK phosphatases. Lorlatinib considerably inhibited DUSP4 and DUSP6 expression at 4 h, which continued up to 48 h in A925L and H2228 cells (Fig. 3f). In addition, DUSP4 or DUSP6 knockdown induced the activation of the JNK/c-Jun axis and EGFR in H2228 and A925L cells (Fig. 3g). Moreover, DUSP4 and DUSP6 expression was lower in DT cells than in parental cells (Supplementary Fig. 9). These observations suggested that the DUSP family proteins might be involved in adaptive resistance to lorlatinib via c-JUN signaling (Fig. 3h).

### EGFR inhibitor sensitized ALK-rearranged lung cancer cells expressing high levels of EGFR to lorlatinib

**Fig. 4:**
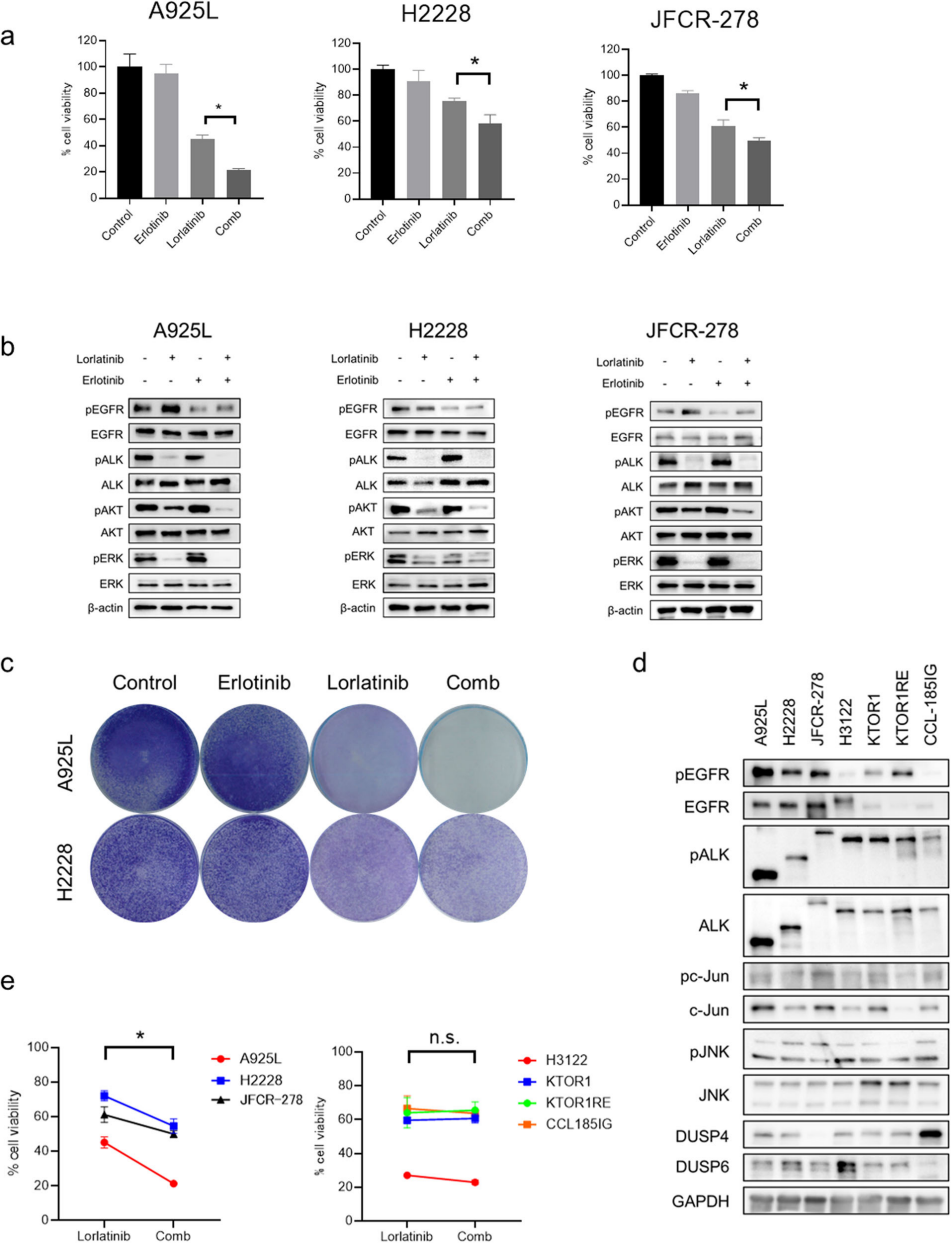
EGFR inhibitor sensitized ALK-rearranged lung cancer cells expressing high levels of EGFR to lorlatinib. **a** A925L, H2228, and JFCR-278 cells were incubated with lorlatinib (100 nmol/L), erlotinib (100 nmol/L), or a combination of lorlatinib and erlotinib for 72 h. Cell growth was determined using MTT assays. *P < 0.05 (one-way ANOVA). **b** Western blotting of A925L, H2228, and JFCR-278 cells treated with lorlatinib (100 nmol/L), erlotinib (100 nmol/L), or a combination of lorlatinib and erlotinib for 4 h. **c** A925L and H2228 cells were visualized using crystal violet staining following 9 days of treatment with lorlatinib (100 nmol/L), erlotinib (100 nmol/L), or combination of lorlatinib and erlotinib. **d** Western blotting of ALK-rearranged NSCLC parental cells. **e** Quantitative determination of the inhibition of cell viability of high- and low-EGFR-expressing ALK-rearranged NSCLC cells treated with lorlatinib in the presence or absence of erlotinib. Cell growth was determined using MTT assays. *P < 0.05 (paired Student’s *t*-test). Data are represented as mean ± S.D.

We investigated whether an EGFR inhibitor could augment the sensitivity of ALK-rearranged NSCLC cells to ALK-TKIs. Erlotinib, a first-generation EGFR inhibitor, did not affect the viability of the ALK-rearranged NSCLC cell lines that were tested. The use of a combination of erlotinib and lorlatinib for 72 h increased the sensitivity of the cells to lorlatinib and reduced the viability of A925L, H2228, and JFCR-278 cells (Fig. 4a). Western blotting showed that treatment of A925L, H2228, and JFCR-278 cells with lorlatinib and erlotinib combination for 4 h inhibited the phosphorylation of AKT and ERK more than treatment with lorlatinib alone (Fig. 4b). In addition, the continuous co-treatment of A925L and H2228 cells with erlotinib and lorlatinib for 9 days reduced cell viability compared to that observed with lorlatinib alone (Fig. 4c). Recombinant HB-EGF stimulation induced resistance of A925L and H2228 cells to lorlatinib, whereas the combination of erlotinib and lorlatinib circumvented this (Supplementary Fig. 10a). HB-EGF stimulation increased phosphorylation of AKT and/or ERK in A925L and H2228 cells treated with lorlatinib, whereas the erlotinib-lorlatinib combination reduced the phosphorylation of these proteins (Supplementary Fig. 10b).

Next, we evaluated the correlation between total EGFR expression and insensitivity to lorlatinib in seven ALK-rearranged NSCLC cell lines to subsequently assess the additional efficacy of erlotinib. The seven ALK-rearranged NSCLC cell lines were divided into two groups based on high and low levels of EGFR expression at baseline (Fig. 4d). Erlotinib accelerated the sensitivity to several ALK-TKIs, including lorlatinib, and reduced the viability of high EGFR-expressing cells (A925L, H2228, and JFCR-278) but only showed marginal effects on the viability of low-EGFR-expressing cells (H3122, KTOR1, KTOR1 RE, and CCL185IG) (Fig. 4e, Supplementary Fig. 11a, b). The combination index values after co-treatment with lorlatinib and erlotinib were less than 1.0, indicating a synergic effect in A925L and H2228 cells (Supplementary Fig. 11c). Western blotting showed that treatment of low EGFR-expressing cells with the combination of lorlatinib and erlotinib for 4 h did not enhance the suppression of phosphorylation of AKT and ERK compared with their treatment with only lorlatinib (Supplementary Fig. 11d). These findings suggested that high EGFR expression at the baseline might be a predictive biomarker for responders to combination therapy with lorlatinib and erlotinib among ALK-rearranged NSCLC cells.

### EGFR inhibitor promoted apoptosis of ALK-rearranged lung cancer cells after lorlatinib treatment via down-regulation of Bcl-xL

**Fig. 5:**
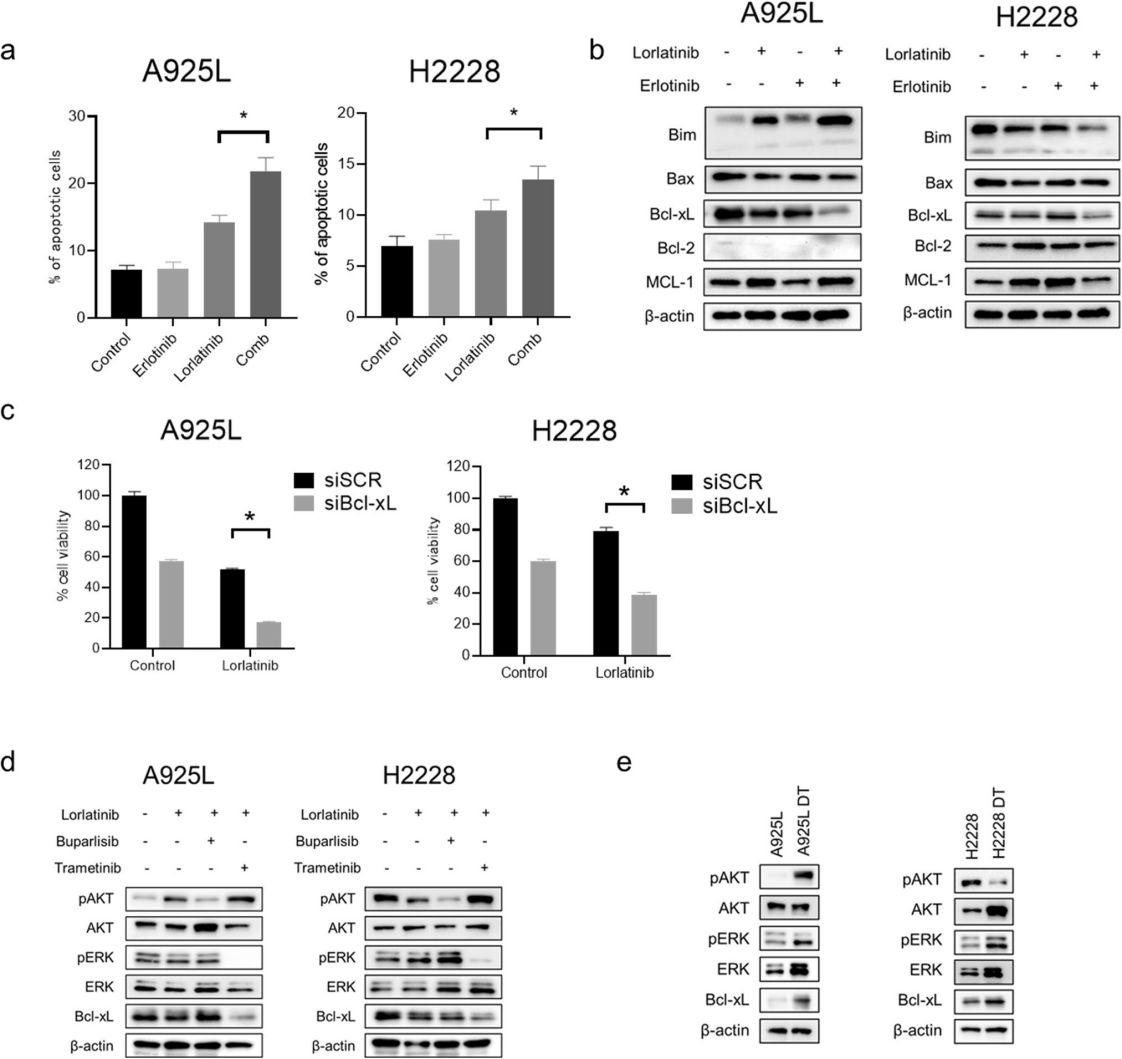
Combination therapy with lorlatinib and erlotinib inhibits cell proliferation by inducing apoptosis via suppression of Bcl-xL. **a** Apoptotic cell percentages of A925L and H2228 cells, which were double-stained with annexin V and propidium iodide, were detected using flow cytometry following treatment with lorlatinib (100 nmol/L), erlotinib (100 nmol/L), or a combination of lorlatinib and erlotinib for 48 h. *P < 0.05 (one-way ANOVA). **b** A925L and H2228 cells were incubated with lorlatinib (100 nmol/L), erlotinib (100 nmol/L), or a combination of lorlatinib and erlotinib for 96 h, and the indicated proteins were detected using western blotting. **c** A925L and H2228 cells treated with nonspecific control siRNA or Bcl-xL-specific siRNAs were incubated with or without lorlatinib (100 nmol/L) for 72 h. Cell growth was determined using MTT assays. *P < 0.01 (two-way ANOVA). **d** A925L and H2228 cells were incubated with lorlatinib (100 nmol/L), a combination of lorlatinib and buparlisib (100 nmol/L), or a combination of lorlatinib and trametinib (100 nM) for 96 h, and the indicated proteins were detected using western blotting. **e** Western blotting of parental cells and DT cells generated from A925L and H2228 cells. Data are represented as mean ± S.D.

To further assess the additional efficacy of erlotinib, we investigated the status of cell cycle and apoptosis in A925L and H2228 cells. Combination of lorlatinib with erlotinib increased G1 arrest, compared to that observed after lorlatinib monotherapy in A925L cells, but not in H2228 cells (Supplementary Fig. 12). In contrast, the number of apoptotic cells increased after lorlatinib monotherapy, and more strongly after treatment with the erlotinib combination in both A925L and H2228 cells (Fig. 5a). Next, we examined the expression levels of apoptosis-related proteins to investigate the mechanisms underlying the apoptotic process induced by the combination therapy with erlotinib. Western blot analysis showed that compared to that observed with lorlatinib monotherapy, the combination therapy enhanced the reduction in the level of the anti-apoptotic factor Bcl-xL (Fig. 5b). Specific siRNA-mediated knockdown of Bcl-xL in A925L and H2228 cells significantly reduced cell viability after treatment with lorlatinib (Fig. 5c). To investigate the effect of downstream molecules on the adaptive resistance to lorlatinib, we evaluated the expression of Bcl-xL when lorlatinib was used in combination with the MEK inhibitor trametinib or the PI3K inhibitor buparlisib. Combination therapy with trametinib and lorlatinib downregulated Bcl-xL via inhibition of the MEK/ERK pathway, compared with monotherapy with lorlatinib (Fig. 5d). In addition, the use of trametinib inhibited cell viability to a greater degree than buparlisib in ALK-rearranged NSCLC cells treated with lorlatinib (Supplementary Fig. 13). Bcl-xL and phosphorylation of ERK were upregulated in A925L and H2228 DT cells compared to their levels in parental cells (Fig. 5e). These results suggest that the upregulation of Bcl-xL via ERK reactivation is involved in the adaptive resistance of ALK-rearranged lung cancer cells, and that inhibition of the MEK/ERK pathway by combination therapy with lorlatinib and erlotinib induces apoptosis via suppression of Bcl-xL.

### ALK-rearranged lung cancer cells with acquired resistance were less dependent on EGFR activity

To examine the roles of EGFR activation in acquired resistance to lorlatinib, we established A925L LR cells with acquired resistance to lorlatinib using in vitro step-wise methods (Supplementary Fig. 14a). The A925L LR cells were moderately resistant to lorlatinib (Supplementary Fig. 14b). In addition, the levels of phosphorylated and total EGFR protein and EGFR mRNA were higher in the A925L LR cells than in their parental cells (Supplementary Fig. 14c, d). Specific siRNAs for ALK marginally affected the viability of A925L LR cells, showing independence from ALK signaling in A925L LR cells (Supplementary Fig. 14e, f). Cell line-based analysis showed that the A925L LR cells were less sensitive to the combination of lorlatinib and erlotinib than A925L parental cells, despite higher EGFR expression in A925L LR cells (Supplementary Fig. 14g). Compared to that in A925L cells, the phosphorylation of AKT and ERK was mildly inhibited in A925L LR cells treated with lorlatinib monotherapy or the erlotinib combination after 4 h (Supplementary Fig. 14h). These results indicated that the survival of cells with acquired resistance to lorlatinib had a marginal dependency on EGFR signaling and that EGFR-targeted combination treatment might be more effective in the initial phase than at the lorlatinib-acquired resistance phase in ALK-rearranged NSCLC cells.

### Initial co-treatment with erlotinib and lorlatinib prevented cell-line-derived xenograft (CDX) tumor regrowth

**Fig. 6:**
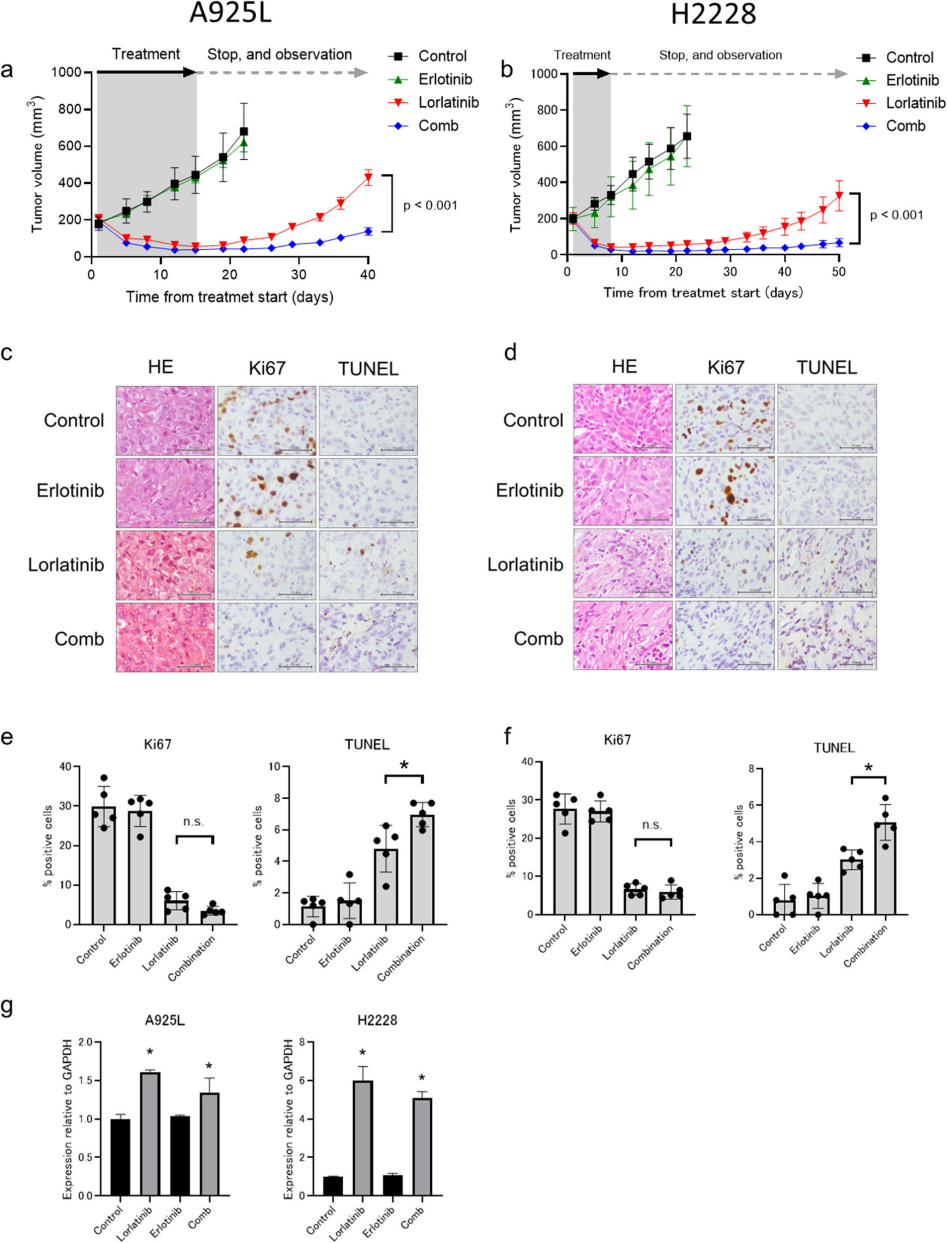
Combination with EGFR inhibitor and ALK-TKIs prevented the emergenceof ALK-rearranged NSCLC in vivo. **a** A925L xenografts were treated with a vehicle (control), lorlatinib (5 mg/kg), erlotinib (25 mg/kg), or lorlatinib plus erlotinib (*n* = 6) for 15 days daily via oral gavage. Thereafter, treatment was discontinued, and tumor regrowth was evaluated on day 40. **b** H2228 xenografts were treated daily with a vehicle (control), lorlatinib (1.5 mg/kg), erlotinib (25 mg/kg), or lorlatinib plus erlotinib (*n* = 6) for 7 days via oral gavage. Thereafter, the treatment was discontinued, and tumor regrowth was evaluated on day 40. Tumor volume was measured from the start of treatment and is shown as mean ± SEM. *P < 0.01 (one-way ANOVA). Representative immunohistochemical staining images of A925L (**c**) and H2228 (**d**) xenografts with antibodies to human Ki-67 and TUNEL assays. Bar, 50 μm. A925L cell line-derived xenograft (CDX) tumors were treated daily with a vehicle (control), lorlatinib (5 mg/kg), erlotinib (25 mg/kg), or lorlatinib plus erlotinib (*n* = 6) for four days via oral gavage. H2228 CDX tumors were treated daily with a vehicle (control), lorlatinib (1.5 mg/kg), erlotinib (25 mg/kg), or lorlatinib plus erlotinib (*n* = 6) for four days via oral gavage. Proliferative and apoptotic cells were quantified by their Ki-67-positive proliferation index (percentage of Ki-67-positive cells) and TUNEL assays in A925L (**e**) and H2228 (**f**) cells. *P < 0.05 (one-way ANOVA). Data are represented as mean ± S.D. **g** qPCR of heparin-binding EGF-like growth factor (HB-EGF) in A925L and H2228 CDX tumors treated with a vehicle (control), lorlatinib, erlotinib, or a combination of lorlatinib and erlotinib for 4 days. *P < 0.05 (one-way ANOVA). Data are represented as mean ± S.D.

We next evaluated the effect of the initial combination of erlotinib plus lorlatinib in CDX models established using A925L and H2228 cells. Treatment with erlotinib monotherapy showed marginal antitumor effects on the growth of subcutaneous tumors of A925L and H2228 cells, whereas treatment with lorlatinib monotherapy caused tumor regression within 1 week; however, small tumors that remained during lorlatinib treatment re-grew following the discontinuation of lorlatinib after 15 days of treatment in the CDX model with A925L (rapid recurrence), and after 20 days in the CDX model with H2228 cells (slow recurrence). Combined treatment with lorlatinib and erlotinib caused tumor regression within 1 week; however, the size of the regressed tumors continued to shrink after more than 2–3 weeks of treatment discontinuation in both models (Fig. 6a, b).

Following a 4-day treatment, the number of Ki-67-positive proliferating tumor cells did not differ significantly between the lorlatinib- or combination-treated tumors derived from A925L and H2228 cells, respectively (Fig. 6c, d). However, consistent with the results of the cell line-based analysis, a significant increase in the number of TUNEL-positive apoptotic tumor cells was observed in the combination drug-treated tumors than in tumors treated with lorlatinib monotherapy in both A925L and H2228 cells (Fig. 5e, f). Furthermore, real-time PCR analysis showed that treatment with lorlatinib enhanced mRNA levels of HB-EGF in xenograft tumors from A925L and H2228 cells, regardless of erlotinib addition (Fig. 6g). Immunohistochemistry assays showed that Bcl-xL expression was remarkably reduced in tumors of the combination group (Supplementary Fig. 15). No apparent adverse events, including weight loss, were observed during these treatments (Supplementary Fig. 16a, b). These observations with the CDX models showed that initial EGFR inhibition significantly delayed the regrowth of ALK-rearranged NSCLC tumors with both rapid and slow recurrences after lorlatinib treatment by accelerating cell apoptosis.

### Association pre-treatment, total- and phosphorylated-EGFR expression, and alectinib outcomes in patients with ALK-rearranged NSCLC

We retrospectively investigated the correlation between the expressions of total- and phosphorylated-EGFR and the clinical efficacy of ALK-TKI alectinib in 26 treatment-naïve patients with ALK-rearranged NSCLC. The probability of total- and phospho-EGFR expressions in pre-alectinib-treated tumor cells was evaluated using immunohistochemical staining (Supplementary Fig. 17a, b). Out of the 26 tumor specimens, 16 (61.5%) expressed low total-EGFR and 10 (38.5%) expressed high total-EGFR. Further, 15 (57.7%) expressed low phospho-EGFR and 11 (42.3%) expressed high phospho-EGFR (Supplementary Table 2). Following alectinib treatment, patients with high total-EGFR expression tumors had a significantly shorter progression-free survival (PFS) than those with low total-EGFR expression tumors (median PFS = 26 and 60 months, respectively; *p* = 0.01; Supplementary Fig. 17c) and tended to exhibit shorter overall survival (OS) following alectinib treatment (median OS = 32 months vs. not reached; *p* = 0.09; Supplementary Fig. 17d). Patients with high phospho-EGFR expression tumors tended to exhibit shorter PFS (median PFS = 26 and 50 months; *p* = 0.36; Supplementary Fig. 17e) and OS (median OS = 46 months vs. not reached; *p* = 0.19; Supplementary Fig. 17f), compared to those with low phospho-EGFR expression tumors. These observations suggest that high pre-treatment EGFR expressions may be correlated with a poor response to ALK-TKI in patients with ALK-rearranged NSCLC.

## Discussion

Despite their remarkable performances, the effects of molecule-targeted single agents are transient because of the presence of residual disease. In addition, the fate of the surviving tumor cells after intervention of molecule-targeted agents remains unknown. In this study, we revealed a novel mechanism of adaptive resistance to the third generation ALK-TKI, lorlatinib, in ALK-rearranged lung cancer. To the best of our knowledge, this is the first study to demonstrate the mechanism underlying the adaptive resistance to lorlatinib, which involves the HB-EGF/EGFR axis of the ALK-rearranged NSCLC cell line, and to identify the efficacy of combination therapy for EGFR and ALK aimed at tumor eradication in ALK-rearranged lung cancer.

Our observations of clinical specimens demonstrated that total- and phosphorylated-EGFR expression is related to poor outcomes in naïve ALK-rearranged NSCLC patients, indicating that a novel therapeutic strategy is required for these patients. When exposed to ALK-TKIs, a small fraction of cells survives and expands, leading to acquired drug resistance and tumor heterogeneity, eventually promoting tumor recurrence^20,21^. Mutation profiling of circulating tumor cells in ALK-rearranged NSCLC showed progressive intratumor heterogeneity and concomitant co-occurrence of mutations in ALK-independent pathways in acquired resistance to ALK-TKI^22,23^; thus, progression of tumor heterogeneity makes conquering drug resistance in ALK-rearranged NSCLC tumors difficult. Therefore, overcoming adaptive resistance using early therapeutic intervention might improve the clinical outcomes of patients with ALK-rearranged NSCLC.

In this study, cell-based assays showed that lorlatinib, in combination with EGFR blockade initiated during the initial phase, exerted significant inhibitory effects on ALK-rearranged NSCLC cells. Moreover, the CDX models demonstrated that the combination of erlotinib and lorlatinib prevented the regrowth of ALK-rearranged NSCLC tumors with both rapid and slow recurrences after treatment discontinuation. Therefore, EGFR activation retains the viability of ALK-TKI-treated cells in vivo, while EGFR inhibition improves the response of ALK-rearranged NSCLC tumors to lorlatinib treatment by preventing recurrence. We also examined the role of EGFR signaling in both intrinsic and acquired resistance to lorlatinib. Reports have shown that EGFR signal activation induced acquired resistances to ALK-TKIs, including lorlatinib, for ALK-rearranged NSCLC^24–27^. However, our observations showed that the additional effect of EGFR inhibition on cell viability was limited in the lorlatinib-acquired resistance phase compared to that in the initial phase of lorlatinib resistance, indicative of progressive intratumor heterogeneity in the acquired resistance phase. These results suggested that treatment with a combination of lorlatinib and erlotinib might be more effective at intervention in the initial phase than at the lorlatinib-acquired resistance phase during the growth of ALK-rearranged NSCLC cells. The first-generation EGFR-TKI, erlotinib, has been approved as the first-line of treatment for patients with advanced EGFR-mutant NSCLC and is clinically used as monotherapy or combined therapy with tolerability and safety. In this study, we indicated the potential of erlotinib for drug repositioning in ALK-rearranged NSCLC patients treated with lorlatinib. This combination has to be further evaluated in a clinical study.

We also revealed the fundamental feedback mechanisms underlying EGFR-mediated adaptive reaction in ALK-rearranged NSCLC treated with lorlatinib. Several studies, including our previous study, have reported that stimulation with the exogenous EGFR ligand induces intrinsic resistance to ALK-TKIs in ALK-rearranged NSCLC^28,29^. In addition, HB-EGF which is one of the EGFR ligands, has been reported to be a promising therapeutic target for ovarian, breast, gastric, and endometrial cancers^27^. In this study, the production of endogenous HB-EGF was involved in drug tolerance to lorlatinib via EGFR activation in ALK-rearranged NSCLC cells.

c-Jun is known as a component of the transcription factor activator protein-1 (AP-1), phosphorylation of which is regulated by JNK, a serine-threonine protein kinase^30^. Reports have shown that JNK/c-Jun signaling induces activation of EGFR signaling via HB-EGF overexpression in various types of cancer, promoting tumor invasion and drug resistance^31–33^. The DUSP family proteins, including DUSP4 and DUSP6, negatively regulate the JNK signaling pathway via dephosphorylation^34^. Previously, we have reported that in ALK-rearranged NSCLC, ALK inhibition activates the JNK/c-Jun pathway via DUSP4^17^. In addition, ERBB signaling was activated by DUSP4 suppression in melanoma cells treated with trametinib^35^. These data indicated that DUSP family proteins, as feedback loops of lorlatinib, play important roles in the establishment of adaptive resistance via JNK/c-Jun activation in ALK-rearranged NSCLC, leading to the activation of HB-EGF/EGFR signaling.

Bcl-xL is involved in the drug resistance of numerous types of tumors, including NSCLC, breast cancer, and ovarian cancer^36^. Previous studies have shown that DT factors, YAP1 and STAT3, promoted apoptosis of ALK-rearranged NSCLC cells via regulation of Bcl-xL^14,15^. In this study, Bcl-xL expression was mainly modulated by the ERK axis at the phase of adaptive response to lorlatinib. Moreover, the increase of Bcl-xL expression was sustained by ERK reactivation during the tolerant phase, whereas Bcl-xL expression decreased due to the inhibition of ERK signaling by combination therapy with lorlatinib and erlotinib. These observations suggest that the ALK-TKI-tolerant mechanisms might involve avoidance of apoptosis via ERK-mediated regulation of Bcl-xL.

In this study, among the several mechanisms of adaptive resistance in ALK-rearranged lung cancer cells, we focused on EGFR signaling. In contrast, transcriptome analysis and siRNA library showed that diverse epigenetic mechanisms intricately affect the adaptive resistance in ALK-rearranged lung cancer, indicating that EGFR is involved in part of the adaptive response to lorlatinib. Therefore, further investigations are needed to understand the fully adaptive resistances in ALK-rearranged lung cancer.

In summary, the mechanisms underlying lorlatinib-induced adaptive resistance in ALK-rearranged NSCLC rely on activation of EGFR signaling via endogenous HB-EGF stimulation, the feedback loop of which is induced by the JNK/c-Jun axis. Importantly, the combination of lorlatinib and erlotinib effectively suppressed the adaptive survival of ALK-rearranged lung cancer cells by enhancing apoptosis induction via suppression of Bcl-xL. These results indicated that the combination of lorlatinib and erlotinib has the potential to improve outcomes in ALK-rearranged lung cancer. The safety and efficacy of this combination therapy will be validated in a future clinical setting for untreated ALK-rearranged lung cancer.

## Methods

### Cell culture and reagents

A925L (EML4–ALK variant 5a, E2: A20) was established from a surgical specimen of the EML4–ALK-positive NSCLC patient kindly provided by Fumihiro Tanaka of the Second Department of Surgery, University of Occupational and Environmental Health, Japan^37^. H2228 (EML4–ALK variant 3a/b E6; A20) and CCL-185IG™ (A549 EML4-ALK + ) cells were purchased from the American Type Culture Collection (Manassas, VA, USA). The H3122 human lung adenocarcinoma cell line, with EML4–ALK fusion protein variant 1 (E13; A20), was kindly provided by Dr. Jeffrey A. Engelman of the Massachusetts General Hospital Cancer Center (Boston, MA, USA)^38^. The KTOR1 and KTOR1-RE cells with EML4–ALK fusion protein variant 1 were established from patients with ALK-rearranged NSCLC who regularly visited the Kyoto University Hospital^14^. These cell lines were maintained in RPMI 1640 medium with 10% fetal bovine serum (FBS), penicillin (100 U/mL), and streptomycin (100 µg/mL) in a humidified 5% CO_2_ incubator at 37 °C. Alectinib-resistant ALK fusion-positive NSCLC PDC line JFCR-278 was cultured in RPMI-1640 medium and Ham’s F-12 medium with 20 mM 4-(2-hydroxyethyl)-1-piperazineethanesulfonic acid (HEPES; Nacalai Tesque Inc., Kyoto, Japan), 15% FBS, and 1× antibiotic–antimycotic mixed stock solution (Nacalai Tesque). All cells were passaged for less than three months before being renewed with frozen early-passage stocks and were regularly screened for *Mycoplasma* using a MycoAlert *Mycoplasma* detection kit (Lonza, Basel, Switzerland). Lorlatinib, alectinib, brigatinib and erlotinib were obtained from Selleck Chemicals (Houston, TX, USA).

### Cell viability assay

Tumor cells (2–3 × 10^3^ cells/100 μL/well) were cultured with the indicated compounds for 72 h, after which cell viability was determined using MTT assays (Sigma-Aldrich, St. Louis, MO, USA) according to the manufacturer’s instructions. Absorbance was measured using a microplate reader at the test and reference wavelengths of 570 nm and 630 nm, respectively. Percentage growth was determined relative to untreated controls. Cells were also treated at 37 °C with the indicated agents for two weeks, and the drugs were replenished every 72 h.

### Microarray gene expression study

Similar to previous studies^18^, total RNA was extracted using a NucleoSpin® RNA Plus kit (Takara Bio, Shiga, Japan) according to the manufacturer’ s instructions. cDNA was synthesized using GeneChip® WT PLUS reagent kit (Thermo Fisher Scientific, Waltham, MA, USA) and hybridized to the array in Clariom^TM^ S human assay (Affymetrix, Santa Clara, CA, USA). The arrays were scanned and normalized with the Signal Space Transformation-Robust Multi-array Average algorithm using the Affymetrix GeneChip Command Console (Thermo Fisher Scientific). Differential gene expression analysis was performed using Transcriptome Analysis Console Software 4.0.2.15 (Thermo Fisher Scientific).

### Human phospho-RTK array

Similar to previous studies^18^, the relative phosphorylation levels of 49 RTKs and two related proteins were measured using a human phospho-RTK array kit (R & D Systems, Minneapolis, MN, USA) according to the manufacturer’s instructions, with slight modifications. Briefly, cells were cultured in RPMI 1640 medium containing 10% FBS and lysed in array buffer prior to reaching confluence. The arrays were blocked with blocking buffer and incubated overnight with 300 μg cell lysate at 4 °C before being washed, incubated with horseradish peroxidase (HRP)-conjugated phospho-kinase antibodies, and treated with SuperSignal West Dura Extended Duration substrate (Pierce Biotechnology, Rockford, IL, USA).

### siRNA transfection

Duplexed Silencer® Select siRNAs for *EGFR* (s565)*, HER2* (s613)*, HER3* (s4780)*, IGF1R* (s7211)*, FGFR1* (s5165)*, InsulinR* (s533802)*, AXL* (s1846)*, HBEGF* (s4353)*, JUN* (s7658)*, DUSP4* (s1922)*, DUSP6* (s4379), and *Bcl-xL* (s4373) were purchased from Invitrogen (Carlsbad, CA, USA). The ALK-targeted siRNAs were purchased from Santa Cruz Biotechnology (Dallas, TX, USA). Cells were transfected with these siRNAs using Lipofectamine RNAi-MAX (Invitrogen) according to the manufacturer’s instructions. In all experiments, Silencer® Select siRNA for negative control #1 (Invitrogen) was used as the scrambled control. *EGFR, JUN, DUSP4, DUSP6*, and *ALK* knockdown were confirmed using western blot analysis. To perform a comprehensive analysis of siRNA screening, the Silencer® Select human kinase siRNA library V4 (Ambion, 4397918) was used according to the manufacturer’s instructions.

### Measurement of HB-EGF using enzyme-linked immunosorbent assay (ELISA)

Tumor cell culture supernatants were measured for heparin-binding EGF (HB-EGF) using ELISA kits according to the manufacturer’s instructions; detection limit was 0.787 pg/mL.

### Human phospho-kinase array

The relative phosphorylation of 37 kinase phosphorylation sites and two related total proteins were measured using a Proteome Profiler human phospho-kinase array kit (R & D Systems) according to the manufacturer’s instructions, with slight modifications. The incubated cells were lysed in array buffer and 600 μg of cell lysate was used for the assays.

### Western blotting

Similar to previous studies^18^, proteins (25-μg aliquots) were resolved using SDS polyacrylamide gel electrophoresis (Bio-Rad, Hercules, CA, USA). The electrophoresed protein samples were transferred to polyvinylidene difluoride membranes (Bio-Rad), washed thrice with TBS, and incubated with a blotting-grade blocker (Bio-Rad) for 1 h at 25 °C before incubating overnight at 4 °C with primary antibodies. The antibodies used in this study are shown in Supplementary Table 3. The membranes were then washed thrice with TBS and incubated with HRP-conjugated species-specific secondary antibodies for 1 h at 25 °C, and the immunoreactive bands were visualized using SuperSignal West Dura Extended Duration substrate (Pierce Biotechnology, Waltham, MA, USA). All of the uncropped western blots with molecular weight indicated were presented in Supplementary Fig. 18. Each experiment was performed independently at least thrice. All blots were derived from the same experiment and were processed in parallel.

### Real-time PCR analysis

Total RNA was extracted using NucleoSpin® RNA Plus (Takara Bio, Shiga, Japan) and cDNA was synthesized using PrimeScript™ RT master mix (Perfect Real Time; Takara Bio) according to the respective manufacturer’s instructions. Real-time PCR was performed using a TaKaRa PCR thermal cycler Dice® (Takara Bio) and SYBR Fast qPCR kit (Kapa Biosystems, Cape Town, South Africa) using the following amplification protocol: initial incubation at 95 °C for 10 min, 40 cycles at 95 °C for 15 s and 60 °C for 1 min, followed by melting curve analysis. Gene expression was calculated from relative standard curves, normalized to GAPDH levels, and analyzed using the 2^−ΔΔCT^ method. The primer sequences used in this study are shown in Supplementary Table 4.

### Apoptotic cell death analysis

H2228 and A925L cells (1 × 10^5^) were treated with or without lorlatinib (100 nmol/L) and/or erlotinib (100 nmol/L) for 48 h. All floating and adherent cells were collected and incubated with Annexin V-FITC and propidium iodide for 15 min at 25 °C in the dark. Then, samples were analyzed using BD Accuri C6 Plus flow cytometer (Becton, Dickinson & Company, Franklin Lakes, NJ, USA) and data were analyzed using the FlowJo® software (FlowJo LLC, Ashland, OR, USA), as reported previously^39^.

### CDX models

A925L and H2228 cell suspensions in PBS (5 × 10^6^ cells) were injected subcutaneously into the flanks of 5-week-old male C.B-17/Icr-scid/scidJcl mice with severe combined immunodeficiency (Clea Japan, Tokyo, Japan). Once the mean tumor volume had reached approximately 100–200 mm^3^, mice were administered the targeted drugs via daily oral gavage. Body weight was measured twice weekly and general condition was monitored daily. The tumors were measured twice weekly using calipers and their volume was calculated as (width^2^ × length)/2. Mouse experimental protocols were approved by the institutional review board of Kyoto Prefectural University of Medicine (Kyoto, Japan; approval no. M29–529). Animal surgery was performed following anesthesia with sodium pentobarbital according to institutional guidelines, and efforts were made to minimize animal suffering.

### Tumor histological analyses

Similar to previous studies^18^, formalin-fixed, paraffin-embedded tissue sections (4-μm-thick) were deparaffinized and subjected to antigen retrieval by microwaving the tissue sections in 10 mM citrate buffer (pH 6.0). Proliferating cells were detected by incubation at 37 °C in 5% CO_2_ with Ki-67 antibodies (Clone MIB-1; GA62661-2, Dako). Apoptosis was quantitated using the terminal deoxynucleotidyl transferase-mediated dUTP-biotin nick-end labeling (TUNEL) method, according to the manufacturer’s instructions. Based on their expression patterns, tumor cells from the tissue specimens were separately evaluated for Bcl-xL, total-EGFR, and phospho-EGFR expression. After incubation with primary and secondary antibodies under the recommended conditions and treatment with a Vectastain ABC kit (Vector Laboratories, Burlingame, CA, USA), peroxidase activity was visualized using 3,3′-diaminobenzidine as a chromogen, and the sections were counterstained with hematoxylin. The five areas (0.1 mm^2^/section) containing the most positively stained cells in each section were selected for histological quantitation using light microscopy at 400 × magnification. The intensity and percentage of positive cells were determined. Total-EGFR classified into 1 + , 2+ as low group, and 3+ as high group, and phospho-EGFR expression classified into 0 and 1+ as low group, and 2+ and 3+ as high group, based on the staining intensity, as reported previously^40,41^.

### Patients

ALK-rearranged tumor specimens were obtained from 26 patients with NSCLC at University Hospital, Kyoto Prefectural University of Medicine (Kyoto, Japan), Japanese Red Cross Kyoto Daiichi Hospital (Kyoto, Japan), and Japanese Red Cross Kyoto Daini Hospital (Kyoto, Japan) prior to treatment with alectinib. The study was conducted in accordance with the Declaration of Helsinki. All patients provided written informed consent and each hospital study was approved by the institutional review board of University Hospital, Kyoto Prefectural University of Medicine (approval No. ERB-C-2684).

### Statistical analysis

Data from the MTT assays and xenograft tumor progression were expressed as the mean ± SD and the mean ± SEM, respectively. Significant differences were analyzed using two-way ANOVA, unpaired *t*-tests, one-way ANOVA. PFS, OS, and 95% confidence intervals were determined using the Kaplan−Meier method and compared using the log-rank test. Pearson’s correlation coefficients, and linear regression analysis of Prism 8.0 (GraphPad Software, San Diego, CA, USA). Two-sided *P*-values < 0.05 were considered significant.

### Reporting summary

Further information on research design is available in the Nature Research Reporting Summary linked to this article.

## Supplementary information

Supplementary Data

REPORTING SUMMARY

## Data Availability

Transcriptomic data obtained from the microarray analysis of parental cells and DT cells derived from A925L and H2228 cells have been deposited in Gene Expression Omnibus (GEO) under the accession code GSE188406. The histology images and patient survival data are not publicly available as they contain information that could compromise the privacy of the research participants. Qualified researchers can apply for access to these data by signing a data usage agreement. The other data that support the findings of this study are available on request from the corresponding author.
