# Supplementary material for: Adaptive resistance to lorlatinib via EGFR signaling in ALK-rearranged lung cancer
Source: NPJ Precis Oncol. 2023 Jan 26;7:12. doi: 10.1038/s41698-023-00350-7 (PMC9879975; doi:10.1038/s41698-023-00350-7)
Supplement: Supplementary file 1 — Supplementary Data [file 41698_2023_350_MOESM1_ESM.pdf]

Sup.Fig.1

a

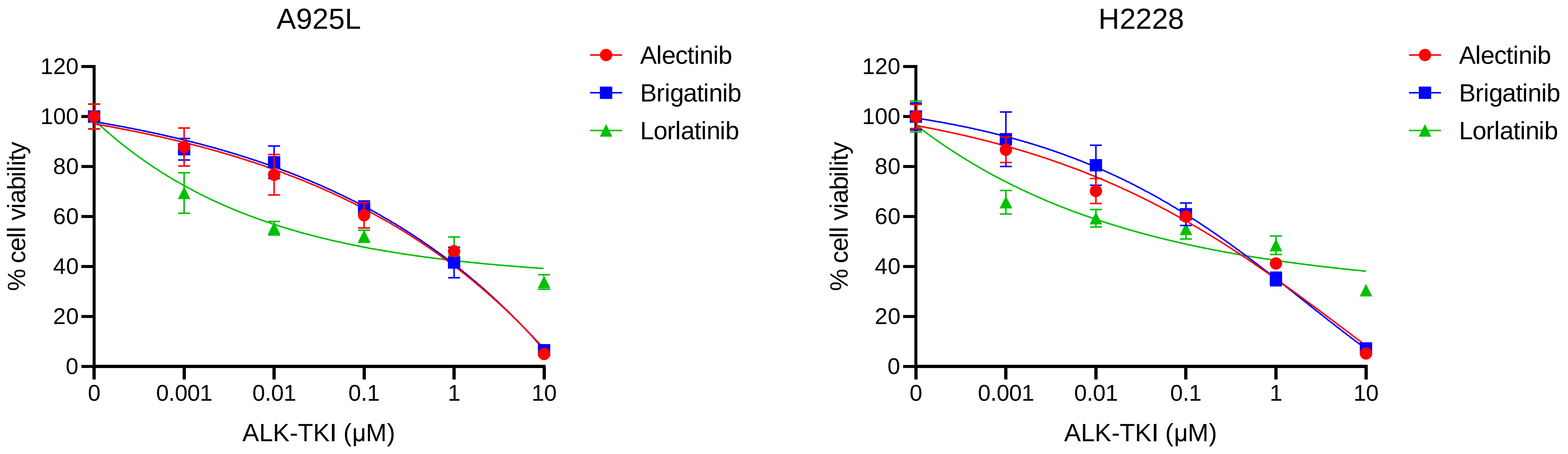

b

IC50 (95%CI) (nM)

	A925L	H2228
Alectinib	255 (160-404)	178 (117-269)
Brigatinib	283 (201-397)	218 (156-299)
Lorlatinib	156 (84-301)	178 (96-340)

Supplementary Figure 1. Cell viabilities of ALK-rearranged lung cancer cells treated with ALK-TKIs.
(a) A925L and H2228 cells were incubated with indicated concentrations of alectinib, brigatinib, and lorlatinib for 72 h. (b) The calculated IC50 values (95% confidence interval) of ALK-TKI in A925L and H2228 cells. Data are represented as mean ± S.D.

Sup.Fig.2

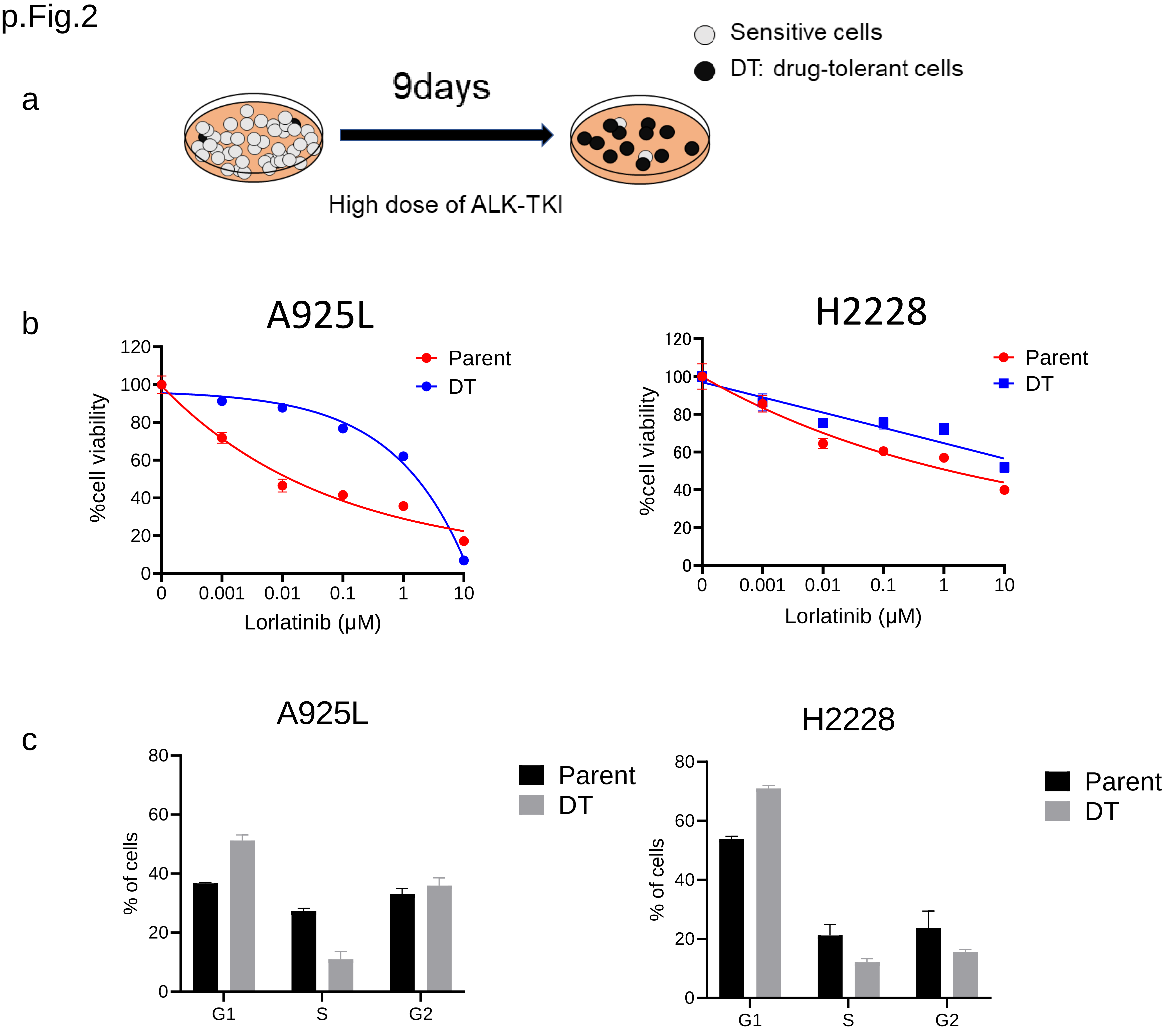

Supplementary Figure 2. Establishment of cells tolerant to lorlatinib (drug-tolerant (DT) cells) from ALK-rearranged NSCLC cells.

(a) DT cells generated from A925L and H2228 cells previously treated with 1 or 10 μmol/L lorlatinib for 9 days. (b) DT cells were treated with the indicated concentrations of lorlatinib for 72 h, and their viability was assessed using MTT assays. (c) Cell cycle analysis of DT cells and parent cells using flow cytometry with propidium iodide (PI) in A925L and H2228 cells. Data are represented as mean ± S.D.

Sup.Fig.3

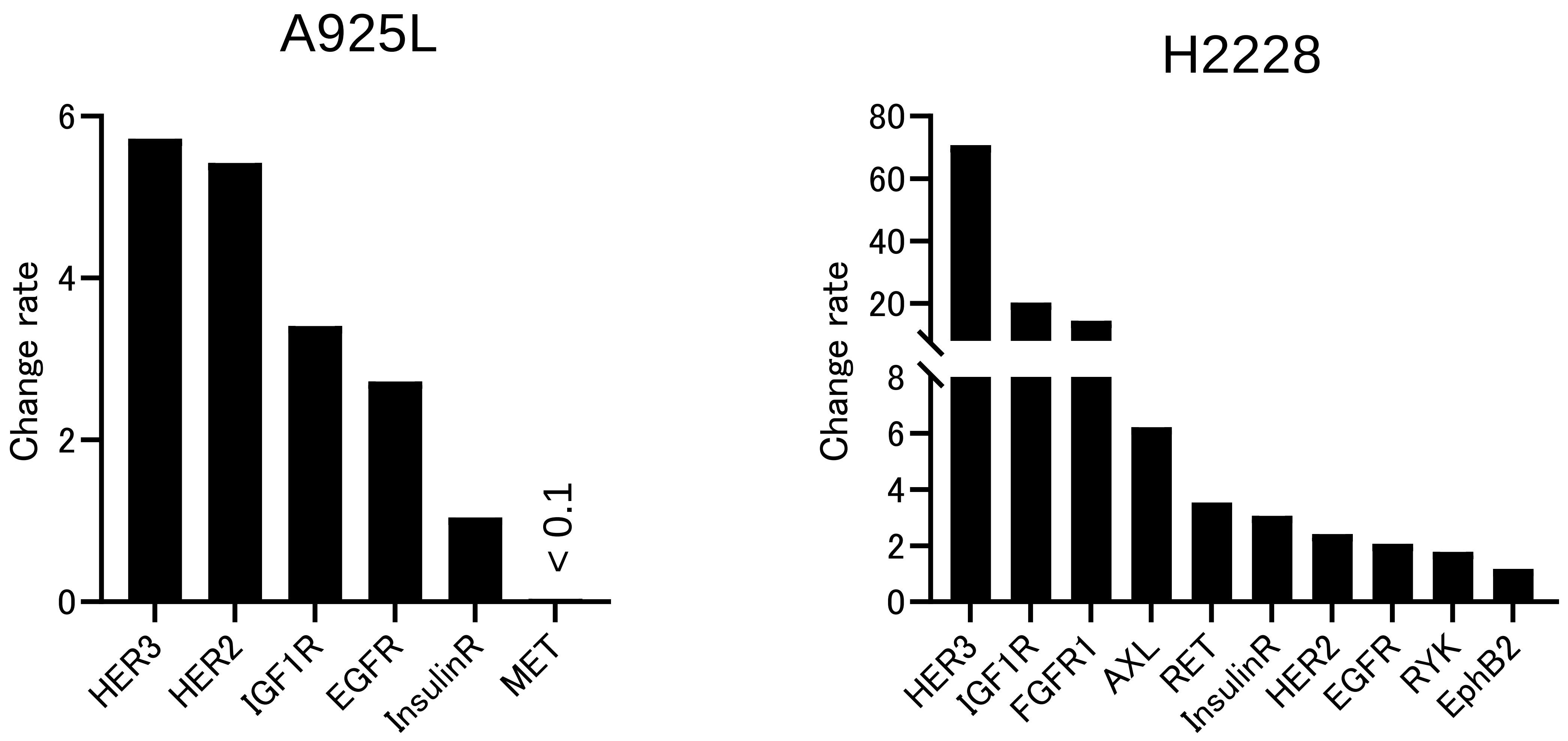

Supplementary Figure 3. Quantification of phospho-RTK array in parental cells and DT cells

Human phospho-RTK array analysis of parental cells and DT cells derived from A925L and H2228 cells. Mean pixel density was measured using ImageJ. RTKs not shown could not be detected.

Sup.Fig.4

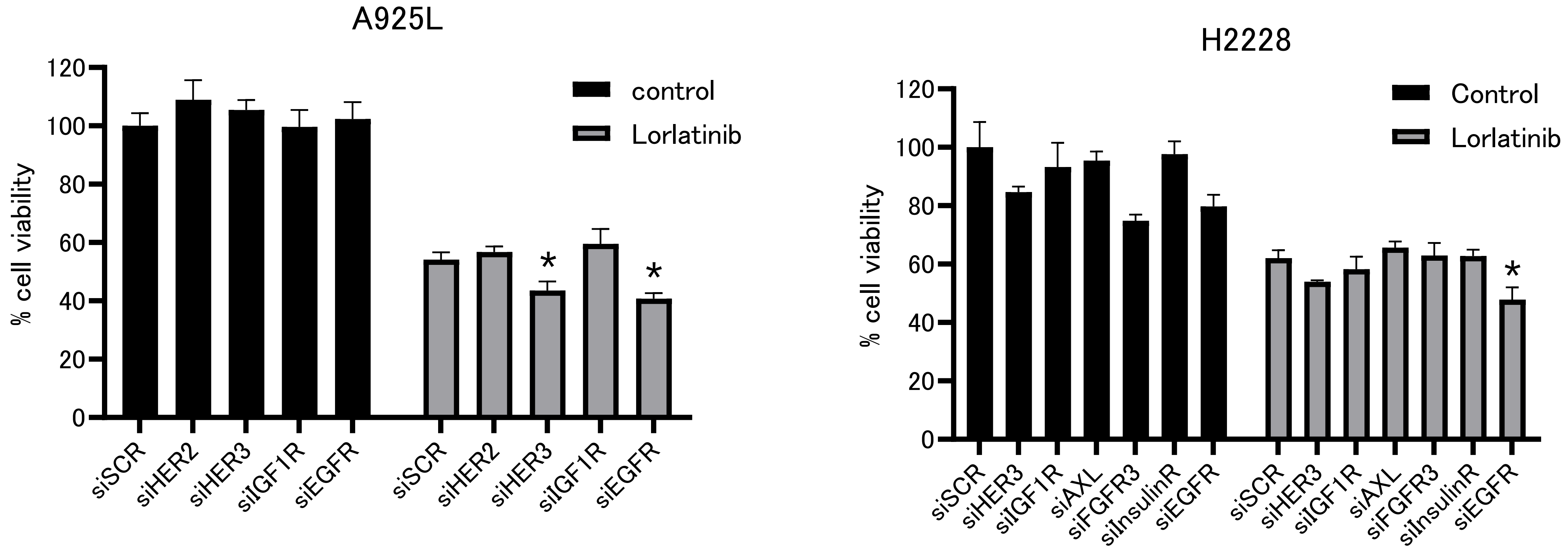

Supplementary Figure 4. Combination of lorlatinib and knockdown of RTKs upregulated in phospho-RTK array in ALK-rearranged NSCLC cells.

A925L and H2228 cells treated with nonspecific control siRNA or EGFR, HER2, HER3, IGF1R, AXL, FGFR3, and InsulinR specific siRNAs were incubated with or without lorlatinib (100 nmol/L) for 72 h and cell viability was detected using MTT assays. *, $P < 0.01$ compared with nonspecific control siRNA (two-way ANOVA). Data are represented as mean ± S.D.

Sup.Fig.5

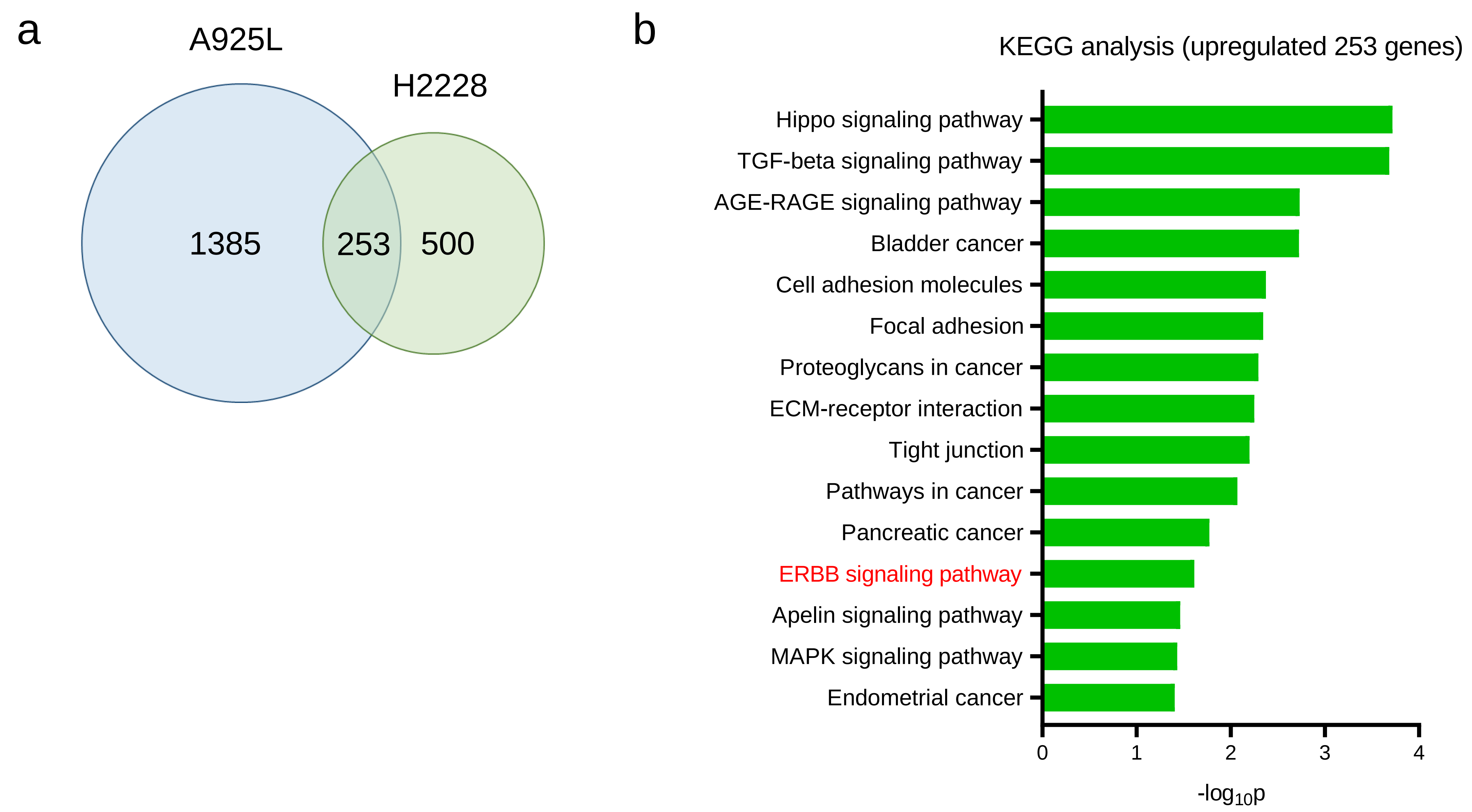

Supplementary Figure 5. Transcriptome analysis in A925L and H2228 DT cells using microarray analysis
(a) Venn plot showing genes upregulated in both A925L and H2228 DT cells. (b) KEGG analysis using commonly upregulated genes in A925L and H2228 DT cells.

Sup.Fig.6

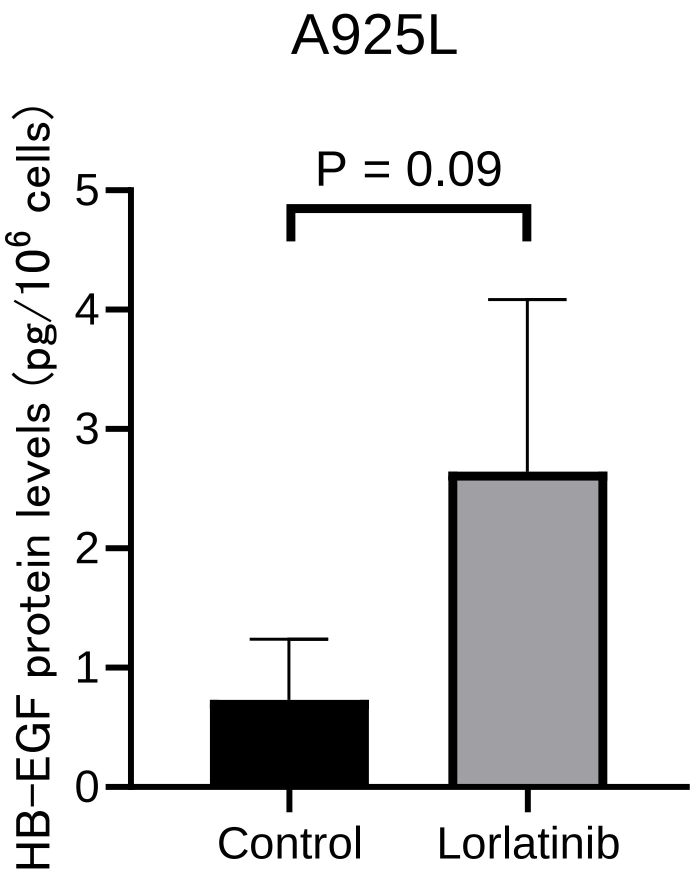

Supplementary Figure 6. Amount of HB-EGF in supernatant by ELISA in A925L cells

A925L (5 × 105 cells per 2 mL per well) were incubated for 48 h with or without lorlatinib (100 mmol/L), and culture supernatants were harvested. The levels of HB-EGF were measured by ELISA. Unpaired t-tests were used for comparisons. Data are represented as mean ± S.D.

Sup.Fig.7

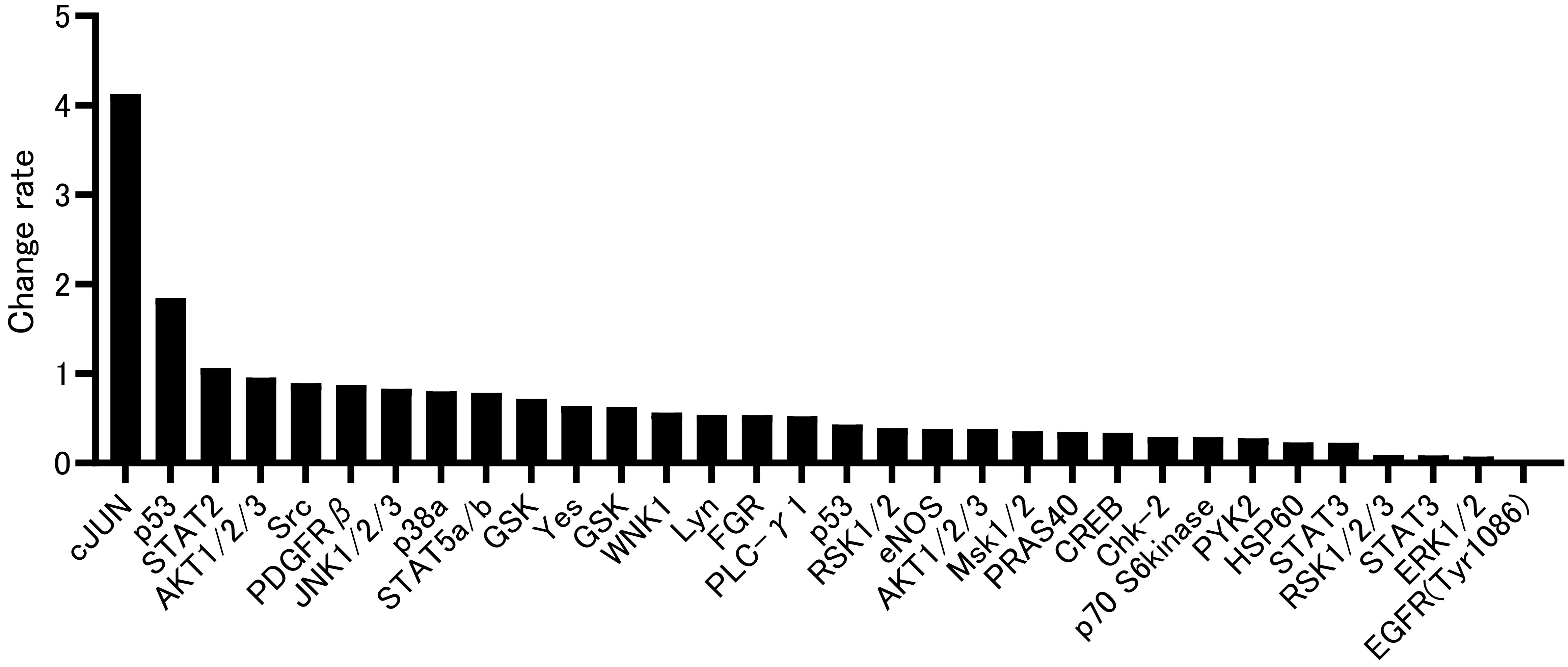

Supplementary Figure 7. Quantification of phospho-kinase array in A925L cells

Human phospho-kinase array analysis of parental A925L cells and A925L cells treated with lorlatinib (100 nmol/L) for 48 h. Mean pixel density was measured using ImageJ.

Sup.Fig.8

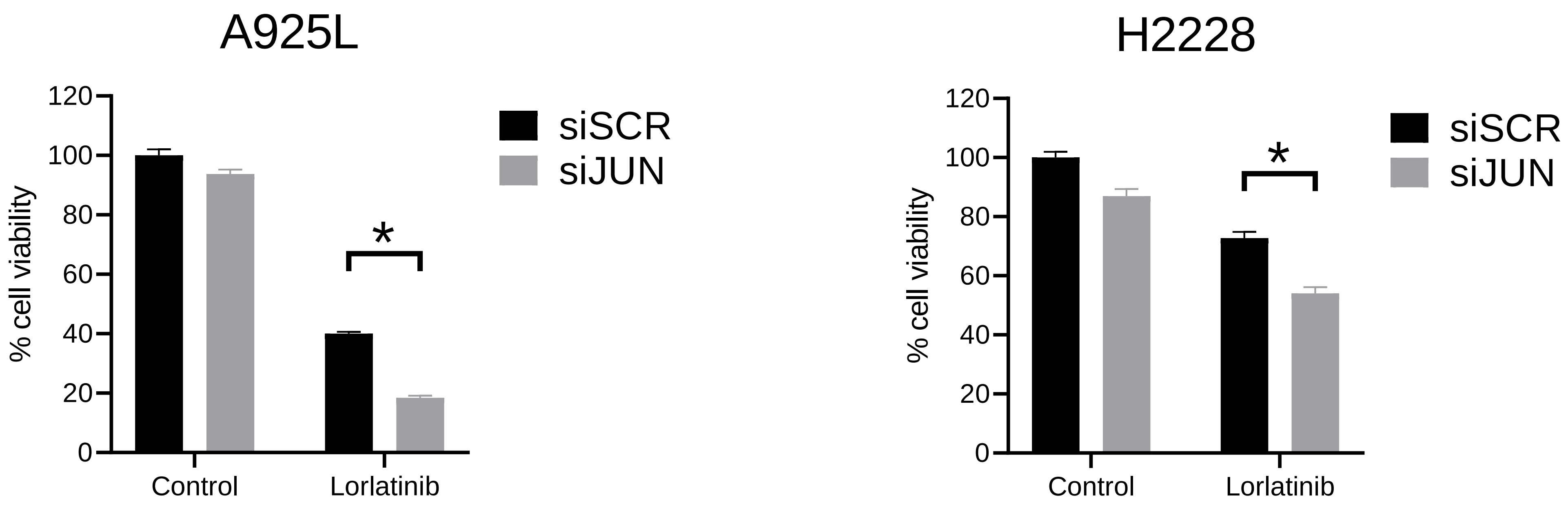

Supplementary Figure 8. Combination of lorlatinib and knockdown of JUN in ALK-rearranged NSCLC cells.

A925L and H2228 cells treated with nonspecific control siRNA or JUN-specific siRNAs were incubated with or without lorlatinib (100 nmol/L) for 72 h and cell viability was detected using MTT assays. *, $P < 0.01$ (two-way ANOVA). Data are represented as mean ± S.D.

Sup.Fig.9

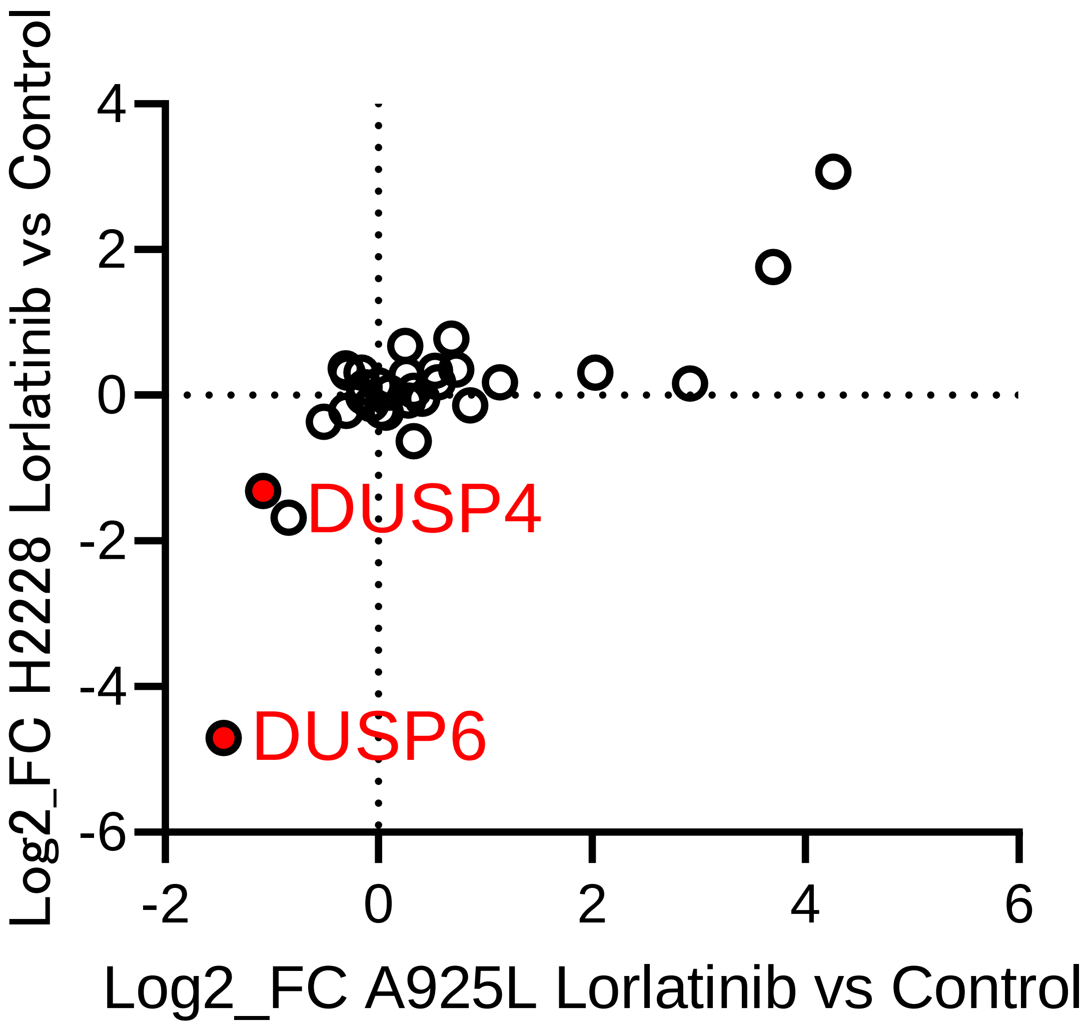

Supplementary Figure 9. DUSP gene expression in DT cells vs. that in parent cells

Scatterplot of DUSP gene expressions analyzed by microarray in either A925L or H2228 DT cells vs. that in parent cells. Samples are colored according to the significance of differential expression ($P < 0.05$) in both groups.

Sup.Fig.10

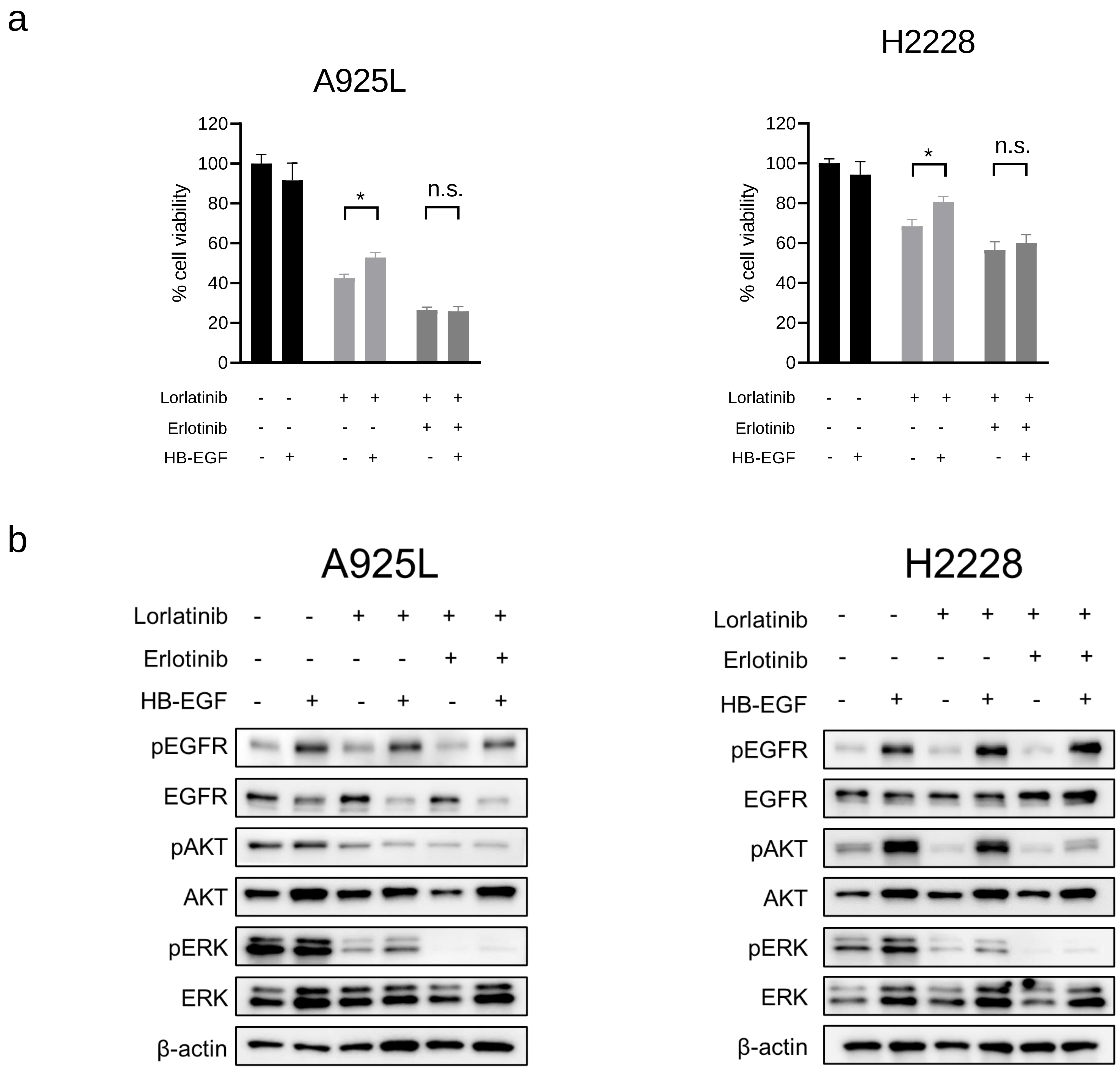

Supplementary Figure 10. HB-EGF-triggered resistance to lorlatinib is abrogated by erlotinib

(a) A925L and H2228 were treated with or without lorlatinib (100 nmol/L), erlotinib (100 nmol/L), or with a combination of lorlatinib and erlotinib and/or HB-EGF (50 ng/mL, 10 ng/mL, respectively) for 72 h. Cell growth was determined by MTT assays. *, $P < 0.01$ (two-way ANOVA). (b) Western blot of A925L and H2228 cells treated with or without lorlatinib (100 nmol/L), erlotinib (100 nmol/L), or with a combination of lorlatinib and erlotinib and/or HB-EGF (50 ng/mL, 10 ng/mL, respectively) for 4 h. Data are represented as mean ± S.D.

Sup.Fig.11

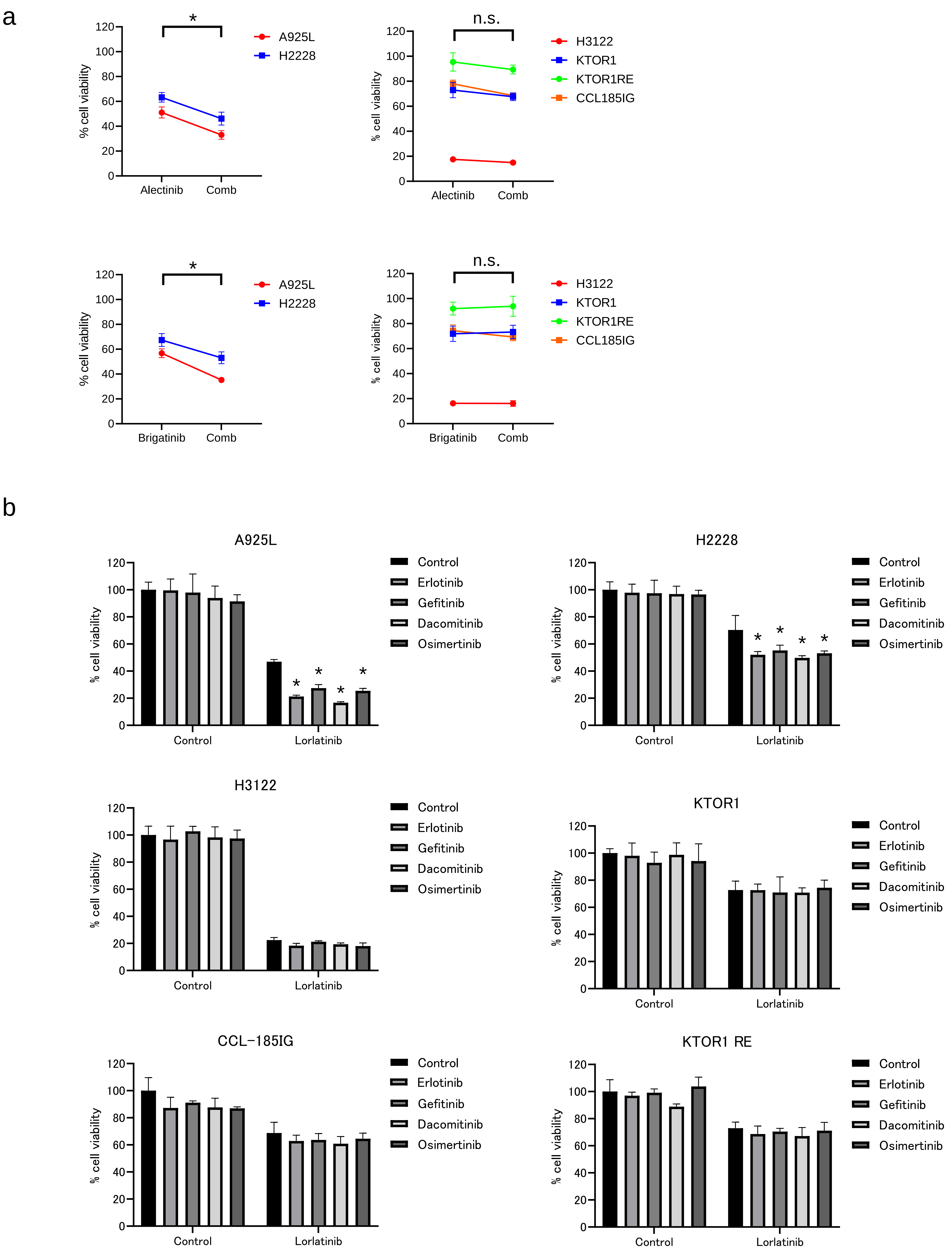
a
*
n.s.
% cell viability
Alectinib
Comb
Brigatinib
A925L
H2228
H3122
KTOR1
KTOR1RE
CCL185IG
b
A925L
H2228
H3122
KTOR1
CCL-185IG
KTOR1 RE
Control
Erlotinib
Gefitinib
Dacomitinib
Osimertinib
Lorlatinib

Sup.Fig.11

c

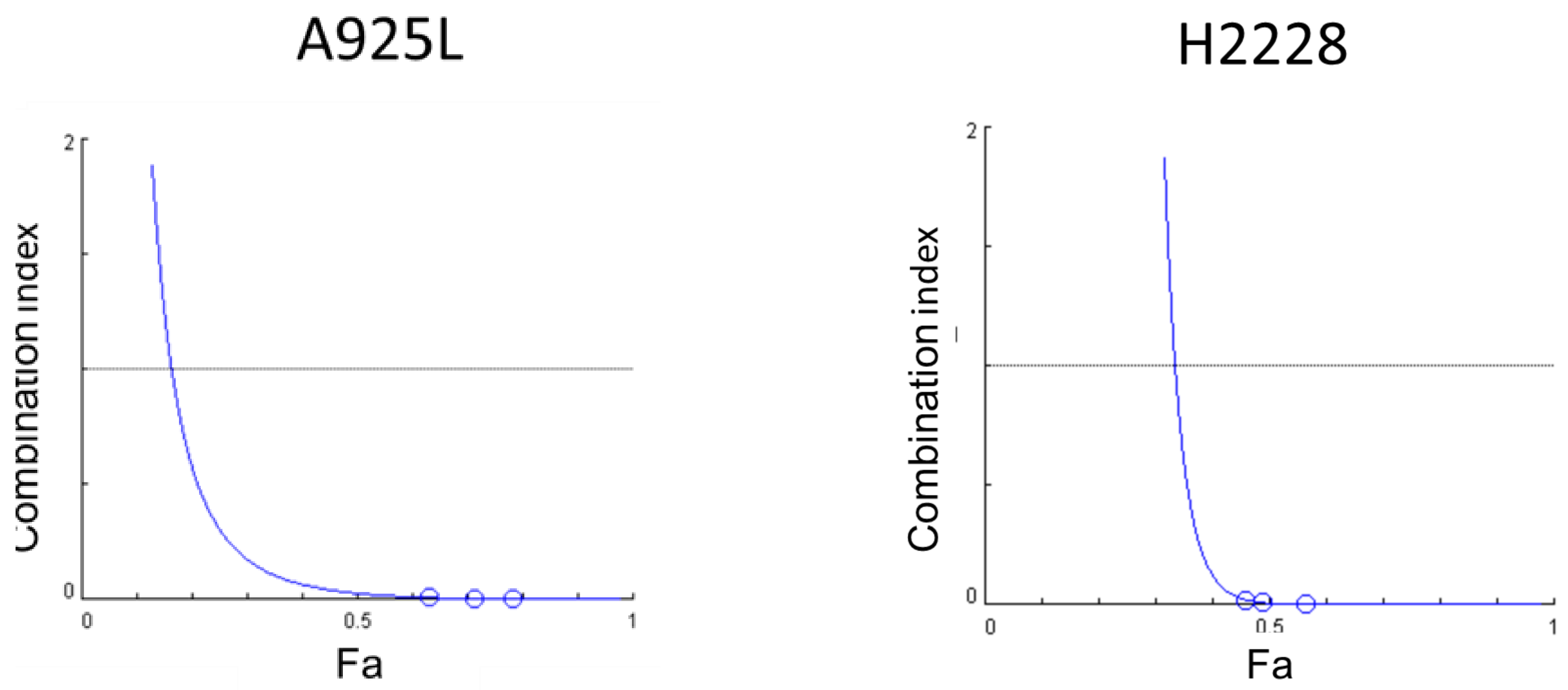

d

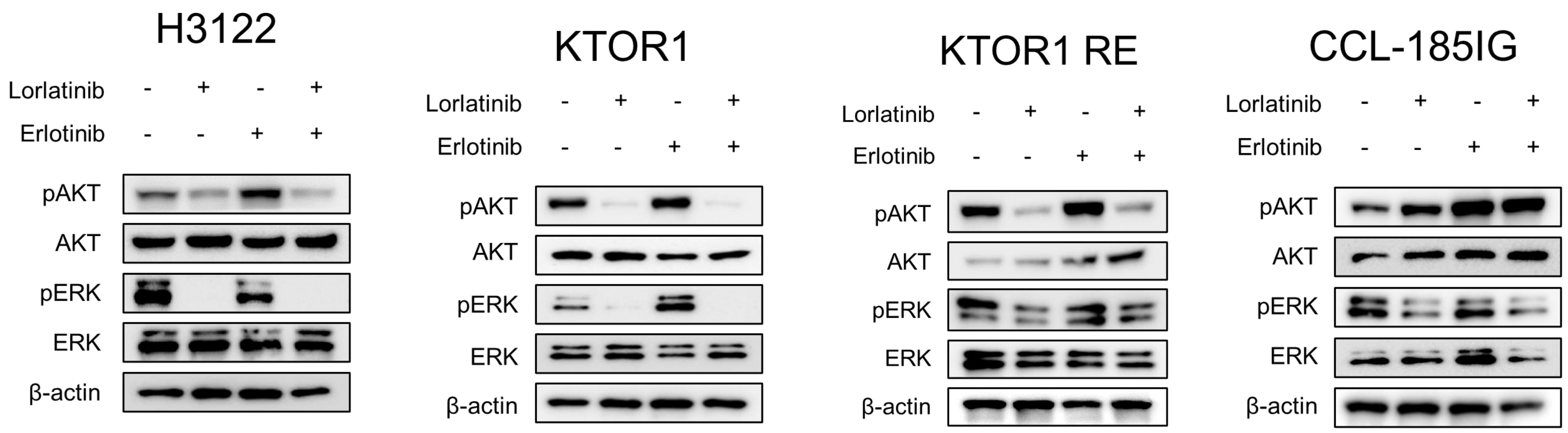

Supplementary Figure 11. Combination therapy with ALK-TKIs and EGFR-TKIs in ALK-rearranged NSCLC cells

(a) Quantitative determination of the inhibition of cell viability of high-EGFR-expressing and low-EGFR-expressing ALK-rearranged NSCLC cells treated with the alectinib or brigatinib in the presence or absence of erlotinib. *, P < 0.05 (paired Student's t-test). (b) A925L, H2228, H3122, KTOR1, KTOR1 RE, and CCL-185IG cells were treated with the 100 nmol/L EGFR-TKI erlotinib, gefitinib, dacomitinib, or osimertinib, 100 nmol/L of lorlatinib, or a combination of these for 72 h, and the cell viability was assessed using MTT assays. *, P < 0.01 compared with the results of lorlatinib monotherapy (two-way ANOVA. (c) Combination index (CI) values were analyzed according to the Chou and Talalay equation using the CalcuSyn software. (d) Western blot of H3122, KTOR1, KTOR1RE, and CCL-185IG cells treated with lorlatinib (100 nmol/L), erlotinib (100 nmol/L), or a combination of lorlatinib and erlotinib for 4 h. Data are represented as mean ± S.D.

Sup.Fig.12

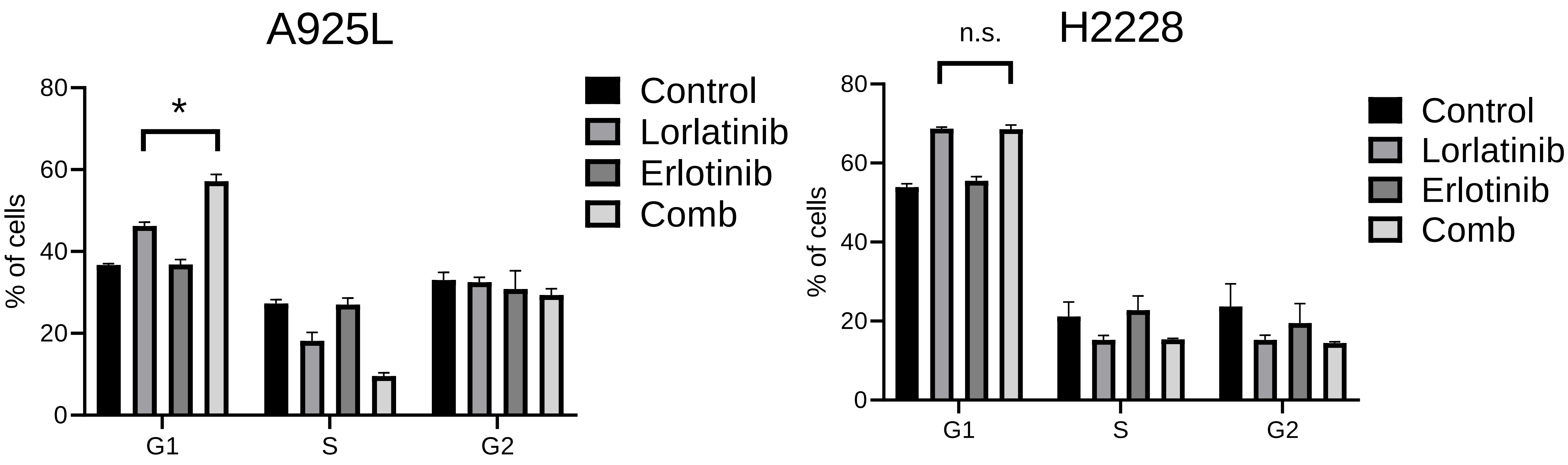

Supplementary Figure 12. Cell cycle analysis in ALK-rearranged NSCLC cells.

A925L and H2228 cells were treated with lorlatinib (100 nmol/L), erlotinib (100 nmol/L), or a combination of lorlatinib and erlotinib for 48 h and the cell cycle was analyzed via flow cytometry with PI. Results are shown as the percentage of cell populations in various cell cycle phases. Comparison between lorlatinib monotherapy and combination with erlotinib. P-values were calculated using two-way ANOVA. Data are represented as mean ± S.D.

Sup.Fig.13

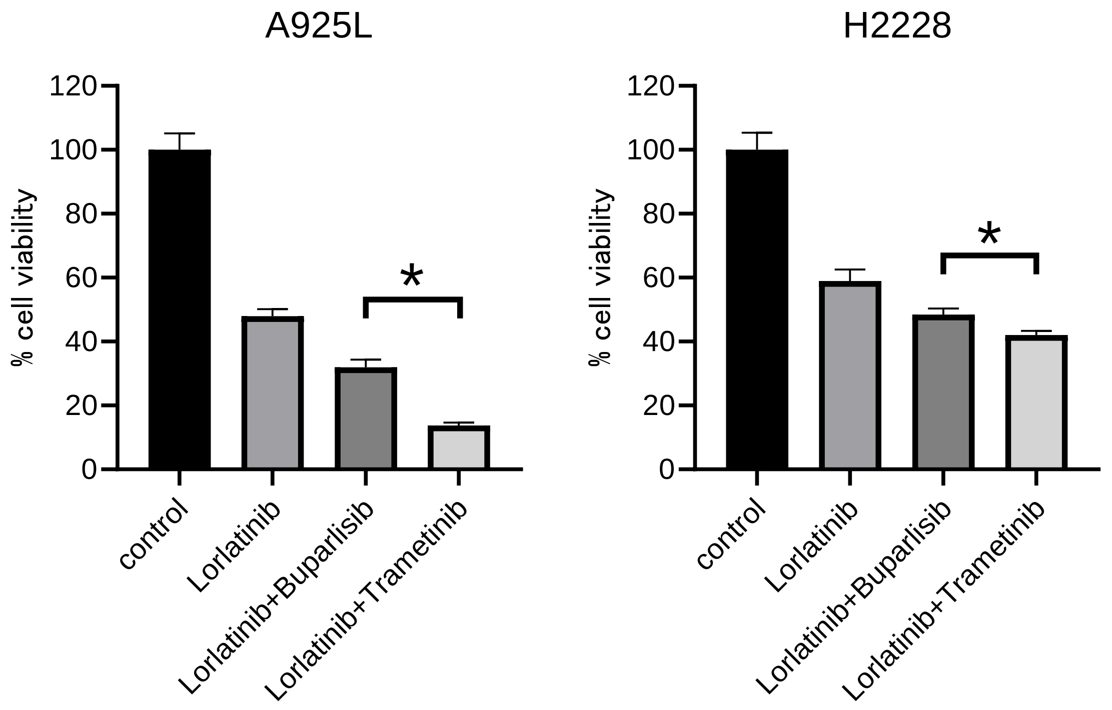

Supplementary Figure 13. Combination of lorlatinib and trametinib or buparlisib in ALK-rearranged NSCLC cells.

A925L and H2228 cells with lorlatinib (100 nmol/L), a combination of lorlatinib and buparlisib (100 nmol/L), or a combination of lorlatinib and trametinib (100 nM) for 72 h. Cell growth was determined using MTT assays. *, $P < 0.05$ (one-way ANOVA). Data are represented as mean ± S.D.

Sup.Fig.14

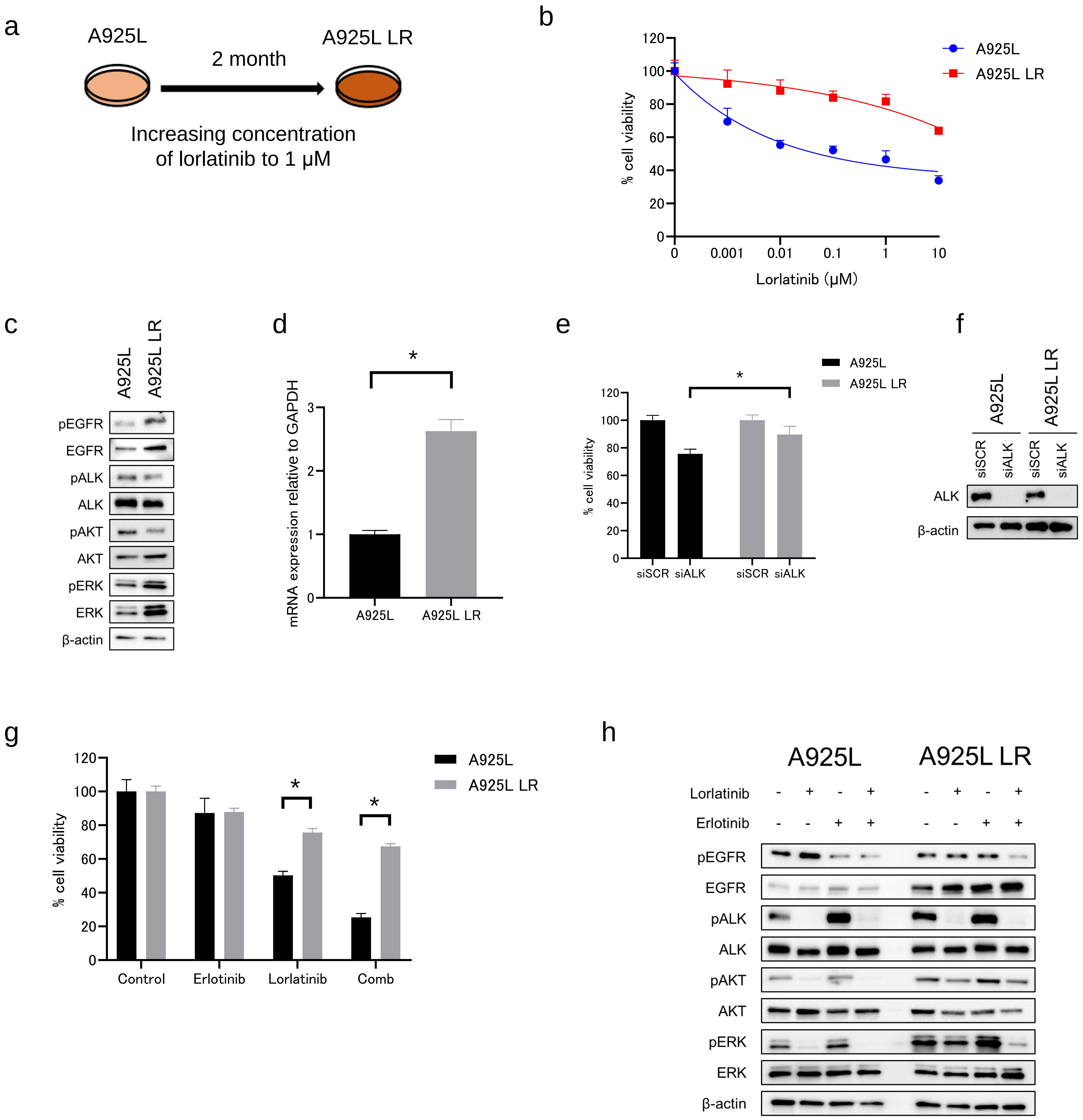
a
A925L
2 month
A925L LR
Increasing concentration
of lorlatinib to 1 μM
b
120
100
80
60
40
20
0
% cell viability
0
0.001
0.01
0.1
1
10
Lorlatinib (μM)
A925L
A925L LR
c
A925L
A925L LR
pEGFR
EGFR
pALK
ALK
pAKT
AKT
pERK
ERK
β-actin
d
*
mRNA expression relative to GAPDH
3
2
1
0
A925L
A925L LR
e
A925L
A925L LR
*
120
100
80
60
40
20
0
% cell viability
siSCR siALK
siSCR siALK
f
A925L
A925L LR
siSCR
siALK
siSCR
siALK
ALK
β-actin
g
120
100
80
60
40
20
0
% cell viability
*
*
Control
Erlotinib
Lorlatinib
Comb
A925L
A925L LR
h
A925L
A925L LR
Lorlatinib - + - + - + - +
Erlotinib - - + + - - + +
pEGFR
EGFR
pALK
ALK
pAKT
AKT
pERK
ERK
β-actin

Supplementary Figure 14. Acquired resistant ALK-rearranged lung cancer cells are less dependent on EGFR activity

(a) Plated parental A925L cells (left) and lorlatinib-acquired resistant A925L LR cells (right). A925L LR cells were generated using lorlatinib in vitro step-wise methods for 2 months. (b) Growth inhibition was assessed by MTT assay of A925L and A925L LR cells treated with the indicated concentrations of lorlatinib for 72 h. (c) Protein expression was analyzed by Western blotting with the indicated antibodies. (d) qPCR EGFR in A925L and A925L LR cells. *, $P < 0.01$ (unpaired t-tests). (e) A925L and A925L LR cells treated with nonspecific control siRNA or ALK-specific siRNAs were incubated for 72 h and cell viability was detected using MTT assays. *, $P < 0.01$ (two-way ANOVA). (f) A925L and A925L LR cells were incubated with nonspecific control siRNA or ALK-specific siRNA for 48 h, lysed, and the indicated proteins were detected via western blotting. (g) Growth inhibition assessed by MTT assay of A925L and A925L LR cells treated with 100 nmol/L lorlatinib or 100 nmol/L erlotinib, or a combination of these agents for 72 h. *, $P < 0.01$. (h) The indicated cells were incubated with 100 nmol/L lorlatinib or 100 nmol/L erlotinib, or a combination of these agents for 4 h. The cells were lysed, and the indicated proteins were detected by western blotting. Data are represented as mean ± S.D.

Sup.Fig.15

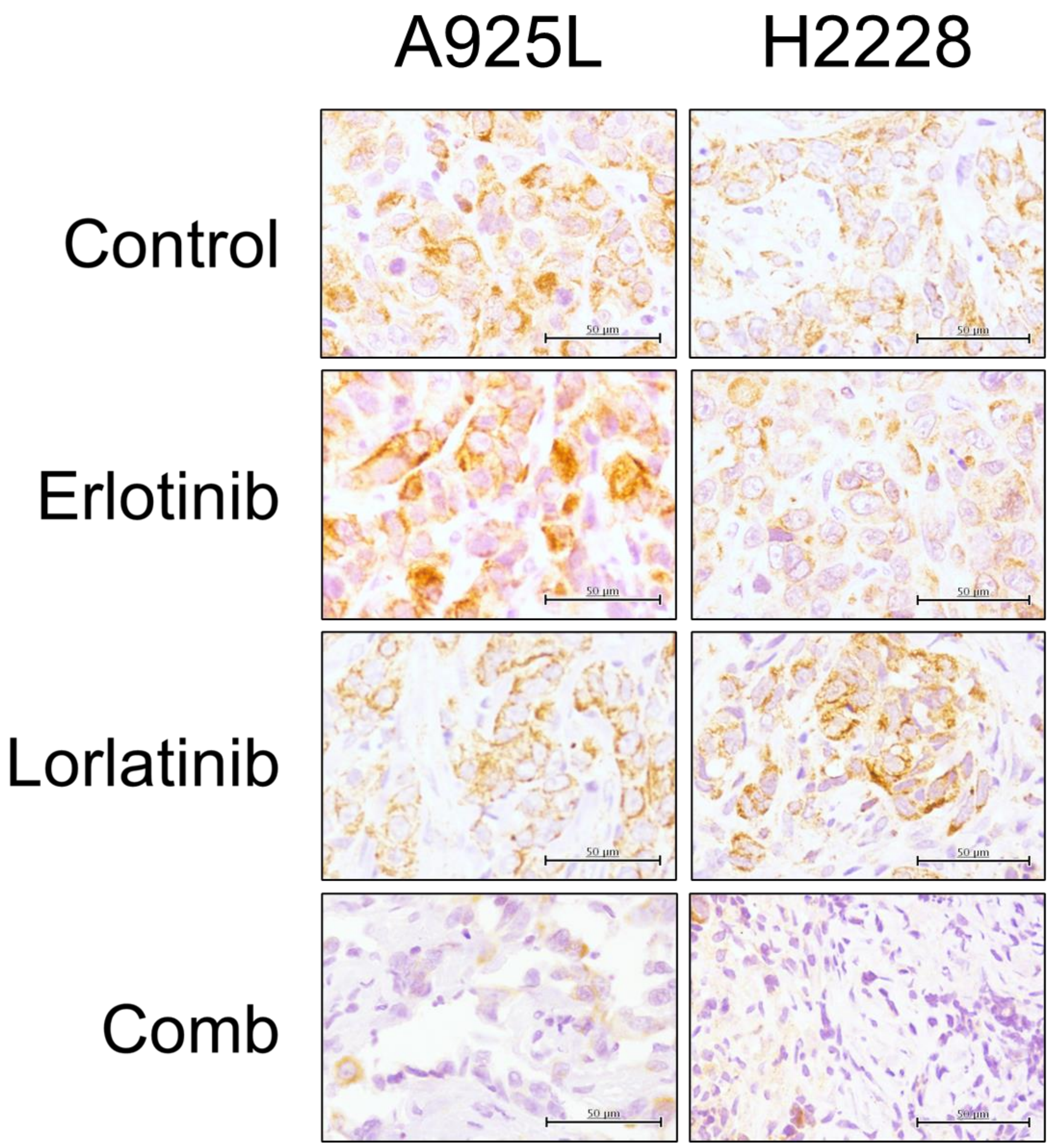

Supplementary Figure 15. Bcl-xL expressions in A925L and H2228 CDX tumors

Representative immunohistochemistry staining for Bcl-xL expression in A925L and H2228 CDX tumors treated with a vehicle (control), lorlatinib, erlotinib, or lorlatinib plus erlotinib for four days. Scale bar, 50 µm.

Sup.Fig.16

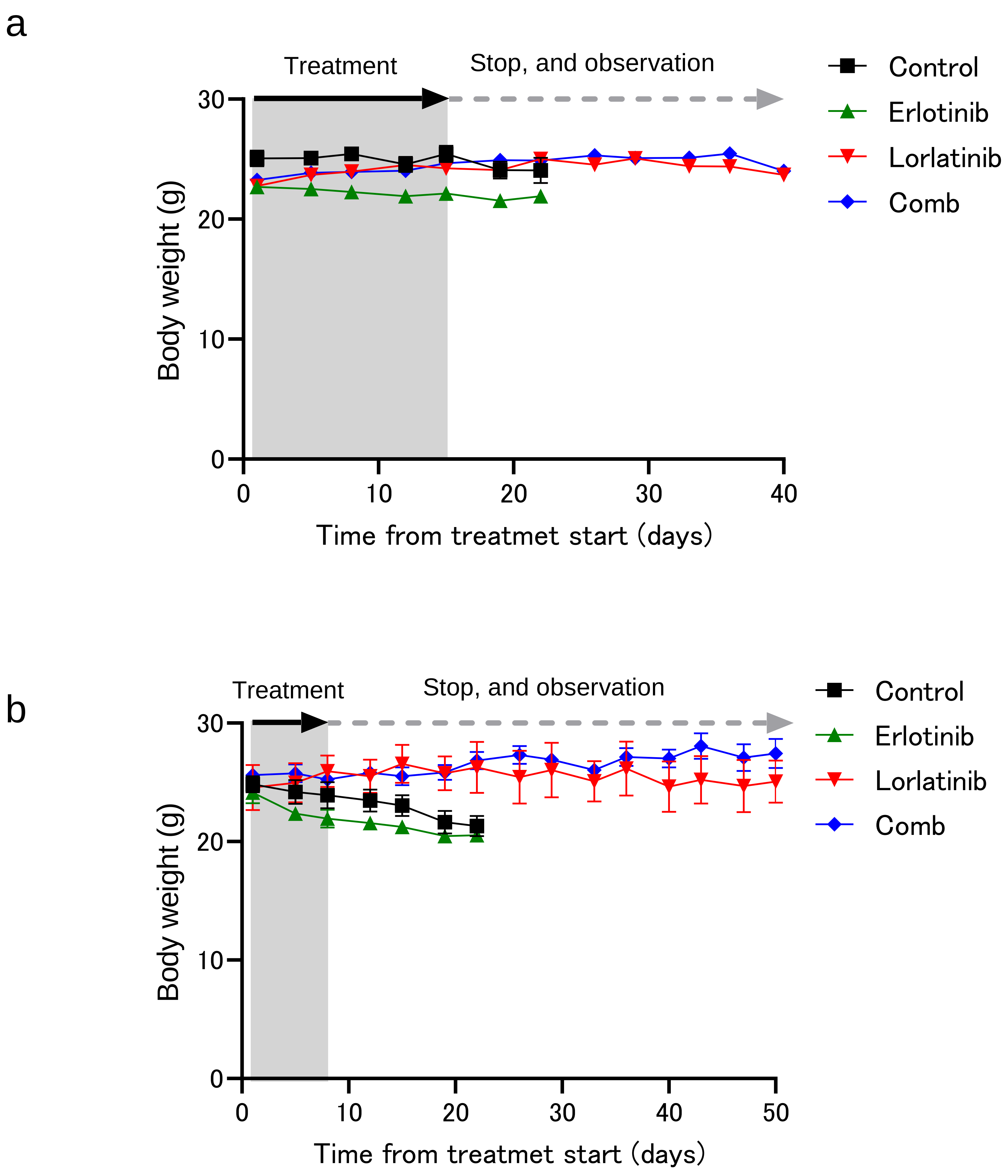

Supplementary Figure 16. Therapeutic tolerance in xenograft models of human ALK-rearranged NSCLC. Mouse weight was evaluated twice weekly.

(a) A925L CDX tumors were treated with a vehicle (control), lorlatinib (5 mg/kg), erlotinib (25 mg/kg), or lorlatinib (5 mg/kg) plus erlotinib (25 mg/kg) via daily oral gavage. (b) H2228 CDX tumors were treated with a vehicle (control), lorlatinib (1.5 mg/kg), erlotinib (25 mg/kg), or lorlatinib (1.5 mg/kg) plus erlotinib (25 mg/kg) via daily oral gavage. Data are represented as mean ± SD.

Sup.Fig.17

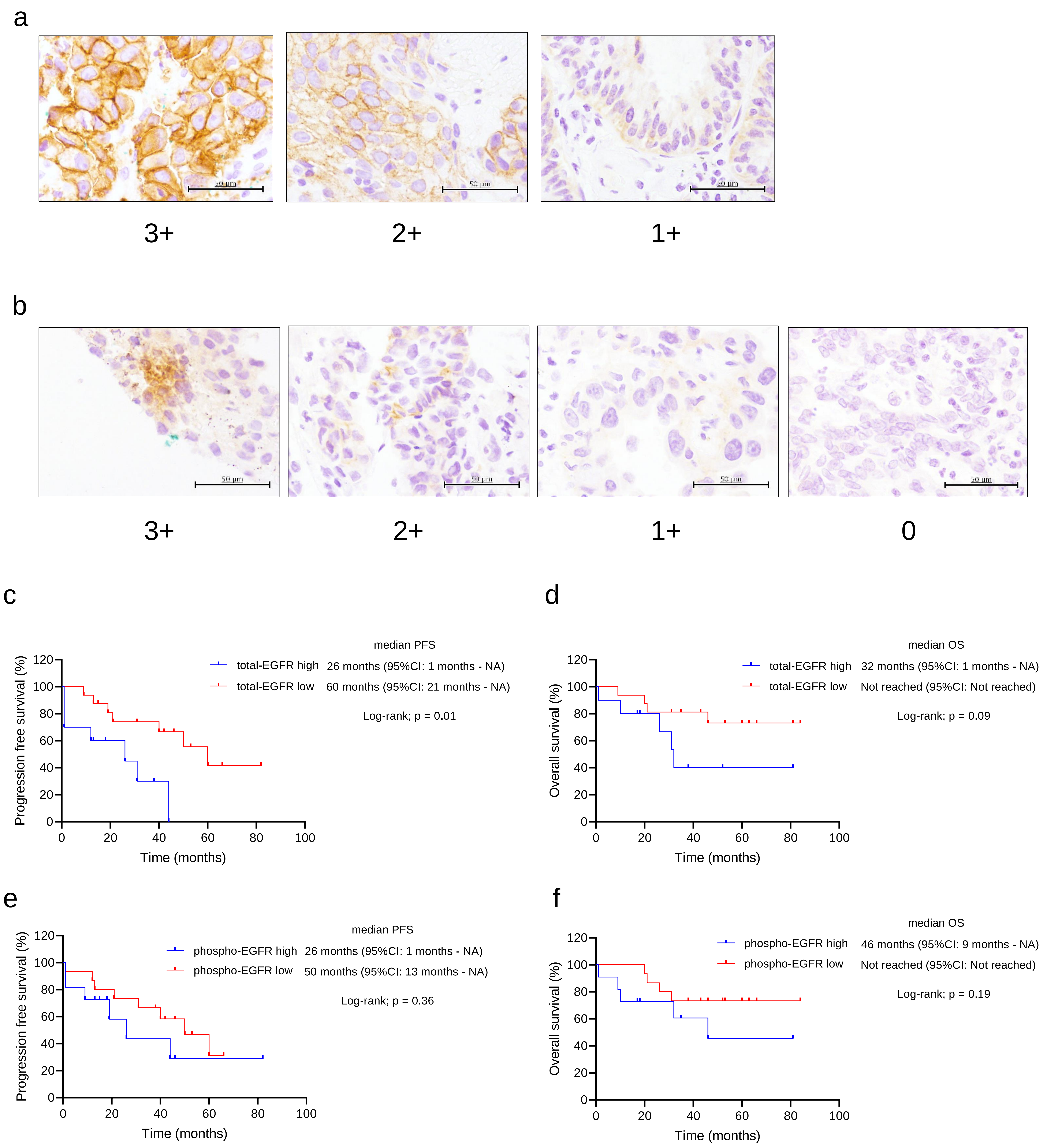
a
3+
2+
1+
b
3+
2+
1+
0
c
median PFS
total-EGFR high
26 months (95%CI: 1 months - NA)
total-EGFR low
60 months (95%CI: 21 months - NA)
Log-rank; p = 0.01
Progression free survival (%)
Time (months)
d
median OS
total-EGFR high
32 months (95%CI: 1 months - NA)
total-EGFR low
Not reached (95%CI: Not reached)
Log-rank; p = 0.09
Overall survival (%)
Time (months)
e
median PFS
phospho-EGFR high
26 months (95%CI: 1 months - NA)
phospho-EGFR low
50 months (95%CI: 13 months - NA)
Log-rank; p = 0.36
Progression free survival (%)
Time (months)
f
median OS
phospho-EGFR high
46 months (95%CI: 9 months - NA)
phospho-EGFR low
Not reached (95%CI: Not reached)
Log-rank; p = 0.19
Overall survival (%)
Time (months)

Supplementary Figure 17. Clinical significance of total-EGFR and phospho-EGFR expression in the outcome of alectinib treatment in patients with ALK-rearranged NSCLC.

a, b. Representative immunohistochemistry images of clinical specimens stained with total-EGFR (a) and phospho-EGFR (b) antibodies. Scale bar, 50 μm. c-f. Kaplan–Meier analysis of progression-free survival (PFS) and overall survival (OS) following alectinib treatment based on the status of total-EGFR and phospho-EGFR expression. PFS and OS were stratified according to total-EGFR expression (c, d). PFS and OS were stratified according to phospho-EGFR expression (e, f), respectively.

Sup.Fig.18

Fig.1f

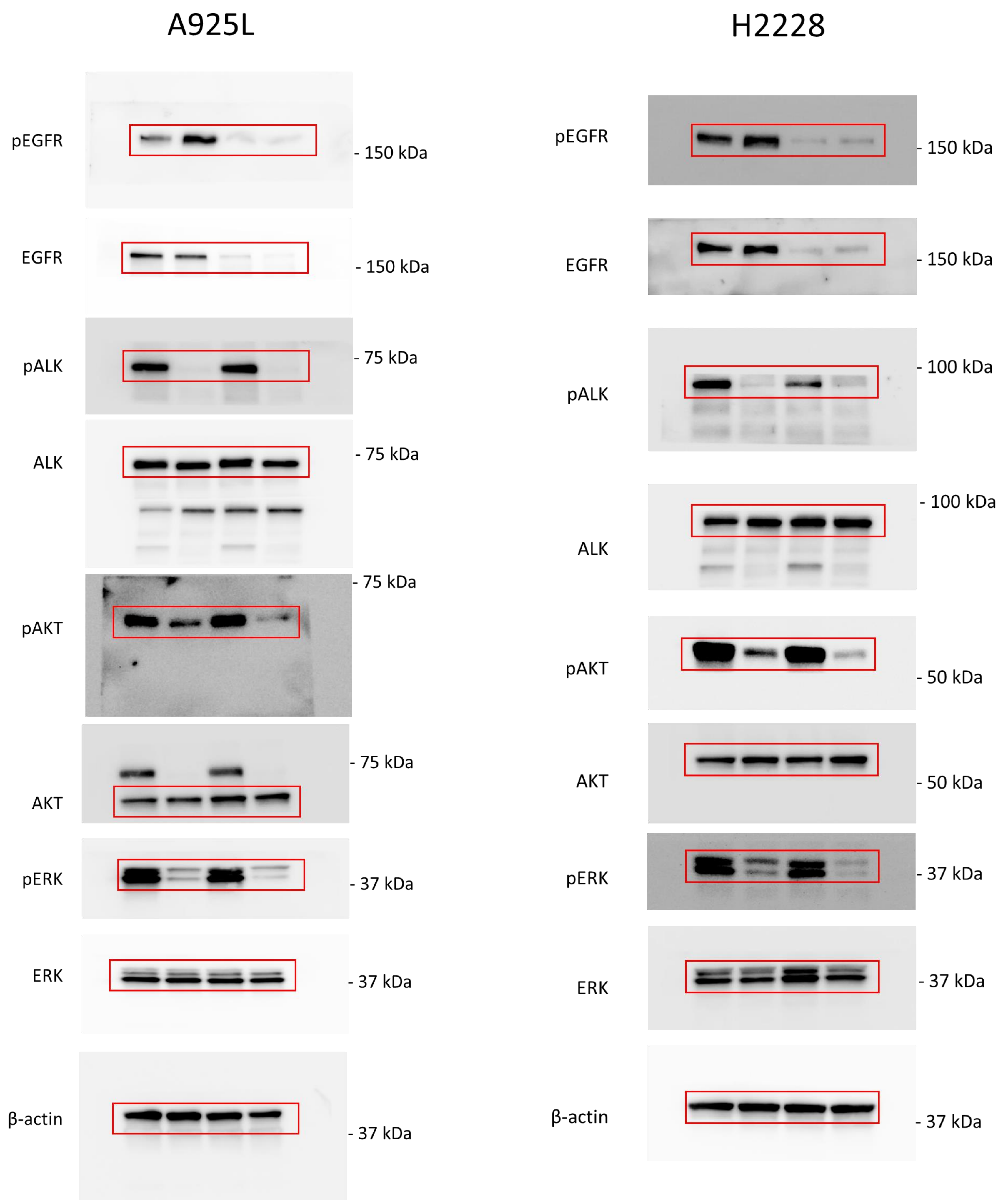

Fig.2a

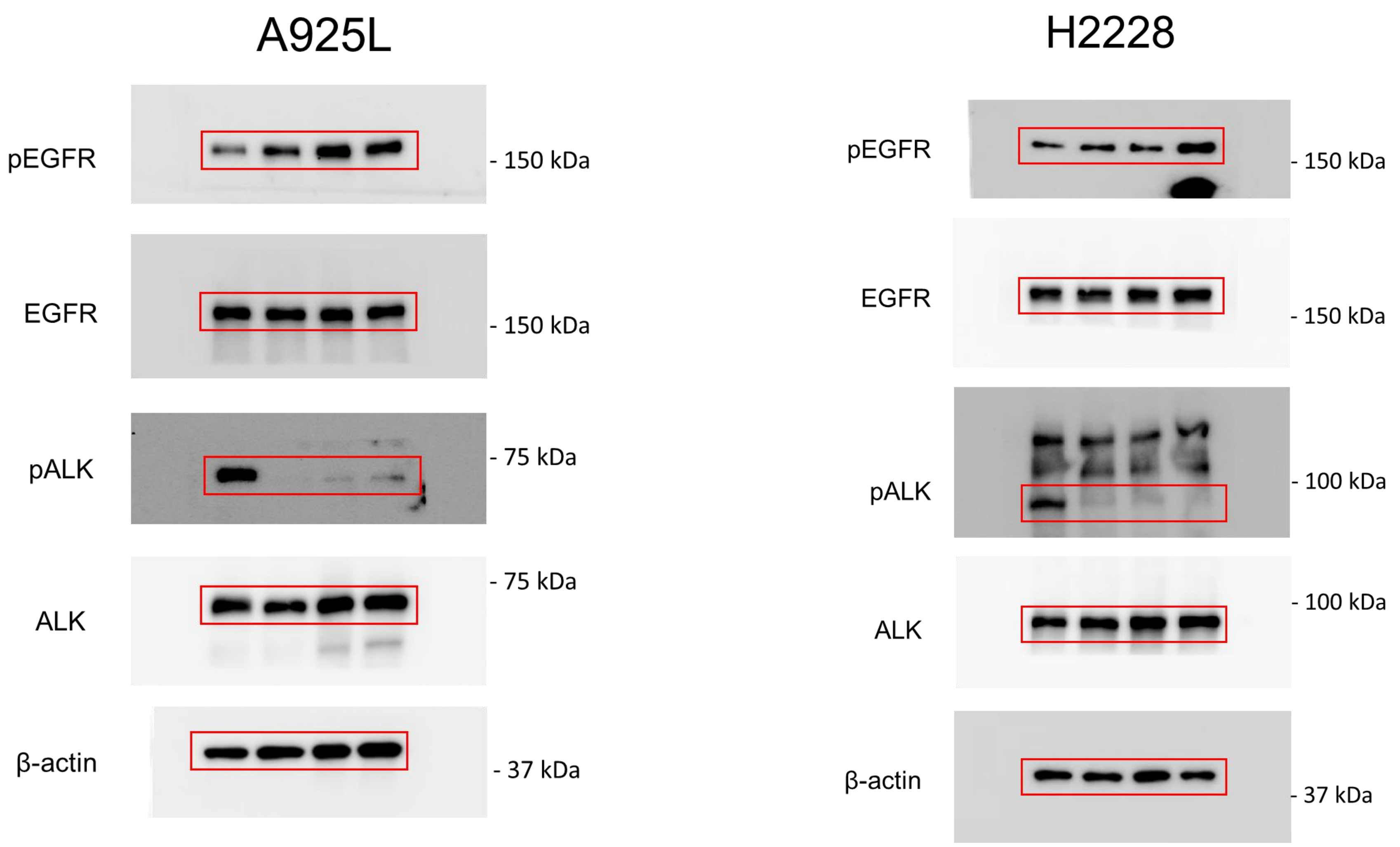

Sup.Fig.18

Fig.2f

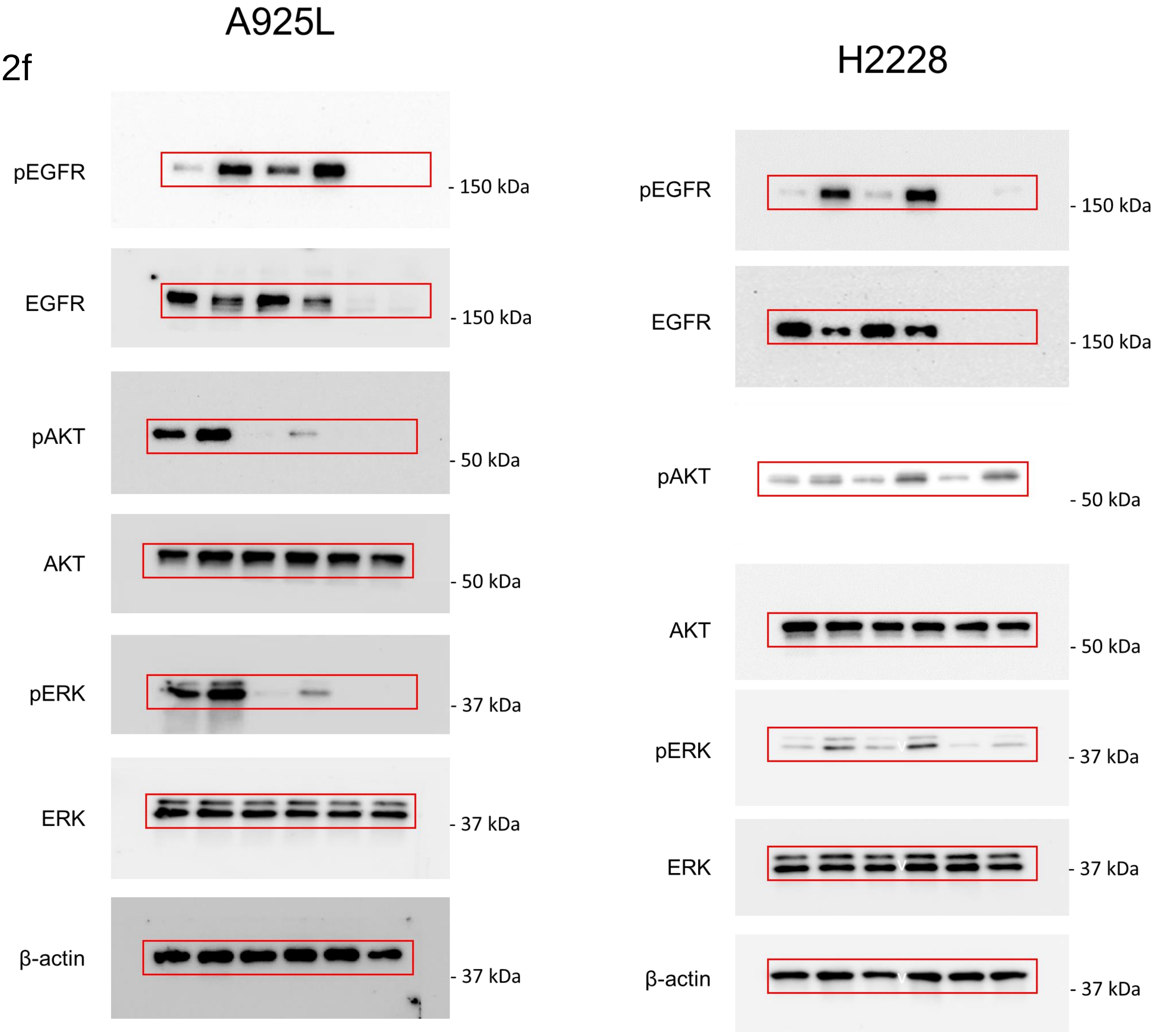

Fig.3c

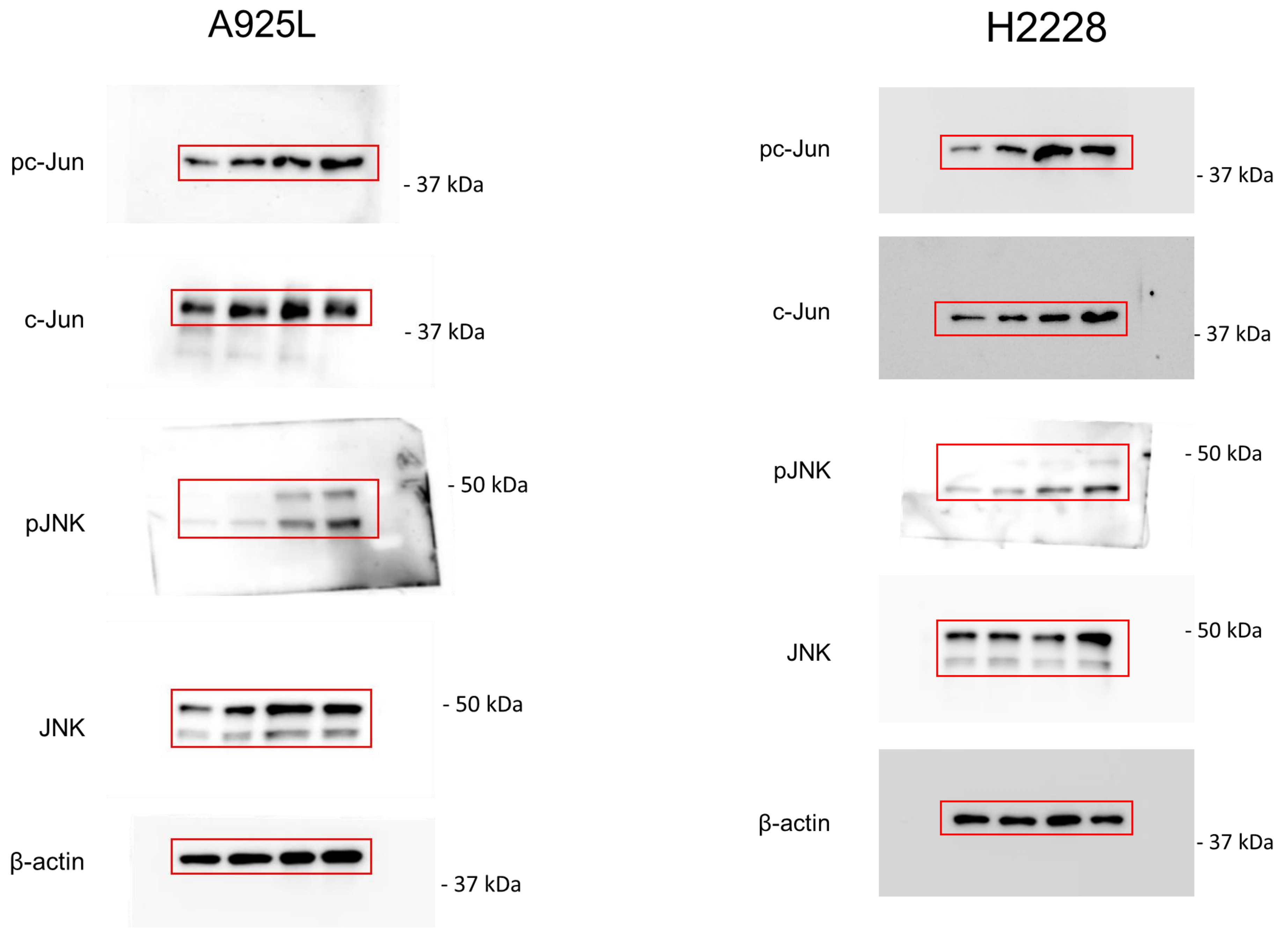

Sup.Fig.18

Fig.3d

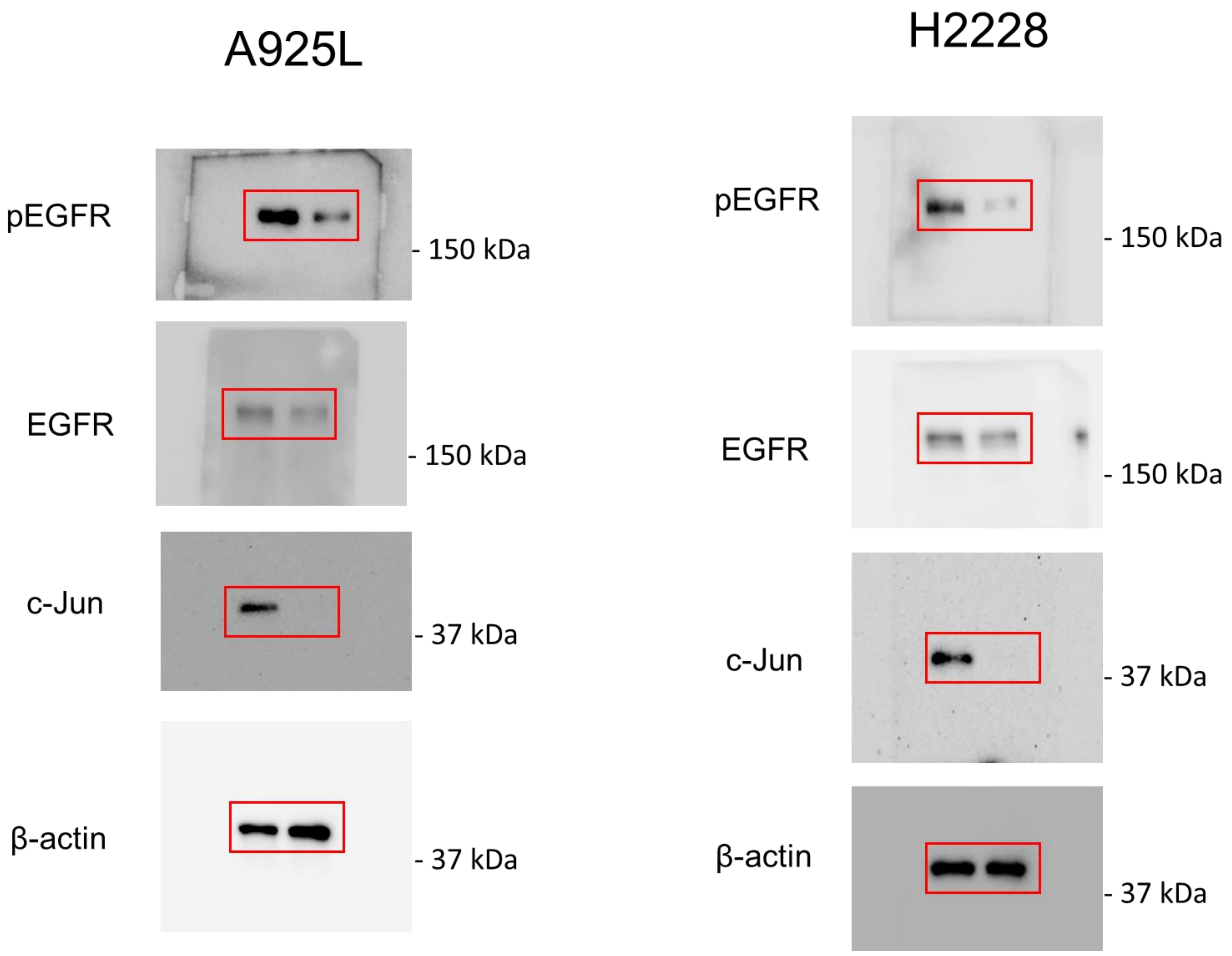

Fig.3f

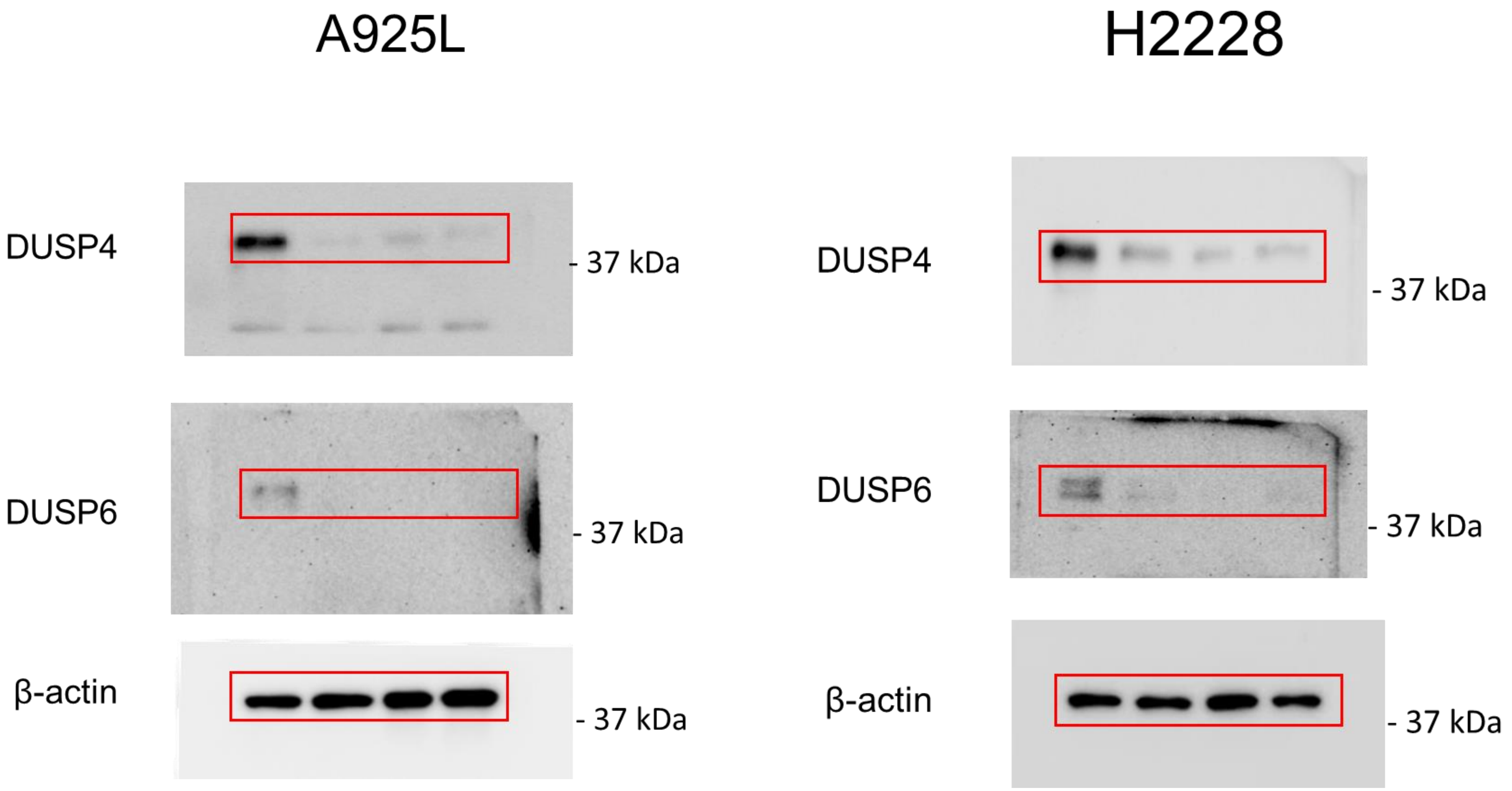

Sup.Fig.18

Fig.3g

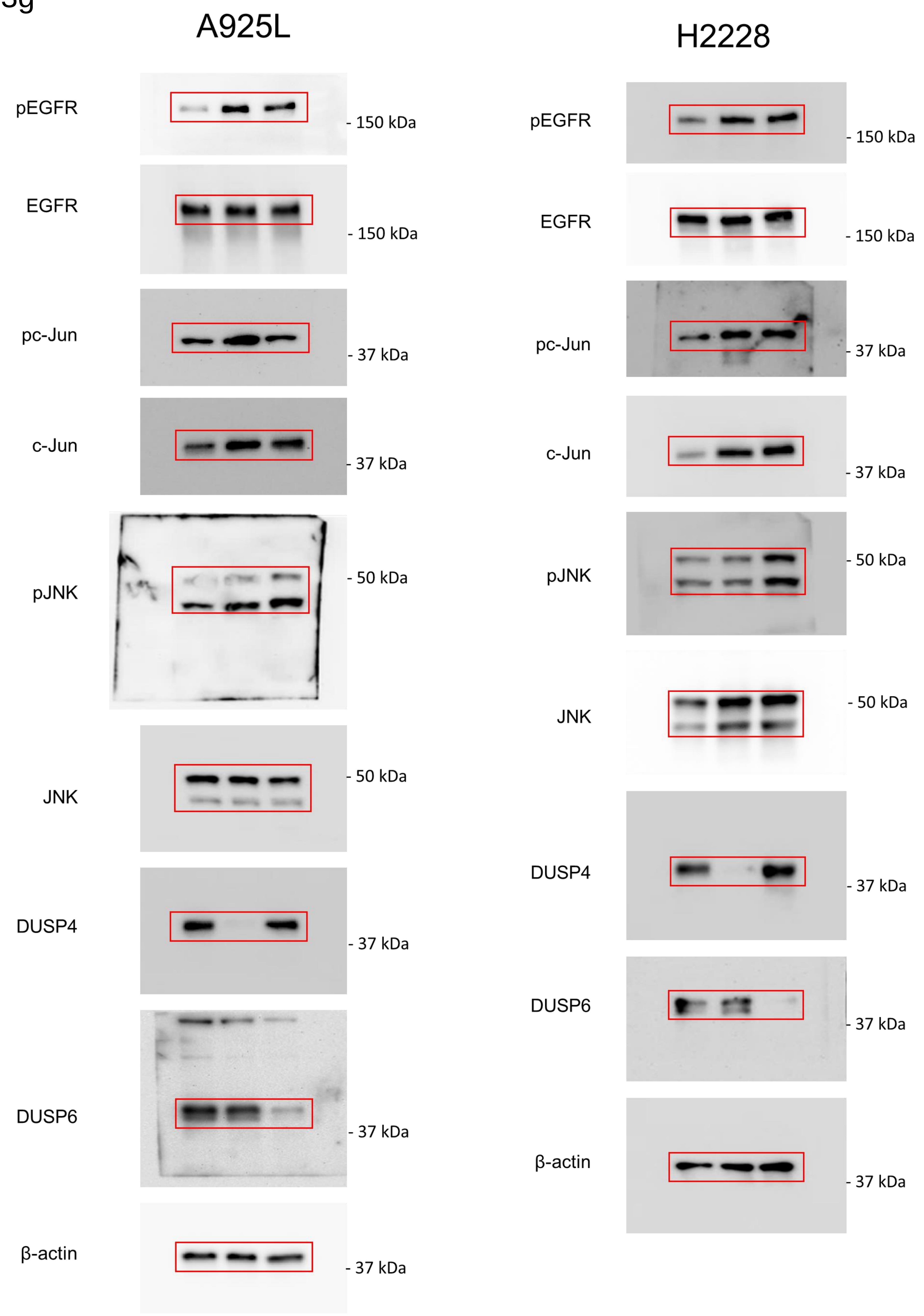

Sup.Fig.18

Fig.4b

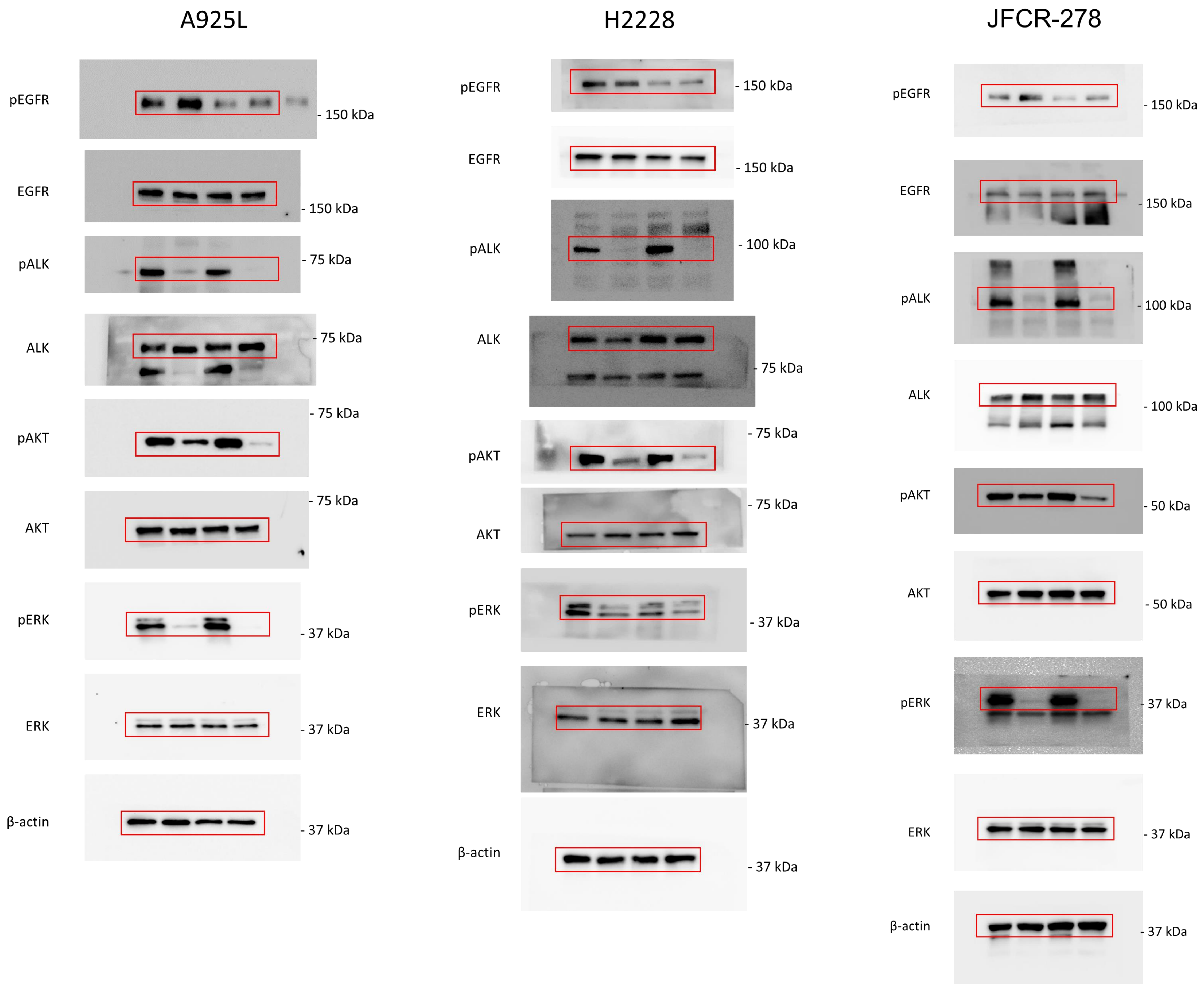

Sup.Fig.18

Fig.4d

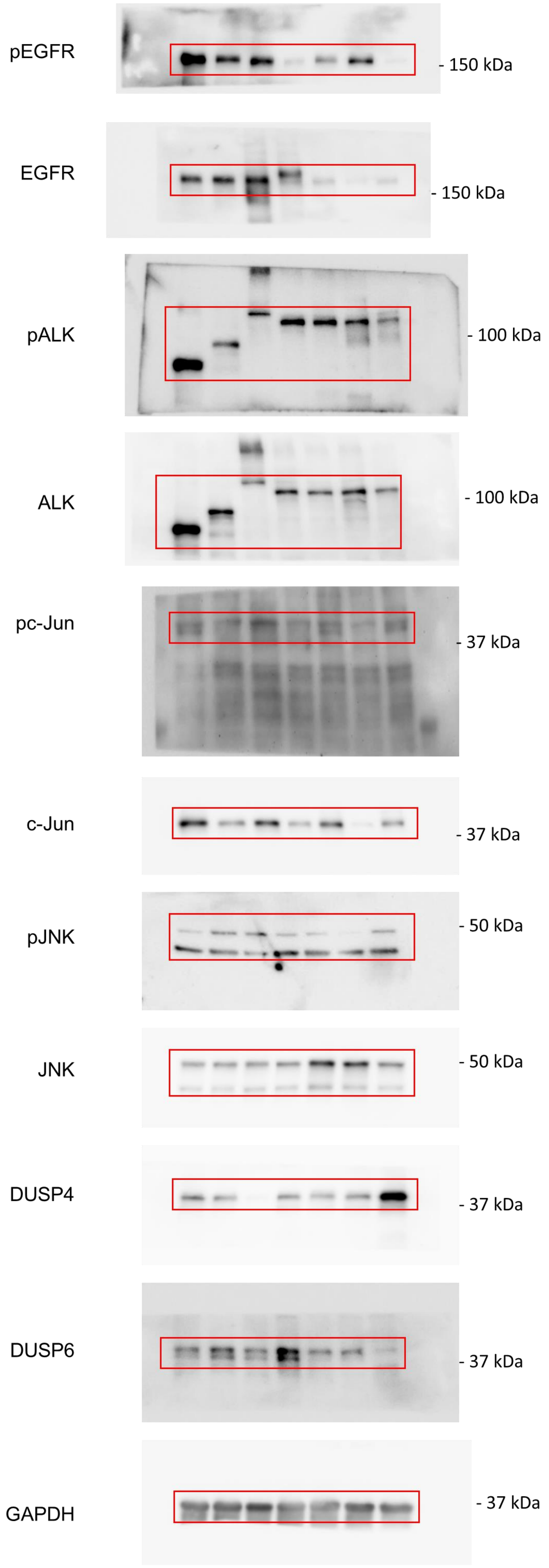

Sup.Fig.18

Fig.5b

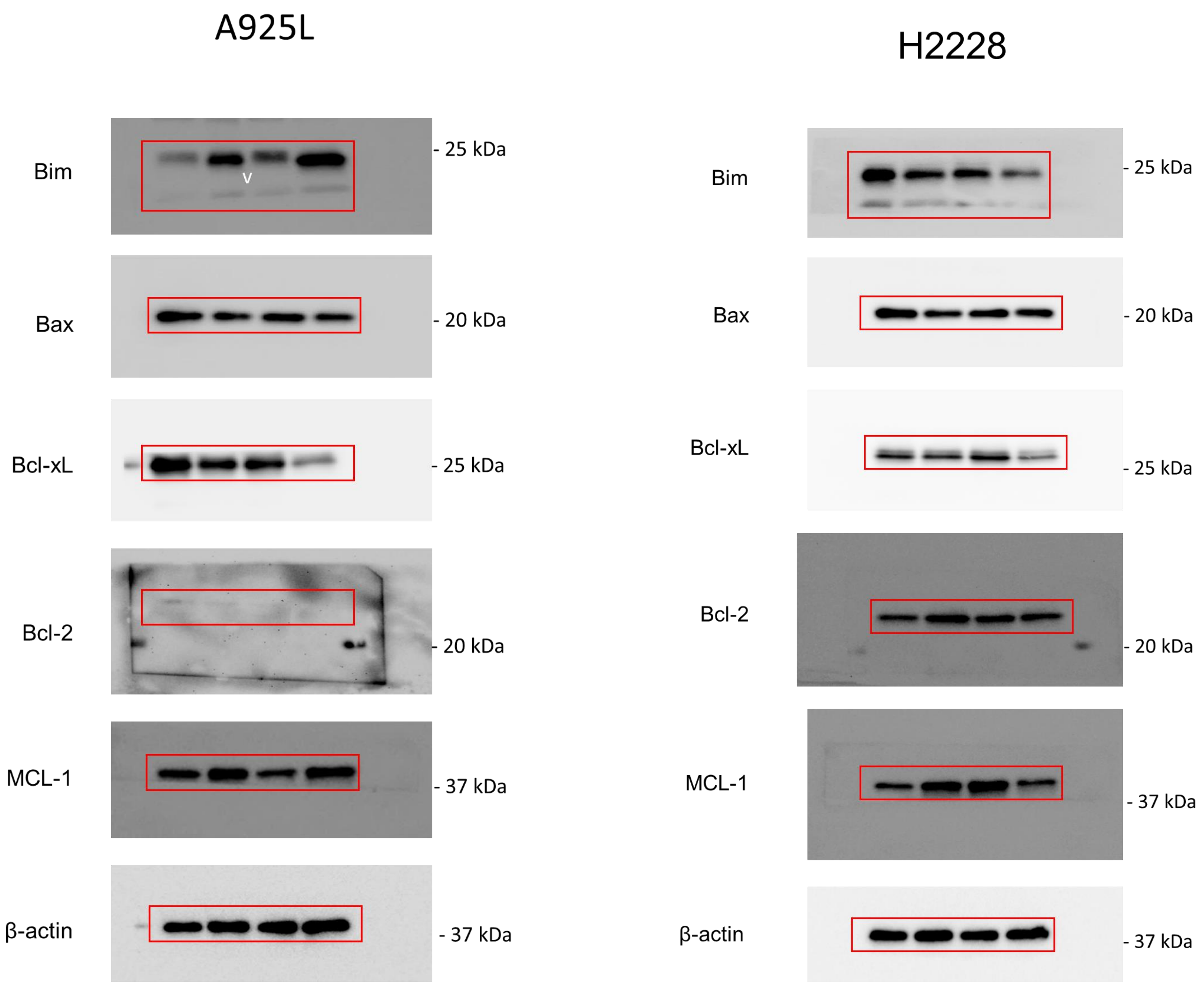

Fig.5d

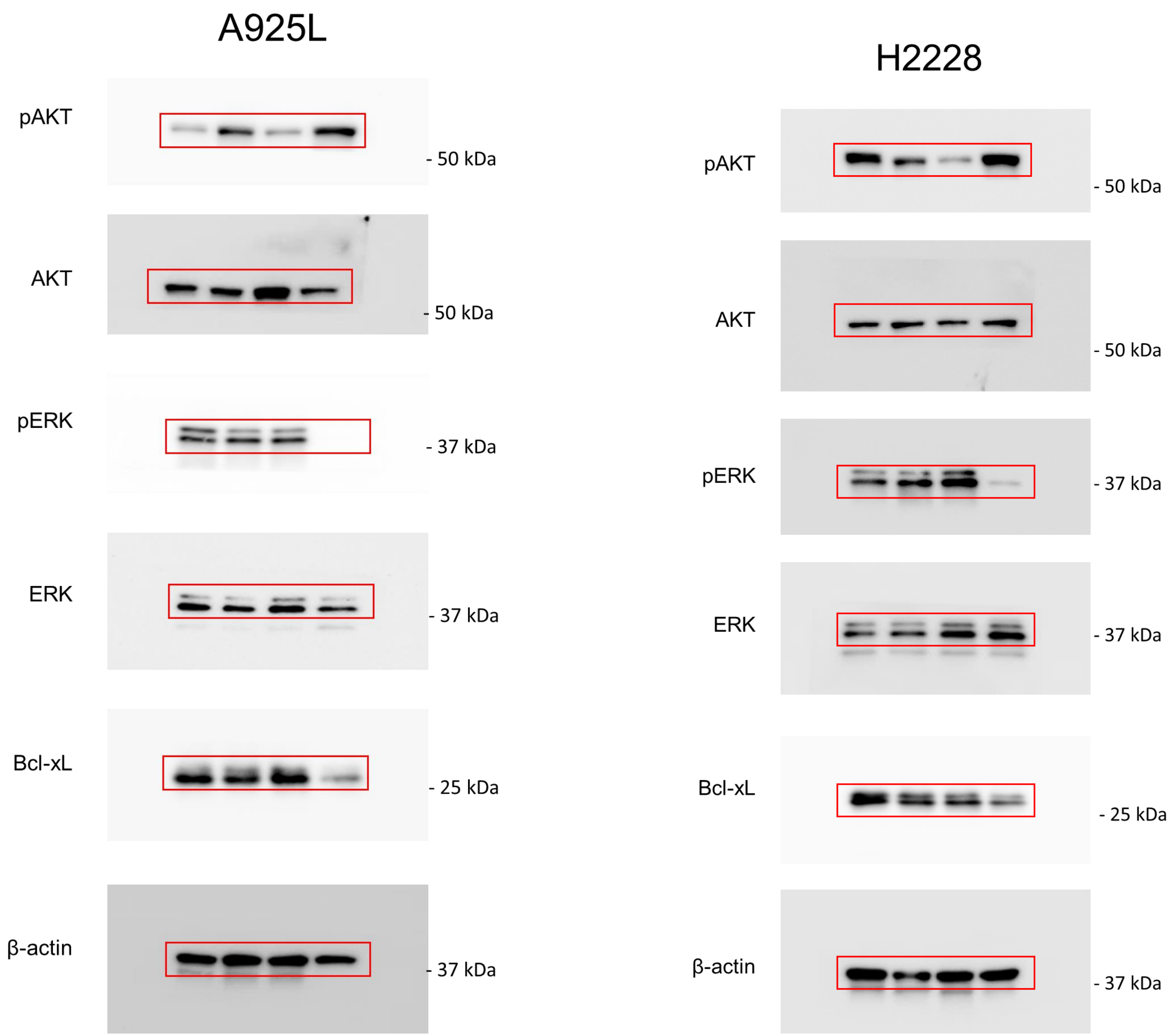

Sup.Fig.18

Fig.5e

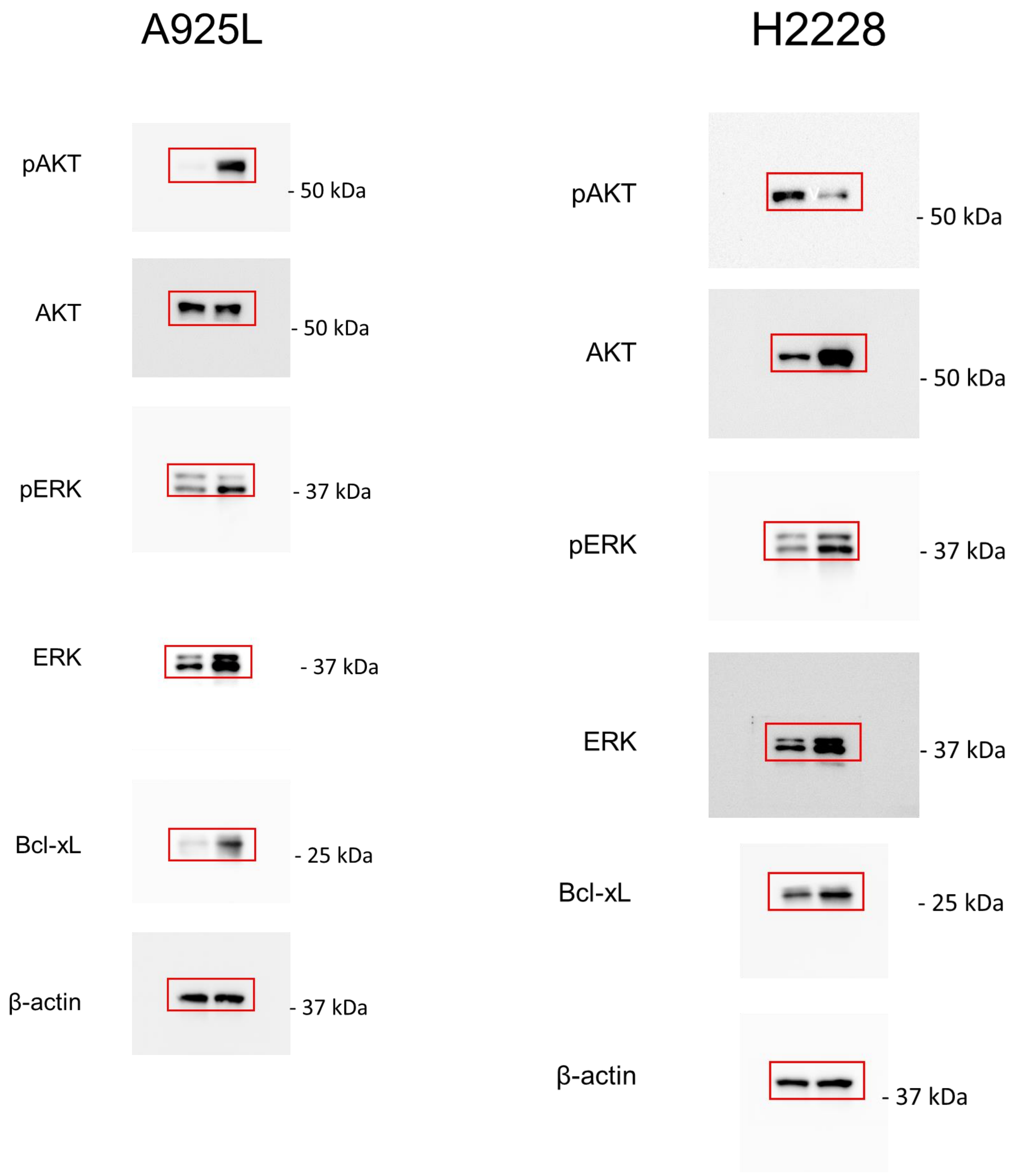

Sup.Fig.10b

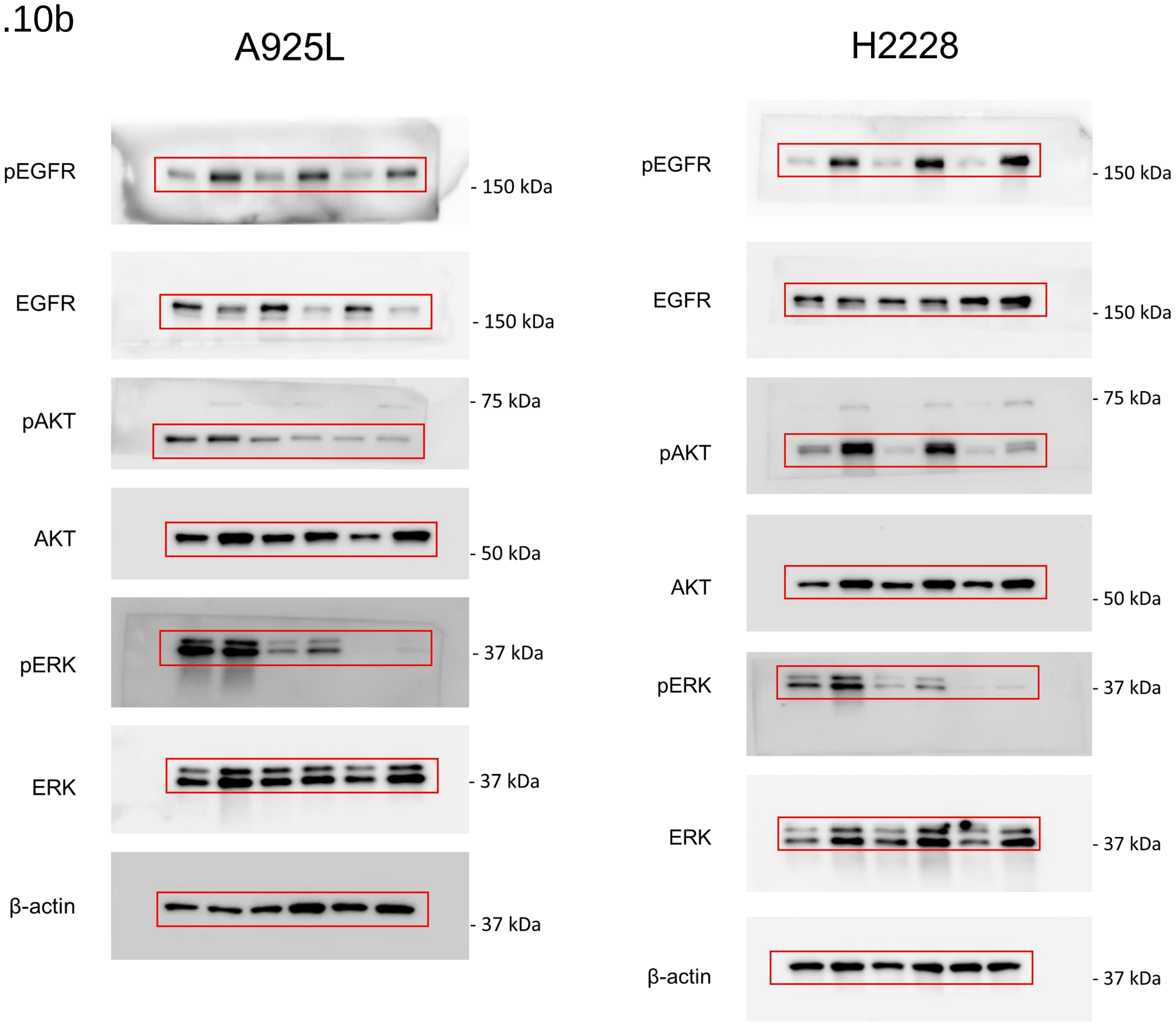

Sup.Fig.18

Sup.Fig.11d

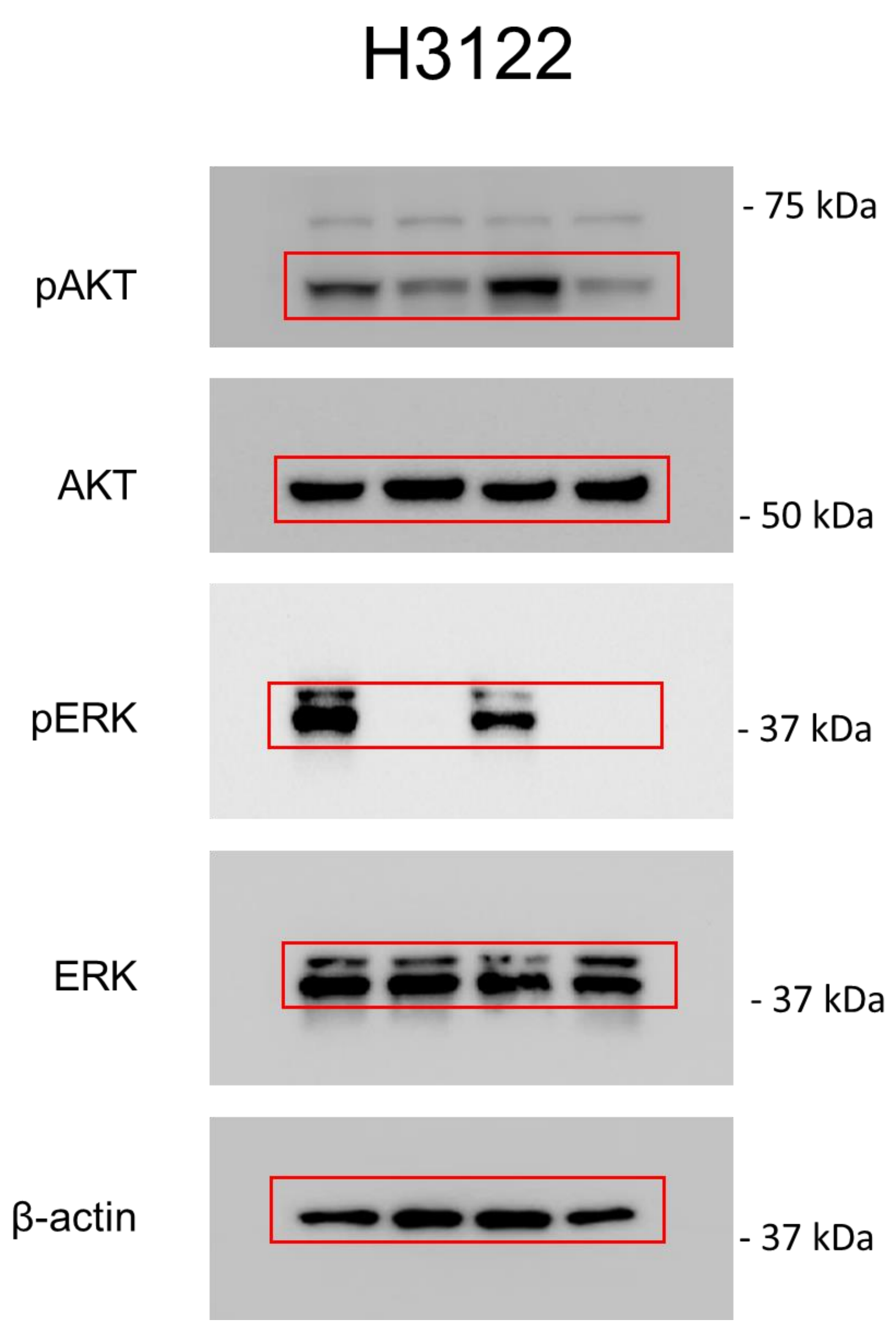

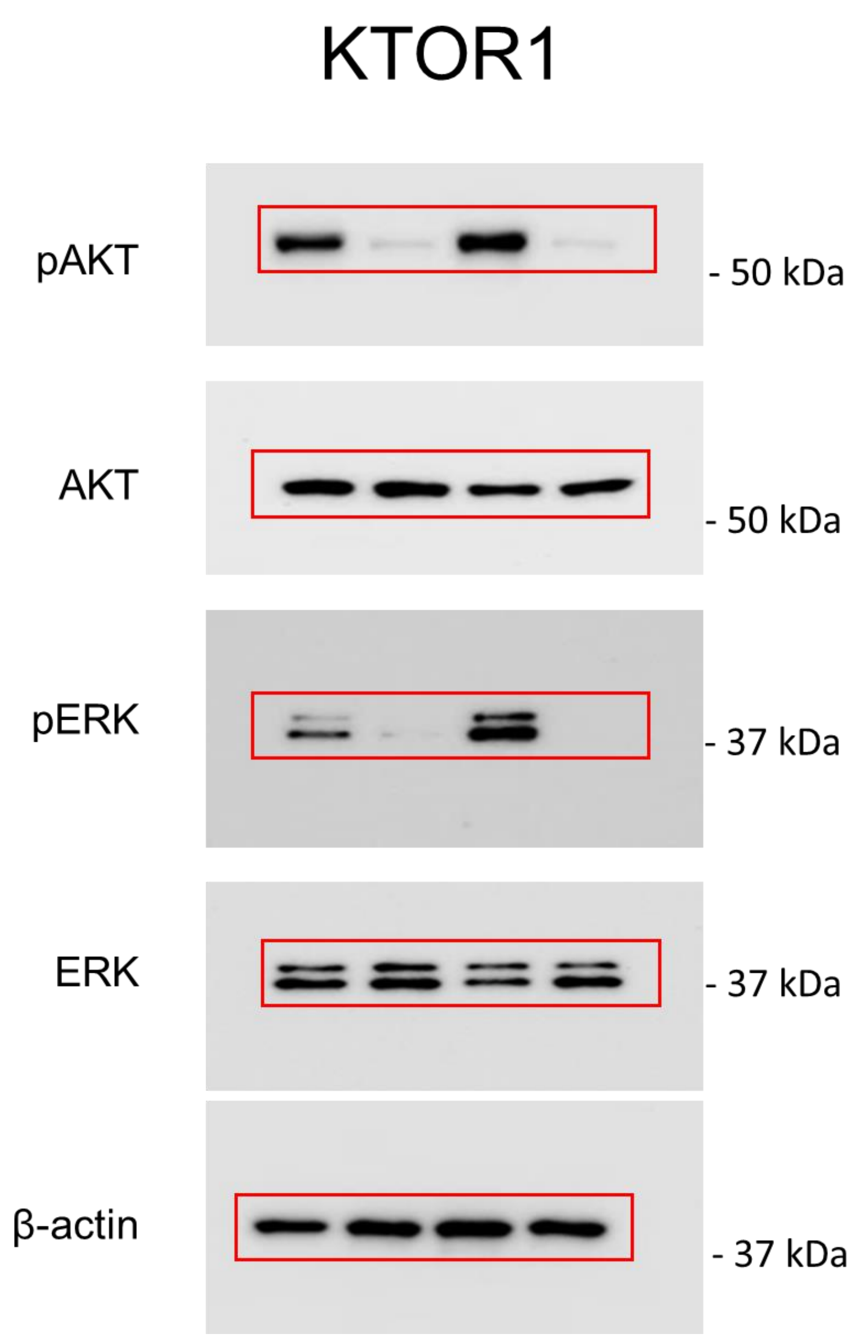

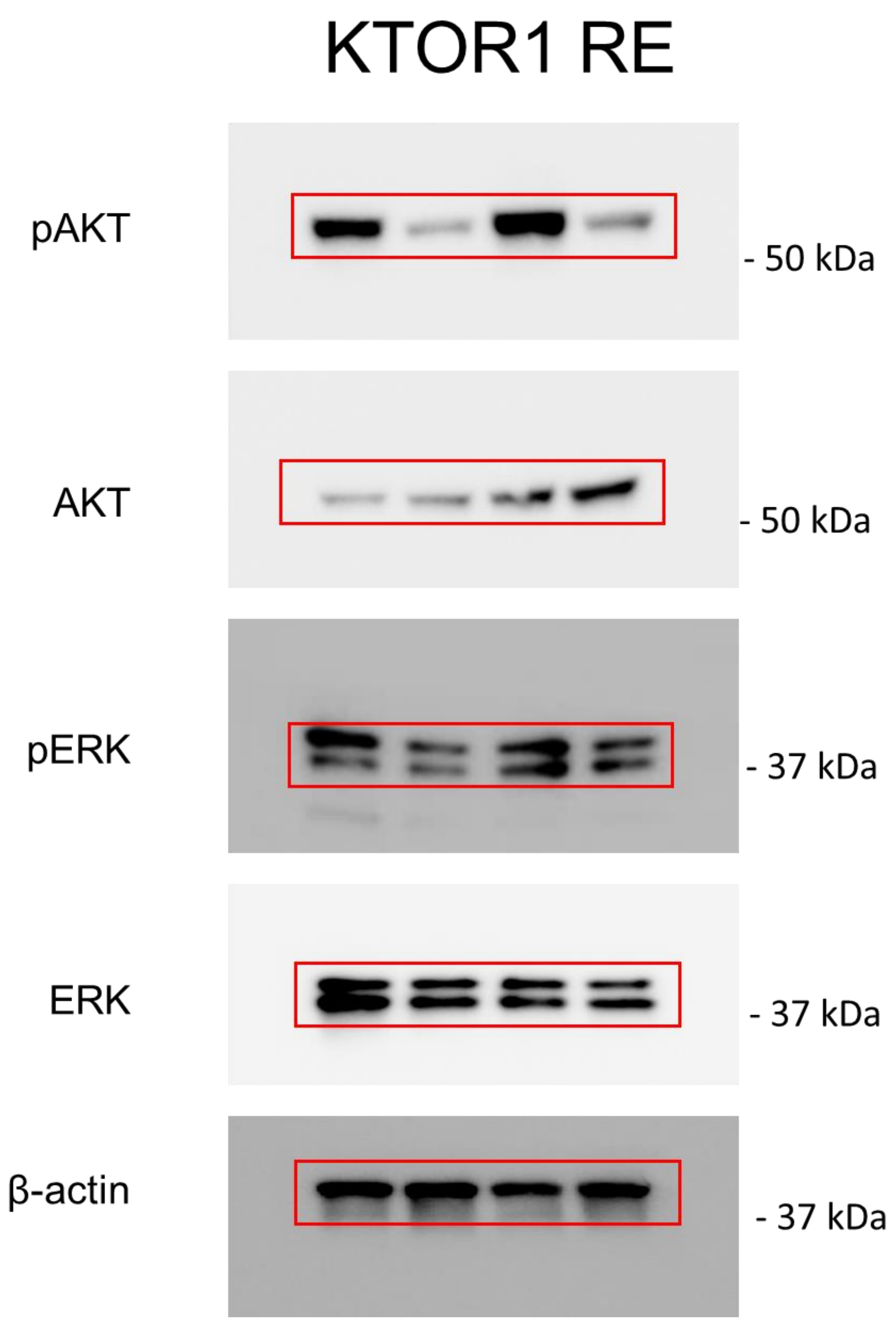

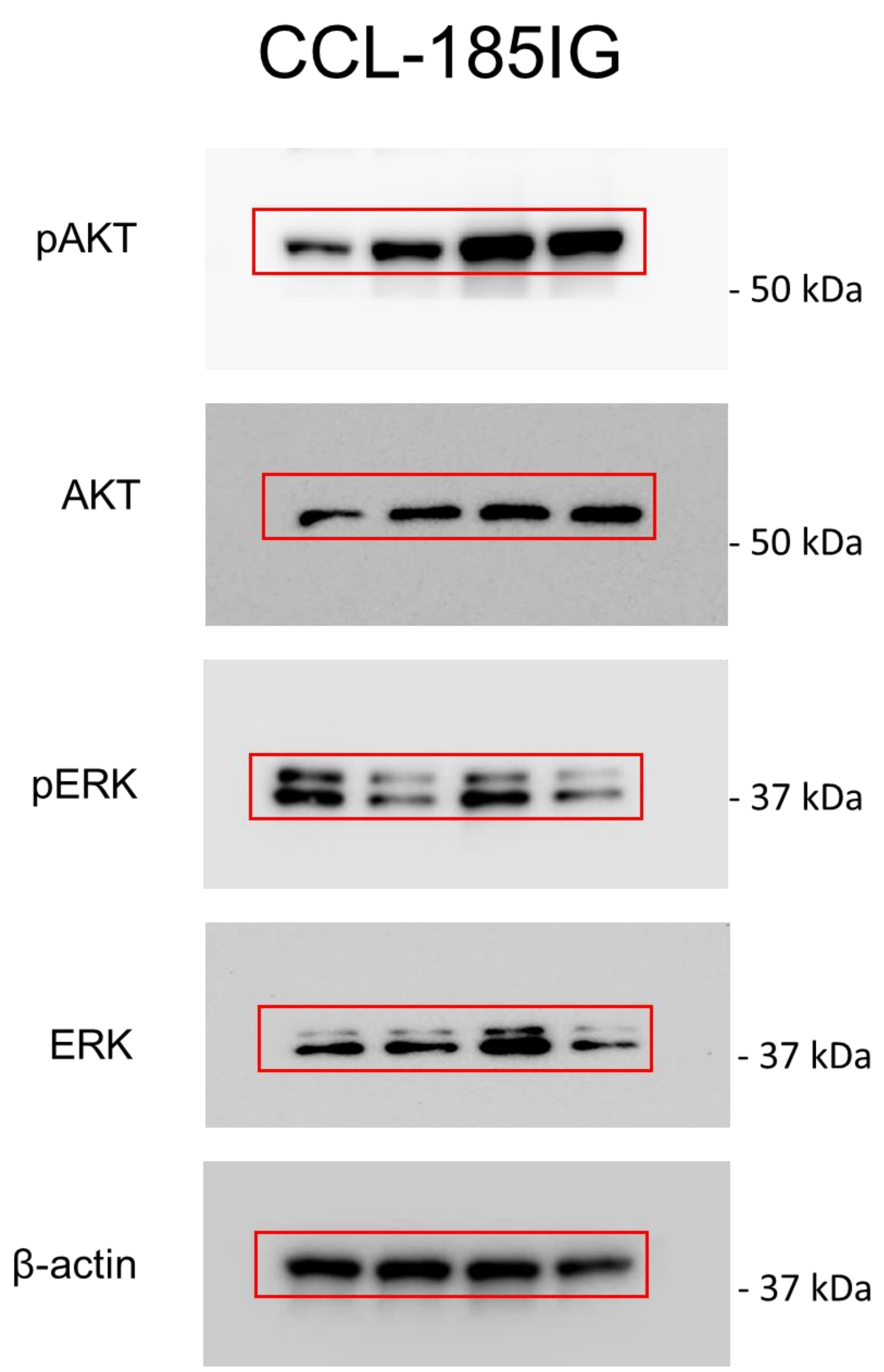

Sup.Fig.18

Sup.Fig.14c

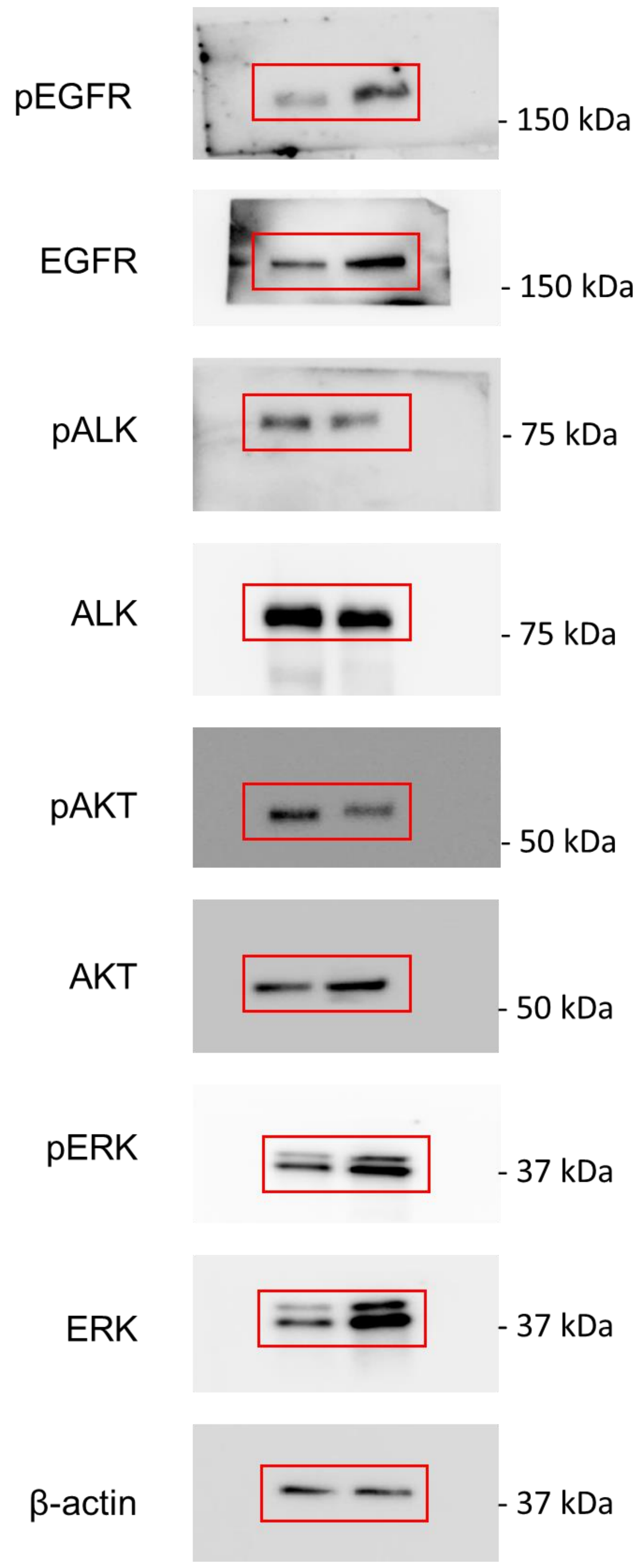

Sup.Fig.14f

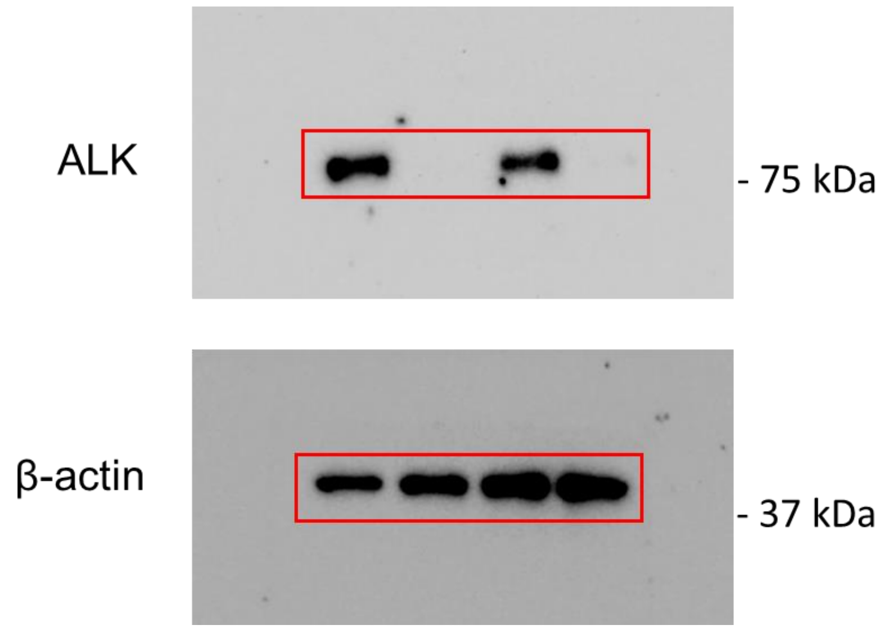

Sup.Fig.14h

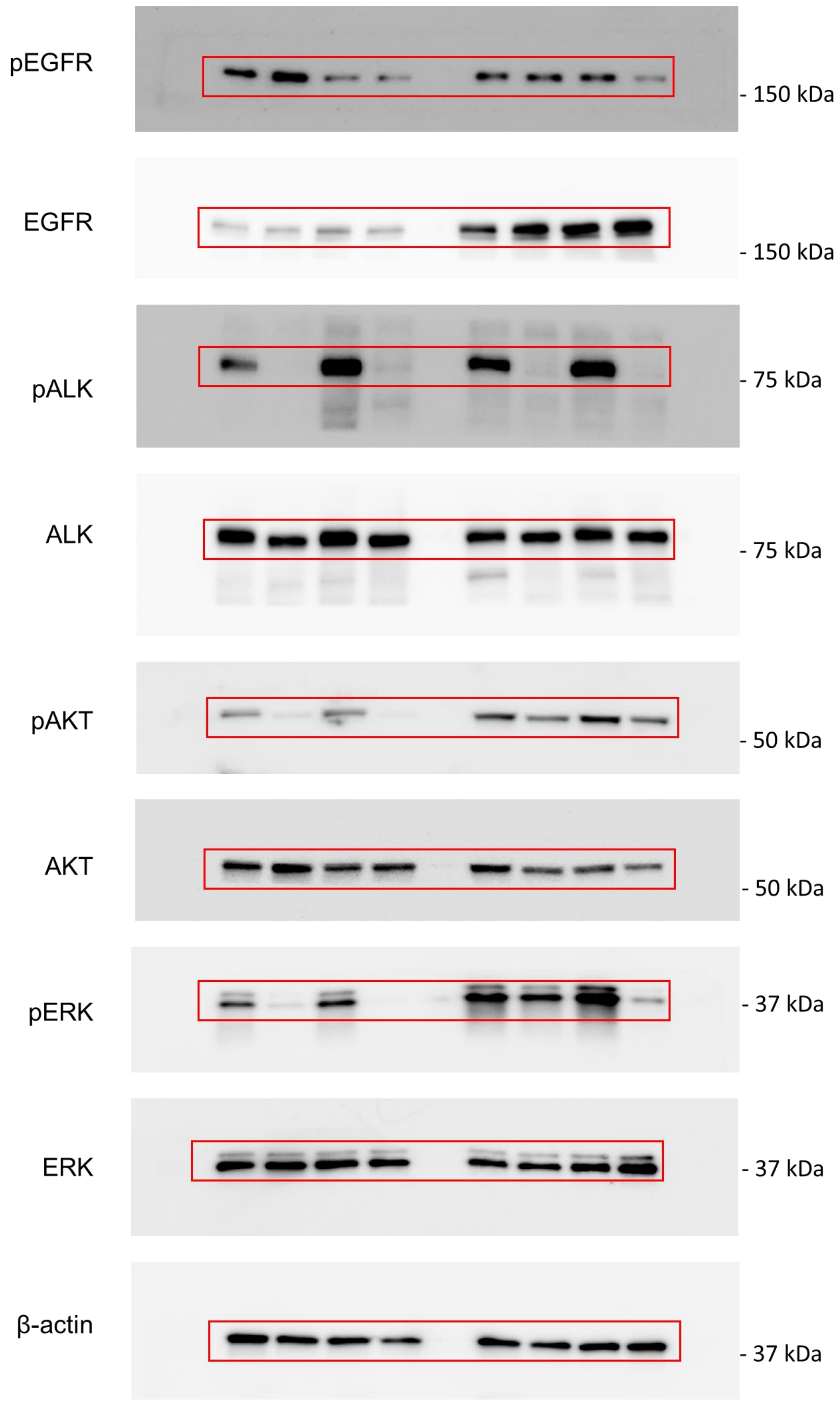

Supplementary Figure 18. Uncropped blots for main figures. Blots shown in the main article are depicted by boxed regions in each of associated uncropped scans

Supplementary Table 1. Upregulated 253 genes

Name
5-hydroxytryptamine receptor 1D(HTR1D)
ABL proto-oncogene 2, non-receptor tyrosine kinase(ABL2)
actin filament associated protein 1 like 2(AFAP1L2)
adhesion molecule with Ig like domain 2(AMIGO2)
A-kinase anchoring protein 12(AKAP12)
AKT serine/threonine kinase 3(AKT3)
aldehyde dehydrogenase 1 family member A3(ALDH1A3)
aldehyde oxidase 1(AOX1)
angiomotin like 1(AMOTL1)
angiomotin like 2(AMOTL2)
ankyrin repeat domain 1(ANKRD1)
ankyrin repeat domain 13A(ANKRD13A)
ankyrin repeat domain 52(ANKRD52)
annexin A3(ANXA3)
annexin A8 like 1(ANXA8L1)
annexin A8(ANXA8)
anosmin 1(ANOS1)
armadillo repeat containing X-linked 2(ARMCX2)
aryl hydrocarbon receptor nuclear translocator 2(ARNT2)
atonal bHLH transcription factor 8(ATOH8)
ATPase phospholipid transporting 8B1(ATP8B1)
BicC family RNA binding protein 1(BICC1)
bile acid-CoA:amino acid N-acyltransferase(BAAT)
BMP and activin membrane bound inhibitor(BAMBI)
BRCA1 interacting helicase 1(BRIP1)
cadherin 1(CDH1)
calcium voltage-gated channel auxiliary subunit gamma 4(CACNG4)
cardiotrophin like cytokine factor 1(CLCF1)
caveolae associated protein 1(CAVIN1)
CD24 molecule(CD24)
CD274 molecule(CD274)
cell adhesion molecule 1(CADM1)
cellular communication network factor 1(CCN1)
cellular communication network factor 2(CCN2)
CEP295 N-terminal like(CEP295NL)
cerebral endothelial cell adhesion molecule(CERCAM)
chemerin chemokine-like receptor 2(CMKLR2)
chloride intracellular channel 3(CLIC3)
chloride intracellular channel 5(CLIC5)
ciliogenesis and planar polarity effector complex subunit 1(CPLANE1)
claudin 1(CLDN1)
coagulation factor III, tissue factor(F3)
coiled-coil domain containing 80(CCDC80)
collagen type I alpha 1 chain(COL1A1)
collagen type IV alpha 4 chain(COL4A4)
collagen type XII alpha 1 chain(COL12A1)
collagen type XVII alpha 1 chain(COL17A1)
complement component 4 binding protein beta(C4BPB)
cut like homeobox 1(CUX1)
C-X-C motif chemokine ligand 16(CXCL16)
CXXC finger protein 5(CXXC5)

Name
cyclin dependent kinase 14(CDK14)
cystatin E/M(CST6)
cysteine rich transmembrane BMP regulator 1(CRIM1)
cytohesin 3(CYTH3)
DAB adaptor protein 2(DAB2)
death associated protein kinase 1(DAPK1)
dedicator of cytokinesis 5(DOCK5)
DExD/H-box 60 like(DDX60L)
diacylglycerol kinase delta(DGKD)
diaphanous related formin 3(DIAPH3)
dickkopf WNT signaling pathway inhibitor 1(DKK1)
discoidin, CUB and LCCL domain containing 1(DCBLD1)
dishevelled associated activator of morphogenesis 1(DAAM1)
DnaJ heat shock protein family (Hsp40) member B2(DNAJB2)
dual specificity phosphatase 1(DUSP1)
dystrobrevin alpha(DTNA)
ectonucleotide pyrophosphatase/phosphodiesterase family member 5(ENPP5)
EGF like repeats and discoidin domains 3(EDIL3)
ELOVL fatty acid elongase 7(ELOVL7)
endothelin 1(EDN1)
epithelial stromal interaction 1(EPSTI1)
erb-b2 receptor tyrosine kinase 2(ERBB2)
family with sequence similarity 13 member B(FAM13B)
F-box and leucine rich repeat protein 2(FBXL2)
FERM domain containing 3(FRMD3)
FERM domain containing 6(FRMD6)
FERM domain containing kindlin 2(FERMT2)
fibrillin 1(FBN1)
filamin A interacting protein 1 like(FILIP1L)
filamin B(FLNB)
follistatin like 1(FSTL1)
follistatin like 3(FSTL3)
forkhead box N3(FOXN3)
G protein-coupled receptor 161(GPR161)
G protein-coupled receptor 176(GPR176)
G protein-coupled receptor 87(GPR87)
G protein-coupled receptor class C group 5 member A(GPRC5A)
GABA type A receptor associated protein like 1(GABARAPL1)
gap junction protein beta 3(GJB3)
GLI pathogenesis related 1(GLIPR1)
GLIS family zinc finger 3(GLIS3)
golgi associated kinase 1B(GASK1B)
growth arrest and DNA damage inducible beta(GADD45B)
hedgehog acyltransferase(HHAT)
heparan sulfate proteoglycan 2(HSPG2)
heparin binding EGF like growth factor(HBEGF)
HIVEP zinc finger 1(HIVEP1)
inhibitor of DNA binding 2(ID2)
insulin like growth factor binding protein 7(IGFBP7)
interferon alpha inducible protein 27(IFI27)
interferon induced protein with tetratricopeptide repeats 2(IFIT2)

Name
interleukin 2 receptor subunit gamma(IL2RG)
interleukin 32(IL32)
Janus kinase 1(JAK1)
junction plakoglobin(JUP)
keratin 17(KRT17)
keratin 7(KRT7)
keratin 80(KRT80)
keratin 81(KRT81)
KIAA0408(KIAA0408)
killer cell immunoglobulin like receptor, two Ig domains and long cytoplasmic tail 4(KIR2DL4)
KLF transcription factor 7(KLF7)
lactamase beta(LACTB)
laminin subunit gamma 2(LAMC2)
latent transforming growth factor beta binding protein 2(LTBP2)
LBH regulator of WNT signaling pathway(LBH)
LHFPL tetraspan subfamily member 6(LHFPL6)
LIM domain and actin binding 1(LIMA1)
lysine demethylase 3A(KDM3A)
lysyl oxidase like 1(LOXL1)
lysyl oxidase(LOX)
Mab-21 domain containing 2(MB21D2)
MARVEL domain containing 1(MARVELD1)
matrix metallopeptidase 24(MMP24)
melanoma cell adhesion molecule(MCAM)
melanotransferrin(MELTF)
methionine sulfoxide reductase B3(MSRB3)
microRNA 198(MIR198)
microRNA 4728(MIR4728)
microRNA 5572(MIR5572)
microRNA 614(MIR614)
microRNA 6756(MIR6756)
microRNA 6827(MIR6827)
microRNA 936(MIR936)
microtubule associated monooxygenase, calponin and LIM domain containing 3(MICAL3)
monoglyceride lipase(MGLL)
MSANTD3-TMEFF1 readthrough(MSANTD3-TMEFF1)
muscle RAS oncogene homolog(MRAS)
MYB proto-oncogene like 1(MYBL1)
myocardin related transcription factor A(MRTFA)
myosin heavy chain 9(MYH9)
myosin IE(MYO1E)
myosin light chain 9(MYL9)
myosin light chain kinase(MYLK)
NALCN channel auxiliary factor 1(NALF1)
N-deacetylase and N-sulfotransferase 1(NDST1)
neogenin 1(NEO1)
nephronectin(NPNT)
nerve growth factor(NGF)
netrin 4(NTN4)
neuregulin 1(NRG1)
neuropeptide Y receptor Y4(NPY4R)

Supplementary Table 1. Upregulated 253 genes

Name
neutrophil cytosolic factor 2(NCF2)
nexilin F-actin binding protein(NEXN)
NF2, moesin-ezrin-radixin like (MERLIN) tumor suppressor(NF2)
NK3 homeobox 1(NKX3-1)
notch 2 N-terminal like A(NOTCH2NLA)
notch receptor 2(NOTCH2)
nucleic acid binding protein 1(NABP1)
par-3 family cell polarity regulator beta(PARD3B)
phosphatidylinositol specific phospholipase C X domain containing 2(PLCXD2)
phosphodiesterase 5A(PDE5A)
piggyBac transposable element derived 5(PGBD5)
pleckstrin homology domain containing O1(PLEKHO1)
pleckstrin homology like domain family B member 2(PHLDB2)
podocalyxin like(PODXL)
polo like kinase 2(PLK2)
potassium voltage-gated channel subfamily H member 1(KCNH1)
pregnancy specific beta-1-glycoprotein 1(PSG1)
pregnancy specific beta-1-glycoprotein 5(PSG5)
pregnancy specific beta-1-glycoprotein 9(PSG9)
pro-apoptotic WT1 regulator(PAWR)
programmed cell death 1 ligand 2(PDCD1LG2)
proliferation and apoptosis adaptor protein 15(PEA15)
proline and serine rich 2(PROSER2)
proline rich 16(PRR16)
prostaglandin F2 receptor inhibitor(PTGFRN)
prostate transmembrane protein, androgen induced 1(PMEPA1)
protein phosphatase 1 regulatory subunit 9A(PPP1R9A)
protein tyrosine phosphatase non-receptor type 21(PTPN21)
protein tyrosine phosphatase receptor type K(PTPRK)
PX domain containing 1(PXDC1)
RAB11 family interacting protein 1(RAB11FIP1)
ras homolog family member B(RHOB)
reversion inducing cysteine rich protein with kazal motifs(RECK)
Rho GTPase activating protein 20(ARHGAP20)
Rho GTPase activating protein 42(ARHGAP42)
Rho related BTB domain containing 1(RHOBTB1)
ribosomal modification protein rimK like family member B(RIMKLB)
ribosomal protein L39 pseudogene 5(RPL39P5)
ring finger protein 144B(RNF144B)
RNA binding motif single stranded interacting protein 3(RBMS3)
RUN and SH3 domain containing 2(RUSC2)
sacsin molecular chaperone(SACS)
sarcospan(SSPN)
sciellin(SCEL)
secreted protein acidic and cysteine rich(SPARC)
semaphorin 3C(SEMA3C)
semaphorin 3E(SEMA3E)
semaphorin 7A (John Milton Hagen blood group)(SEMA7A)
serpin family E member 1(SERPINE1)
SERTA domain containing 4(SERTAD4)
shroom family member 3(SHROOM3)

Name
SKI like proto-oncogene(SKIL)
SMAD family member 7(SMAD7)
snail family transcriptional repressor 2(SNAI2)
SOGA family member 3(SOGA3)
solute carrier family 26 member 2(SLC26A2)
solute carrier family 44 member 2(SLC44A2)
solute carrier family 66 member 1 like(SLC66A1L)
ST6 N-acetylgalactosaminide alpha-2,6-sialyltransferase 5(ST6GALNAC5)
StAR related lipid transfer domain containing 13(STARD13)
sterile alpha motif domain containing 12(SAMD12)
synaptopodin(SYNPO)
synaptotagmin 1(SYT1)
synaptotagmin 15(SYT15)
syntaxin 11(STX11)
t-complex 11 like 1(TCP11L1)
tensin 4(TNS4)
tetraspanin 2(TSPAN2)
tetratricopeptide repeat domain 9(TTC9)
TGFB2 overlapping transcript 1(TGFB2-OT1)
thrombospondin 1(THBS1)
thrombospondin type 1 domain containing 4(THSD4)
thymocyte selection associated high mobility group box(TOX)
TIMP metallopeptidase inhibitor 2(TIMP2)
tissue factor pathway inhibitor 2(TFPI2)
TLE family member 4, transcriptional corepressor(TLE4)
transforming growth factor beta 2(TGFB2)
transforming growth factor beta induced(TGFBI)
transgelin(TAGLN)
transglutaminase 5(TGM5)
transmembrane 131 like(TMEM131L)
transmembrane protein 130(TMEM130)
transmembrane protein 139(TMEM139)
transmembrane protein adipocyte associated 1(TPRA1)
transmembrane protein with EGF like and two follistatin like domains 1(TMEFF1)
trio Rho guanine nucleotide exchange factor(TRIO)
tripartite motif containing 29(TRIM29)
tropomyosin 1(TPM1)
tubulointerstitial nephritis antigen like 1(TINAGL1)
tuftelin 1(TUFT1)
tumor protein p53 inducible protein 3(TP53I3)
ubiquitin specific peptidase 11(USP11)
UDP-glucose ceramide glucosyltransferase(UGCG)
utrophin(UTRN)
V-set domain containing T cell activation inhibitor 1(VTCN1)
V-set immunoregulatory receptor(VSIR)
WD repeat and FYVE domain containing 1(WDFY1)
WW and C2 domain containing 1(WWC1)
zinc finger NFX1-type containing 1(ZNFX1)
zinc finger protein 704(ZNF704)

Supplementary Table 2. Characteristics of patients

Characteristics	All patients (n = 26)	high EGFR expression (n = 10, 38.5%)	low EGFR expression (n = 16, 61.5%)	*p*-value
Age				
Median (range)	70.5 (42-85)	69 (42-79)	71.5 (53-85)	0.09
Sex				
Male	9 (34.7%)	5 (50.0%)	4 (25.0%)	0.23
Female	17 (65.4%)	5 (50.5%)	75 (5.0%)	
Stage				
III	5 (19.2%)	2 (20.0%)	3 (18.8%)	0.21
IV	13 (50.0%)	7 (70.0%)	6 (37.5%)	
Recurrence	5 (19.2%)	1 (10.0%)	7 (43.8%)	
Smoking status				
Current/Former	11 (42.3%)	6 (60.0%)	5 (31.2%)	0.23
Never	15 (57.7%)	4 (40.0%)	11 (68.8%)	
phospho-EGFR				
high expression	11 (42.3%)	6 (60.0%)	5 (31.2%)	0.23
low expression	15 (57.7%)	4 (40.0%)	11 (68.8%)	

Supplementary Table 3: Details of the antibodies used in this study.

Antibodies	Dilution	Company	Catalog#
p-EGFR (Tyr1068)	1 : 1000	Cell Signaling Technology	3777
p-AKT (Ser473)	1 : 1000	Cell Signaling Technology	4060
t-AKT	1 : 1000	Cell Signaling Technology	9272
p-ERK1/2 (Thr202/Tyr204)	1 : 1000	Cell Signaling Technology	4370
t-ERK1/2	1 : 1000	Cell Signaling Technology	4695
p-c-Jun (Ser63)	1 : 1000	Cell signaling Technology	2361
c-Jun (60A8)	1 : 1000	Cell signaling Technology	9165
p-SAPK/JNK (Thr183/Tyr185)	1 : 1000	Cell signaling Technology	4668
JNK	1 : 1000	Cell signaling Technology	9252
β-actin	1 : 1000	Cell signaling Technology	4970
DUSP4	1 : 1000	Cell signaling Technology	5149
DUSP6	1 : 1000	Cell signaling Technology	3058
p-ALK (Tyr1604)	1 : 1000	Cell signaling Technology	3341
ALK	1 : 1000	Cell signaling Technology	3633
BIM	1 : 1000	Cell signaling Technology	2933
BAX	1 : 1000	Cell signaling Technology	2772
Bcl-xL	1 : 1000	Cell signaling Technology	2764
Bcl-2	1 : 1000	Cell signaling Technology	15071
Mcl-1	1 : 1000	Cell signaling Technology	5453
GAPDH	1 : 1000	Cell signaling Technology	2118
t-EGFR	1 : 1000	R&D Systems	AF231

Supplementary Table 4: Primer sequences for PCR

Gene	Primer sequence
HB-EGF	Forward: 5′ - ATGAAGCTGCTGCCGTCGGTG-3′
	Reverse: 5′ - TGGATGCAGTAGTCCTTGTATTTC-3′
EGFR	Forward: 5′ - CTTCTTAAAGACCATCCAGG-3′
	Reverse: 5′ - TTTCTGGCAGTTCTCCTCTC-3′
GAPDH	Forward: 5′ - GTCTCCTCTGACTTCAACAGCG-3′
	Reverse: 5′ - ACCACCCTGTTGCTGTAGCCAA-3′
