# Supplementary material for: Adaptive resistance to lorlatinib via EGFR signaling in ALK-rearranged lung cancer
Source: NPJ Precis Oncol. 2023 Jan 26;7:12. doi: 10.1038/s41698-023-00350-7 (PMC9879975; doi:10.1038/s41698-023-00350-7)
Supplement: Supplementary file 2 — REPORTING SUMMARY [file 41698_2023_350_MOESM2_ESM.pdf]

nature portfolio

Corresponding author(s): Tadaaki Yamada

Last updated by author(s): Nov 30, 2022

Reporting Summary

Nature Portfolio wishes to improve the reproducibility of the work that we publish. This form provides structure for consistency and transparency in reporting. For further information on Nature Portfolio policies, see our Editorial Policies and the Editorial Policy Checklist.

Statistics

For all statistical analyses, confirm that the following items are present in the figure legend, table legend, main text, or Methods section.

n/a	Confirmed	
☐	☒	The exact sample size (*n*) for each experimental group/condition, given as a discrete number and unit of measurement
☐	☒	A statement on whether measurements were taken from distinct samples or whether the same sample was measured repeatedly
☐	☒	The statistical test(s) used AND whether they are one- or two-sided<br>*Only common tests should be described solely by name; describe more complex techniques in the Methods section.*
☒	☐	A description of all covariates tested
☐	☒	A description of any assumptions or corrections, such as tests of normality and adjustment for multiple comparisons
☐	☒	A full description of the statistical parameters including central tendency (e.g. means) or other basic estimates (e.g. regression coefficient) AND variation (e.g. standard deviation) or associated estimates of uncertainty (e.g. confidence intervals)
☐	☒	For null hypothesis testing, the test statistic (e.g. *F*, *t*, *r*) with confidence intervals, effect sizes, degrees of freedom and *P* value noted<br>*Give P values as exact values whenever suitable.*
☒	☐	For Bayesian analysis, information on the choice of priors and Markov chain Monte Carlo settings
☒	☐	For hierarchical and complex designs, identification of the appropriate level for tests and full reporting of outcomes
☒	☐	Estimates of effect sizes (e.g. Cohen's *d*, Pearson's *r*), indicating how they were calculated

Our web collection on statistics for biologists contains articles on many of the points above.

Software and code

Policy information about availability of computer code

Data collection	No software was used.
Data analysis	GraphPad Prism Ver. 8.0 (GraphPad Software, Inc., San Diego, CA, USA)<br>ImageJ/FIJI (https://fiji.sc/)

For manuscripts utilizing custom algorithms or software that are central to the research but not yet described in published literature, software must be made available to editors and reviewers. We strongly encourage code deposition in a community repository (e.g. GitHub). See the Nature Portfolio guidelines for submitting code & software for further information.

Data

Policy information about availability of data

All manuscripts must include a data availability statement. This statement should provide the following information, where applicable:

- Accession codes, unique identifiers, or web links for publicly available datasets
- A description of any restrictions on data availability
- For clinical datasets or third party data, please ensure that the statement adheres to our policy

The authors declare that all data supporting the findings of this study are available within the paper and its supplementary information files

Human research participants

Policy information about studies involving human research participants and Sex and Gender in Research.

Reporting on sex and gender	n/a
Population characteristics	ALK-rearranged tumor specimens were obtained from 26 patients with NSCLC at University Hospital, Kyoto Prefectural University of Medicine (Kyoto, Japan), Japanese Red Cross Kyoto Daiichi Hospital (Kyoto, Japan), and Japanese Red Cross Kyoto Daini Hospital (Kyoto, Japan) prior to treatment with alectinib.
Recruitment	All patients were participants in Institutional Review Board of each Hospitals –approved studies.
Ethics oversight	All patients were participants in Institutional Review Board of each Hospital –approved studies and all provided written informed consent.

Note that full information on the approval of the study protocol must also be provided in the manuscript.

Field-specific reporting

Please select the one below that is the best fit for your research. If you are not sure, read the appropriate sections before making your selection.

☒ Life sciences ☐ Behavioural & social sciences ☐ Ecological, evolutionary & environmental sciences

For a reference copy of the document with all sections, see nature.com/documents/nr-reporting-summary-flat.pdf

Life sciences study design

All studies must disclose on these points even when the disclosure is negative.

Sample size	This research mainly targets for experimental research.
Data exclusions	No data were excluded from the analysis.
Replication	For in vitro experiments, each experiment was independently performed at least twice.
Randomization	In mice experiments, the mice were transferred to the animal facility at Kyoto Prefectural University of Medicine and randomized once their mean tumor volume reached the each indicated volume.
Blinding	Blinding was not performed in this study.

Reporting for specific materials, systems and methods

We require information from authors about some types of materials, experimental systems and methods used in many studies. Here, indicate whether each material, system or method listed is relevant to your study. If you are not sure if a list item applies to your research, read the appropriate section before selecting a response.

Materials & experimental systems

n/a	Involved in the study
☐	☒ Antibodies
☐	☒ Eukaryotic cell lines
☒	☐ Palaeontology and archaeology
☐	☒ Animals and other organisms
☒	☐ Clinical data
☒	☐ Dual use research of concern

Methods

n/a	Involved in the study
☒	☐ ChIP-seq
☒	☐ Flow cytometry
☒	☐ MRI-based neuroimaging

Antibodies

Antibodies used	p-EGFR (Tyr1068), p-AKT (Ser473), t-AKT, p-ERK1/2 (Thr202/Tyr204), t-ERK1/2, p-c-Jun (Ser63), c-Jun (60A8), p-SAPK/JNK (Thr183/Tyr185), JNK, β-actin, DUSP4, DUSP6, p-ALK (Tyr1604), ALK, BIM, BAX, Bcl-xL, Bcl-2, Mcl-1, GAPDH (Cell Signaling Technology)<br>t-EGFR (R&D systems)

Validation	All antibodies are commercially available and have been validated by the manufacturer.

Eukaryotic cell lines

Policy information about cell lines and Sex and Gender in Research

Cell line source(s)	A925L (EML4–ALK variant 5a, E2: A20) was established from a surgical specimen of the EML4–ALK-positive NSCLC patient kindly provided by Fumihiro Tanaka of the Second Department of Surgery, University of Occupational and Environmental Health, Japan. H2228 (EML4–ALK variant 3a/b E6; A20) and CCL-185IG™ (A549 EML4-ALK+) cells were purchased from the American Type Culture Collection (Manassas, VA, USA). The H3122 human lung adenocarcinoma cell line, with EML4–ALK fusion protein variant 1 (E13; A20), was kindly provided by Dr. Jeffrey A. Engelman of the Massachusetts General Hospital Cancer Center (Boston, MA, USA). The KTOR1 and KTOR1-RE cells with EML4–ALK fusion protein variant 1 were established from patients with ALK-rearranged NSCLC who regularly visited the Kyoto University Hospital. The EML4-ALK-positive NSCLC patient-derived cell line JFCR-278 was established from the EML4-ALK-positive NSCLC patient in Hospital of Japanese Foundation for Cancer Research, Japan
Authentication	Cell lines were authenticated by DNA fingerprinting.
Mycoplasma contamination	Cells were regularly screened for mycoplasma using a MycoAlert Mycoplasma Detection Kit.
Commonly misidentified lines (See ICLAC register)	Commonly misidentified lines were not used in the study.

Animals and other research organisms

Policy information about studies involving animals; ARRIVE guidelines recommended for reporting animal research, and Sex and Gender in Research

Laboratory animals	Five-week-old male C.B-17/Icr-scid/scidJcl mice with severe combined immunodeficiency were obtained from Clea Japan (Tokyo, Japan).
Wild animals	n/a
Reporting on sex	Data are no relevant to the sex of mice used in this study.
Field-collected samples	n/a
Ethics oversight	Mouse experimental protocols were approved by the institutional review board of Kyoto Prefectural University of Medicine (Kyoto, Japan; approval no. M29-529).

Note that full information on the approval of the study protocol must also be provided in the manuscript.
